# Acute liver steatosis translationally controls the epigenetic regulator MIER1 to promote liver regeneration in a study with male mice

**DOI:** 10.1038/s41467-023-37247-9

**Published:** 2023-03-18

**Authors:** Yanhao Chen, Lanlan Chen, Xiaoshan Wu, Yongxu Zhao, Yuchen Wang, Dacheng Jiang, Xiaojian Liu, Tingting Zhou, Shuang Li, Yuda Wei, Yan Liu, Cheng Hu, Ben Zhou, Jun Qin, Hao Ying, Qiurong Ding

**Affiliations:** 1 CAS Key Laboratory of Nutrition, Metabolism and Food Safety, Shanghai Institute of Nutrition and Health, University of Chinese Academy of Sciences, Chinese Academy of Sciences, Shanghai, 200031 P. R. China; 2 School of Pharmacy, East China University of Science and Technology, Shanghai, 200237 P. R. China; 3 Department of Clinical Laboratory, Linyi People’s Hospital, Xuzhou Medical University, Xuzhou, Shandong 276000 P. R. China; 4 Shanghai Jiao Tong University Affiliated Sixth People’s Hospital, Shanghai, 200233 China; 5 CAS Key Laboratory of Tissue Microenvironment and Tumor, Shanghai Institute of Nutrition and Health, University of Chinese Academy of Sciences, Chinese Academy of Sciences, Shanghai, 200031 P. R. China; 6 Institute for Stem Cell and Regeneration, Chinese Academy of Sciences, 100101 Beijing, P. R. China

**Keywords:** Cell proliferation, Hepatocytes

## Abstract

The early phase lipid accumulation is essential for liver regeneration. However, whether this acute lipid accumulation can serve as signals to direct liver regeneration rather than simply providing building blocks for cell proliferation remains unclear. Through in vivo CRISPR screening, we identify MIER1 (mesoderm induction early response 1) as a key epigenetic regulator that bridges the acute lipid accumulation and cell cycle gene expression during liver regeneration in male animals. Physiologically, liver acute lipid accumulation induces the phosphorylation of EIF2S1(eukaryotic translation initiation factor 2), which consequently attenuated *Mier1* translation. MIER1 downregulation in turn promotes cell cycle gene expression and regeneration through chromatin remodeling. Importantly, the lipids-EIF2S1-MIER1 pathway is impaired in animals with chronic liver steatosis; whereas MIER1 depletion significantly improves regeneration in these animals. Taken together, our studies identify an epigenetic mechanism by which the early phase lipid redistribution from adipose tissue to liver during regeneration impacts hepatocyte proliferation, and suggest a potential strategy to boost liver regeneration.

## Abstract (web summary)

Early lipid accumulation is thought to contribute to liver regeneration through unclear functional mechanisms. Here the authors identify an epigenetic regulator, MIER1, that bridges the acute lipid accumulation and cell cycle gene transcription during liver regeneration after surgical resection in male mice.

## Introduction

It has long been recognized that the early regenerating liver accumulates lipids temporarily, and a number of experimental observations suggest that the acute liver steatosis is indeed essential for normal liver regeneration. The hepatic lipid accumulation during liver regeneration peaks in 12–24 h after liver hepatectomy, reaching as high as three to four times in triglyceride content compared to the level before surgery, and gradually decreases to the basal level at 72 h post-surgery^1,2^. The time window in which lipid accumulation changes (12–72 h) perfectly matches with the time window of cell dividing during liver regeneration^3^. Furthermore, several studies have demonstrated impaired liver regenerative capacity when the lipid accumulation is affected. For example, liver regeneration was significantly impaired when the animals were pretreated with leptin^4^, clofibrate^5^ or propranolol^6^ that affect liver lipid accumulation; or in animals with genetic ablation of *Cav1*^7,8^ or *Lpin1*^9^, both of which resulted in dramatically reduced liver lipid accumulation. On the contrary, dietary supplementation with palmitate and carnitine enhanced toxin-induced hepatocellular proliferation^10^. However, some discrepancies also exist. In liver fatty acid binding protein (L-Fabp) knockout animals and animals with intestine-specific deletion of the microsomal triglyceride transfer protein (MTP-IKO), both of which showed partially decreased liver lipid accumulation, the liver regeneration was normal^11^. Speculation was thus raised about the “existence of a threshold of adaptive lipogenesis essential for regeneration”, which was not influenced in the L-Fabp or MTP-IKO animals^12^. However, what makes the threshold is unknown.

It is generally hypothesized that the accumulated lipids during liver regeneration serve simply as substrates for the energy supply or as biomaterials for membrane synthesis. However, several nuclear steroid hormone transcription factors, e.g. PPARα, FXR, LXR^13–16^, have been previously implicated as important during hepatectomy-induced liver regeneration. It was thus predicted that lipid derived metabolites might also influence regenerative signaling pathways via transcriptional or epigenetic mechanism, although direct support to this hypothesis has been lacking^12^. During liver regeneration, in which multiple events are highly orchestrated to support cell proliferation, it is reasonable to envisage that when the building blocks for new cells are ready, e.g., provided by lipids, signal(s) should be sent out simultaneously to coordinate the epigenetic changes for later cell proliferating; and we suspect the signal(s) should be intimately linked to the lipids itself. From a different aspect, chronic hepatic steatosis has been identified as an important risk factor that impairs liver regeneration, both observed in rodent models and human beings^17–22^. However, the basis for the differences in the influence of chronic and acute liver steatosis on regeneration is enigmatic.

With the aim to unravel unknown regulators and mechanisms in liver regeneration, we here performed a high-throughput in vivo CRISPR/Cas9 screening directly in mouse livers. We identified MIER1 (mesoderm induction early response protein 1) as a key regulator in liver regeneration. MIER1 depletion in adult hepatocytes significantly enhanced their regenerative capacity, and overexpression greatly delayed the regeneration. Interestingly, we showed that MIER1 was physiologically regulated by acute liver steatosis in liver regeneration via acute stress-induced translational control. In the meantime, high-fat diet (HFD)-fed and aging animals with chronic liver steatosis showed no response to the acute stress posed by the temporary lipid accumulation, and thus had impaired MIER1 regulation; whereas loss of MIER1 greatly improved liver regenerative capacity in these animals. Taken together, our studies present an intriguing mechanism by which acute liver steatosis in liver regeneration functions as an important signal via translationally control of MIER1 to modulate chromatin status and promote liver regeneration; and explains why the lipid accumulation and regeneration couples in healthy livers but uncouples in fatty livers.

## Results

### CRISPR in vivo screening identifies key regulators in liver regeneration

**Fig. 1:**
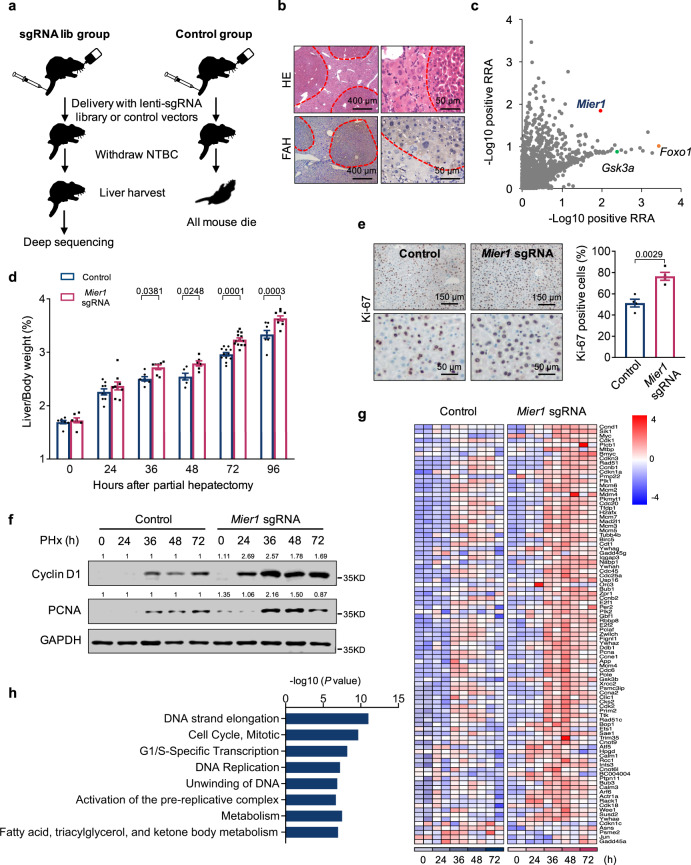
CRISPR in vivo screening identifies MIER1 as a negative regulator in liver regeneration. **a** Schematic representation for the in vivo screening procedures. **b** Liver repopulation analysis by HE staining and α-FAH immunohistochemistry. Repopulated hepatocytes are circled. **c** Identification of candidate genes using positive robust rank aggregation score from two independent screenings. The liver/body weight ratio (%) (**d**) (*n* = 7, 9, 7, 6, 12, 8 in Control group, and *n* = 7, 9, 7, 6, 13, 9 in *Mier1* sgRNA-treated group, in different time points after PHx), liver Ki-67 immunostaining at 48 h post-surgery (**e**) (*n* = 4), liver Immunoblots (Cyclin D1, PCNA, and GAPDH) (**f**), heatmap presentation of differentially liver expressed cell cycle genes (**g**), and gene enrichment analysis of liver differentially expressed genes at 24 h after surgery (**h**) in Control and *Mier1* sgRNA groups before or after surgery. Values represent means with SEM. *P* values were assessed by unpaired, two-tailed Student’s *t* test (**e**), Two-Way ANOVA with *post hoc* Šídák’s multiple-comparison tests (**d**) or two-tailed Fisher’s exact test (**h**). The experiment was repeated for three times with similar results (**f**). Quantification of western blots was performed, and the average fold changes between two groups were indicated, with ones in control group arbitrarily set as “1” (**f**). Source data are provided as a Source Data file.

To broadly assess the regulators involved in liver regeneration, we established an in vivo unbiased screening platform, taking advantage of the CRISPR/Cas9 technology and the *Fah*^*−/−*^ animals lacking the fumarylacetoacetate hydrolase, an enzyme involved in tyrosine catabolism^23^ (Fig. S1a). The lethality of the *Fah*^*−/−*^ animals can be prevented by continuous treatment with the drug nitisinone, NTBC, which blocks the accumulation of intermediary hepatotoxins^24^. To setup the in vivo screening, *Fah*^*−/−*^ animals were bred with the Cre-dependent *Cas9* knockin mice^25^, wherein Cas9 expression can be induced by the Cre-recombinase expression. The *Cas9*^LSL+/+^*Fah*^*−/−*^ animals were used for subsequent in vivo screening (Fig. S1a). We then generated lentiviral vectors harboring two main cassettes: a hepatic-specific TBG (thyroxine-binding globulin) promoter-driven *Cre-2a-Fah* cassette for hepatocyte-specific induction of Cas9 and FAH expression; a U6-driven sgRNA cassette for sgRNA pool delivery. As we were primarily interested in the regulations responsible for the dynamic change of chromatin state and gene expression during liver regeneration, we constructed a pool of sgRNAs containing in total 9094 oligos targeting 1514 genes, including epigenetic regulators (including main acetyltransferases, deacetylases, transmethylases, demethylases, and key transcription factors), kinases, and other genes of our interest, and generated a pool of lentiviral vectors (Fig. S1a and Supplementary data 1). The general idea for the screening is that upon intravenous delivery of lentiviral vectors to the *Cas9*^LSL+/+^*Fah*^*−/−*^ animals, a portion of the hepatocytes will be expressing *Fah* and sgRNA(s) targeting certain gene(s). After NTBC withdrawal, the *Fah*^*−/−*^ hepatocytes that have not received the lentiviral vectors will die shortly, whereas the hepatocytes that have successfully received the lentiviral vectors thus expressing *Fah* will survive and repopulate in vivo. We therefore can tell later in these repopulated cells the sgRNAs (hence the corresponding genes) that can likely confer liver cells a repopulation and regeneration advantage (Fig. 1a).

We first evaluated the titer needed for in vivo delivery of the lentiviral vectors. As GFP is co-expressed in the same cassette with Cas9 in the *Cas9*^LSL+/+^ animals, percentage of GFP positive cells thus offers an indication of the infection efficiency in vivo. Lentiviral delivery had a relatively low in vivo delivery efficiency. Injection of 1 × 10^9^ vectors per animal resulted in around 3% in vivo infection rate (Fig. S1b). Considering that the relatively low infection rate may help to ensure that the majority of cells receive only one sgRNA, 1 × 10^9^ vectors per animal was later adopted as the titer for screening. Lentiviruses with no *Fah* or sgRNA expression were used as control. NTBC was withdrawn 7 days after viral delivery. Whereas all mice in control group died within 40 days, all animals that received sgRNA pool vectors survived, which were sacrificed 88 days after viral delivery (Fig. S1c). In these surviving animals, efficient liver replacement from FAH-expression hepatocytes as a result of lentiviral infection was detected in liver sections (Fig. 1b).

The abundance of sgRNAs in the repopulated liver tissues as well as in the initial CRISPR vector pool were then quantified via deep sequencing. Average values for sgRNA representation were calculated and a group of sgRNAs were significantly enriched in livers treated with CRISPR library (Fig. S1d). The positive robust rank aggregation^26^ score as assessed by MAGeCK^27^ were then calculated for each gene in two independent screenings (Fig. 1c and Supplementary Data 1). Some variations between individual screenings were noticed, which may probably be due to the low in vivo infection efficiency of lentiviral vectors. However, we noticed that some genes previously identified to function in liver regeneration were screened out in our assay, indicating successful enrichment of certain negative regulators during liver regeneration (Fig. 1c). For example, studies showed that liver-specific deletion of FoxO1 can rescue the hepatic regenerative capability in Akt1- and Akt2-deficient animals;^28^ and inhibition of the glycogen synthase kinase (GSK3) led to improved liver regeneration after acetaminophen-induced acute liver failure^29^. In line with these results, both *Foxo1* and *Gsk3a* were identified as negative regulators in our screening.

### Hepatic MIER1 depletion accelerates liver regeneration after surgical resection

We next decided to follow up on *Mier1*, a gene equally significantly enriched in two independent screenings (Fig. 1c). *Mier1* encodes mesoderm induction early response protein 1, previously identified as a transcriptional repressor via recruitment of histone deacetylase (HDAC) 1 and 2^30,31^, with not much functional study in liver or liver regeneration. We first validated the functional involvement of MIER1 in liver regeneration. The *Cas9* knockin animal was adopted for specific in vivo MIER1 depletion in hepatocytes via AAV vectors (*Mier1*-sgRNA), with vectors with Cre recombinase and luciferase expression were used as control^32,33^ (Fig. S2a). Depletion of MIER1 had no obvious effects on body weight, blood aspartate aminotransferase (AST), alanine transaminase (ALT) levels, or blood liver lipids (Fig. S2b). To assess the role of MIER1 in liver regeneration, we performed partial hepatectomy, in which two-thirds of the liver was surgically removed. Consistent with the screening result, significantly improved liver regeneration was observed after MIER1 depletion, as demonstrated by significant increase in ratio of the liver/body weight (Fig. 1d), greater percentage of Ki-67^+^ cells (Fig. 1e), and enhanced liver expression of Cyclin D1 and PCNA (Fig. 1f), as compared to control group. In the meantime, we did not observe significant differences in liver weight between two groups at 12 days or 2 months after surgery, suggesting that MIER1 functions primarily in the proliferation stage during liver regeneration (Fig. S2c–e). And in general, no regional difference in liver MIER1 distribution was observed during liver regeneration (Fig. S2f). As MIER1 was previously defined as a transcription repressor, we then performed RNA sequencing (RNA-seq) analysis on liver tissues collected from both groups at different time points after surgery. Remarkably, we observed significantly and consistently increased expression of cell cycle-relevant genes after MIER1 depletion upon the onset of liver regeneration (Fig. 1g, h, S2g–i).

Human liver organoids derived from human pluripotent stem cells (hPSCs) can partly recapitulate the organogenesis events during liver regeneration^34^. Interestingly, when MIER1 was knocked down using the lenti-Cas9/CRISPR system in hPSCs, expression levels of several cell cycle markers, including Cyclin A2, Cyclin D1, PCNA were significantly enhanced in liver organoids, but not in hPSCs or foregut cells differentiation from hPSCs, after MIER1 depletion (Fig. S3a, S3b, S3c). Consistently, the size of liver organoids was clearly larger than wild-type liver organoids (Fig. S3d). Immunostaining also showed more Ki-67^+^ cells in HLOs with MIER1 depletion (Fig. S3e). In addition, no obvious difference was observed in albumin secretion or expression levels of several hepatocyte markers, indicating that MIER1 depletion does not affect liver characteristics of HLOs (Fig. S3f, S3g). These results altogether suggest a possible similar effect of MIER1 depletion in promoting human liver cell proliferation. However, as MIER1 depletion started in hPSCs, whether MIER1 depletion promoted proliferation of hepatic progenitor cells or mature hepatocytes is so far unclear. Deeper analyses to the function of MIER1 in HLO differentiation and proliferation, as well as in human liver regeneration processes, are warranted in the future before making a conclusion. Nonetheless, these data altogether suggest MIER1 as a negative regulator during liver regeneration.

### MIER1 targets cell cycle genes and regulates their expression through chromatin remodeling

**Fig. 2:**
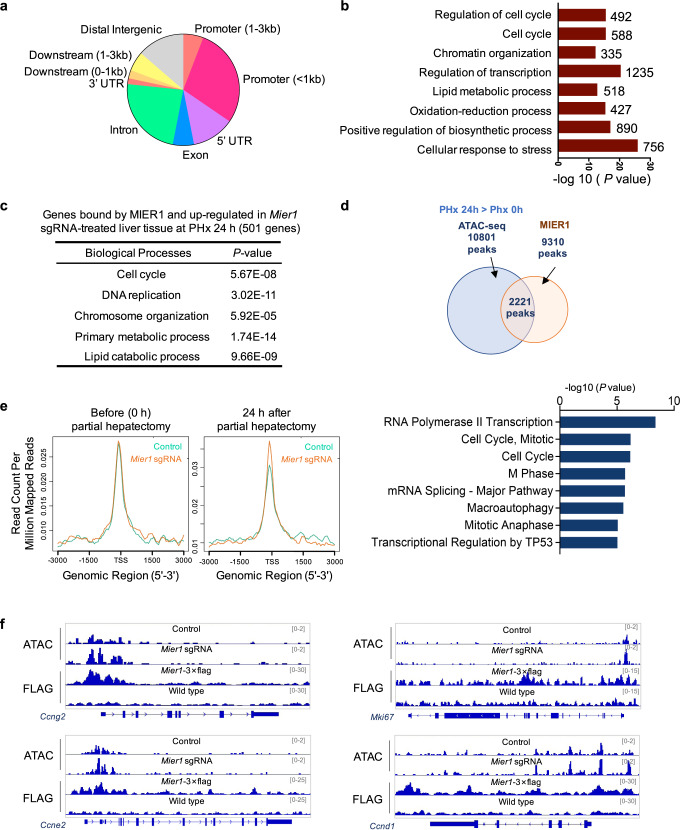
MIER1 targets and regulates cell cycle gene expression through chromatin remodeling. **a** Genomic distribution of MIER1 binding sites. **b** Gene enrichment analysis of genes bound by liver MIER1. Numbers of genes in each GO term are shown. **c** Gene enrichment analysis of genes both bound by MIER1 and up-regulated in *Mier1*-depleted liver tissue. **d** Overlapped peaks of liver MIER1 with increased ATAC-seq peaks at 24 h post-surgery as compared to ones before surgery (above); Gene enrichment analysis of genes near these overlapped peaks (below). **e** Average intensity of ATAC-seq signals of the overlapped 2221 peaks as in (**d**) surrounding the transcription start site (TSS) in Control and *Mier1* sgRNA groups at 24 h post hepatectomy. **f** Liver ATAC-seq tracks and MIER1 signals near cell cycle-relevant genes at 24 h after surgery in different groups as indicated. *P* values were assessed by two-tailed Fisher’s exact test (**b**, **c**, **e**).

MIER1 was previously reported to function in repressing gene expression through the chromatin recruitment of HDAC1 and HDAC2^30,31^. To further examine whether MIER1 directly target cell cycle genes, which showed consistent upregulation in livers after MIER1 depletion during liver regeneration, we next performed chromatin immunoprecipitation followed by sequencing (ChIP-seq) in liver tissues. Due to a lack of available MIER1 antibody for ChIP-seq analysis, exogenous liver expression of MIER1-FLAG under TBG promoter was executed using AAV vectors, and ChIP-seq was conducted using FLAG antibody. Liver tissues from animals receiving empty vectors were also collected for ChIP-seq with FLAG antibody to control for possible non-specific binding signals from FLAG antibody. We observed in total 9310 sites bound by MIER1, the majority of which were around the transcription start sites (TSS) or in introns or exons (Fig. 2a). Analysis of genes bound by MIER1 revealed significant enrichment of genes in regulation of cell cycle, chromatin organization, and transcription (Fig. 2b). Further analysis combining the results from RNA-seq revealed a significant set of genes involved in cell cycle, DNA replication and chromosome organization, that were bound by MIER1 and upregulated after MIER1 depletion in liver tissues, indicating a direct functional regulation of these genes via MIER1 (Fig. 2c and Supplementary Data 2).

We next assessed whether MIER1 depletion led to altered chromatin accessibility, especially near cell cycle related genes, by performing genome-wide ATAC-seq analysis. In total 17556 peaks were identified with ATAC-seq in quiescent livers before partial hepatectomy, in which 3661 peaks were also bound by MIER1 (Fig. S4a). In addition, consistent with the previous discovery^35^, the ATAC-seq results showed that, even not in active transcription, cell cycle genes resided in open chromatin in quiescent liver (Fig. S4a). And these genes were also bound by MIER1 (Figs. 2b, 2c, S4a). A further ATAC-seq analysis of liver tissues at 24 h after partial hepatectomy identified 10801 peaks that showed higher intensity in comparison to ones in quiescent liver, suggesting increased chromatin accessibility near these genomic loci in early liver regeneration (Fig. 2d, S4b). Of these 10801 peaks, 2221 peaks were bound by MIER1, which were close to cell cycle-relevant genes (Fig. 2d). Interestingly, although liver MIER1 depletion did not change the chromatin accessibility of these genomic loci in quiescent livers, MIER1 depletion resulted in a significant increase in chromatin accessibility at 24 h after partial hepatectomy (Fig. 2e, f). These results were consistent with the RNAseq analysis, suggesting that liver MIER1 depletion increased the chromatin accessibility and transcription of cell cycle-relevant genes during liver regeneration, but not in quiescent livers.

In addition, signals of MIER1 displayed significant overlap with hepatic genomic binding signals from H3K27ac (GSM2040959), a histone marker of active promoter and enhancer (Fig. S4c). And MIER1 depletion resulted in increased H3K27ac signal at 24 h after hepatectomy, especially near some cell cycle genes (Fig. S4d, S4e). Although we observed interaction between MIER1 and HDAC1 and HDAC2 (Fig. S4f), consistent with the previous finding, the genomic recruitment of HDAC1 or HDAC2 did not change upon liver MIER1 depletion (Fig. S4g). The chromatin regulatory mechanism by MIER1 specifically during liver regeneration warrants future investigation. Nonetheless, these above results altogether demonstrated that MIER1 targets and represses the expression of cell cycle genes in the liver regeneration process.

### MIER1 overexpression delays liver regeneration

**Fig. 3:**
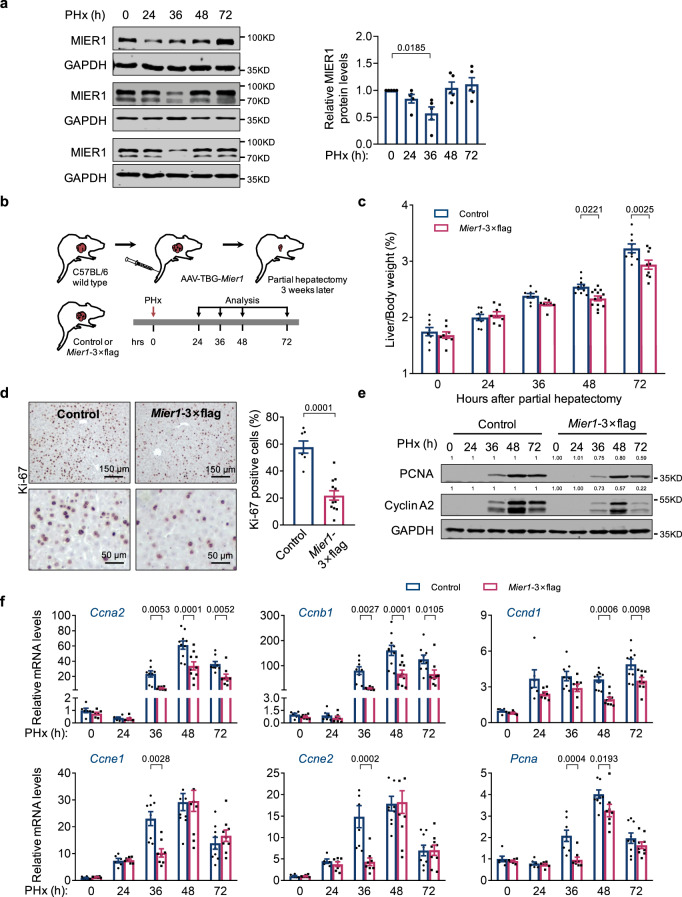
MIER1 overexpression delays liver regeneration. **a** Immunoblots (MIER1 and GAPDH) in liver tissues before or after hepatectomy. *n* = 5. **b** Schematic presentation of study with MIER1 overexpression. The liver/body weight ratio (%) (**c**) (*n* = 8, 8, 8, 10, 9 in Control group, and *n* = 8, 8, 8, 13, 9 in *Mier1-*3×flag group, in different time points after PHx), liver Ki-67 immunostaining (**d**) (*n* = 7 in Control group, and *n* = 12 in *Mier1-*3×flag group), liver immunoblots (Cyclin A2, PCNA, and GAPDH) (**e**), and mRNA levels of liver cell cycle genes (**f**) (*n* = 6, 6, 8, 10, 9 in Control group, and *n* = 7, 7, 8, 10, 9 in *Mier1-*3×flag group, in different time points after PHx) in Control and *Mier1-*3×flag groups before or after surgery. Values represent means with SEM. *P* values were assessed by unpaired, two-tailed Student’s *t* test (**d**), One-Way ANOVA with Dunnett’s multiple comparisons test (**a**), or Two-Way ANOVA with post hoc Šídák’s multiple-comparison tests (**c**, **f**). The experiment was repeated for three times with similar results (**e**). Quantification of western blots was performed, and the average fold changes between two groups were indicated, with ones in control group arbitrarily set as “1” (**e**). Source data are provided as a Source Data file.

We further asked about the physiological function of MIER1 during liver regeneration. Interestingly, we noticed a significant decrease of MIER1 protein expression shortly (24–36 h) after surgery, which largely recovered around 48 h post-surgery (Fig. 3a, S5a). Considering the physiologically increased expression of cell cycle genes during early liver regeneration and the repressive regulation of MIER1 to these genes, we suspect that the decreased expression of MIER1 in early regeneration stage may account for the increased expression of cell cycle genes.

To test this hypothesis, we exogenously overexpressed MIER1 specifically in vivo in adult mouse hepatocytes via AAV vectors (Fig. 3b, S5b). Remarkably, we observed significantly delayed liver regeneration upon MIER1 overexpression, as demonstrated by delayed recovery of liver/body weight ratio (Fig. 3c), decreased percentage of Ki-67^+^ hepatocytes (Fig. 3d), and significantly reduced expression of cell cycle genes (Fig. 3e, f). Taken together, we found that MIER1 depletion accelerated, and MIER1 overexpression delayed, liver regeneration. We thus suspect that MIER1 acts as an epigenetic “*brake”* during liver regeneration, which normally represses cell cycle gene expression; upon the start of liver regeneration, decreased MIER1 protein releases the “*brake”*, resulting in increased cell cycle gene expression, and cell proliferation.

### Acute hepatic steatosis regulates MIER1 expression via translational control

We next wanted to assess which signal(s) in the early phase of liver regeneration leads to MIER1 downregulation. We noticed that there was a decrease in food intake after surgery (Fig. S5c). To exclude that decreased food intake may affect MIER1 expression non-specifically, we performed sham surgery without liver excision. The results showed that sham procedure did also lead to reduced food intake, however, had no impact on MIER1 expression nor liver cell proliferation (Fig. S5c, S5d). Long-time fasting may lead to liver steatosis. However, no significant liver steatosis was observed in sham surgery (Fig. S5e), possibly because that the reduced food intake was only transient in this situation. The above results further confirmed that the dynamic MIER1 downregulation was specific to certain regeneration signals.

We next examined the possible effects of several major events in early regeneration using primary mouse hepatocytes^12^, including the secretion of cytokines and growth factors, e.g., interleukin-6 (IL-6), tumor necrosis factor-α (TNF α), hepatocyte growth factor (HGF), epidermal growth factor (EGF), by treating cells with these cytokines (Fig. S6a); and the rapid decrease of blood glucose by incubating cells in low and high glucose medium (Fig. S6b). However, no clear difference was observed in MIER1 expression after these treatments.

**Fig. 4:**
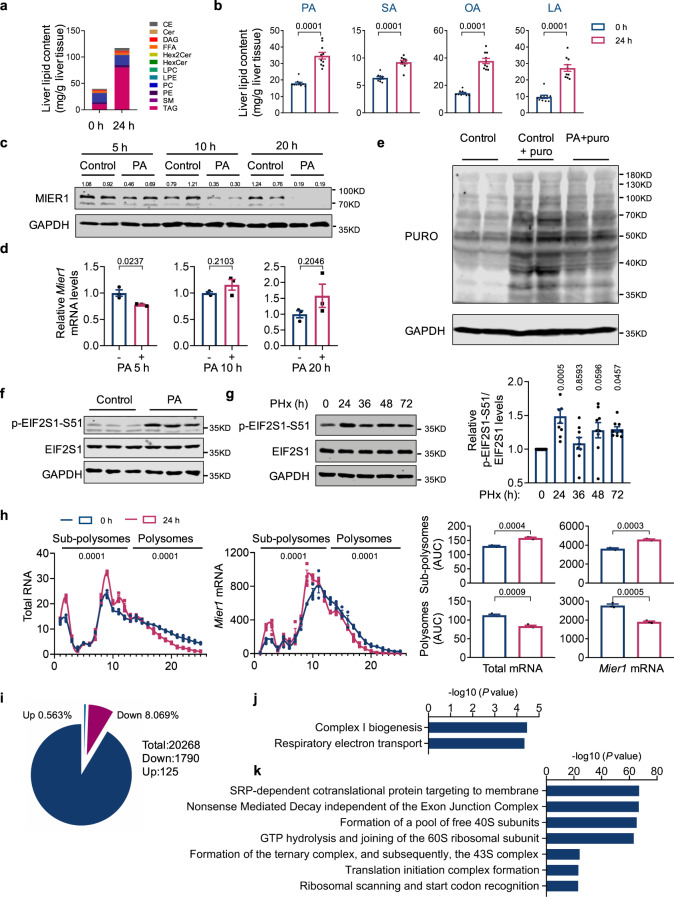
Acute hepatic lipid accumulation regulates MIER1 expression via acute stress-induced translational control. Quantification of different lipid species (**a**) and major fatty acids in lipid composition (**b**) in liver tissues from NCD animals at 0 h and 24 h after PHx. *n* = 10. Immunoblots (MIER1 and GAPDH) (**c**) and relative *Mier1* mRNA level (**d**) in primary hepatocytes after treatment with palmitic acid (PA, 0.5 mM) for indicated times. *n* = 3. Protein synthesis analysis (**e**) and immunoblots of EIF2S1 phosphorylation (**f**) in primary hepatocytes treated with or without PA for 10 h. Images show detection of puromycylated nascent proteins (**e**). **g** Immunoblots of liver EIF2S1 phosphorylation before or after hepatectomy. *P* values are between each group with group “0 h”. *n* = 3. **h** Polysome profiles and qRT-PCR analysis of distributions of indicated transcripts in liver tissues before or 24 h after hepatectomy. *n* = 3. **i** Percentage of transcripts with increased or decreased translational activity as assessed by polysome separation followed by RNA-seq analysis in liver tissues at 24 h after hepatectomy, as compared to tissues before surgery. Gene enrichment analysis of genes with decreased (**j**) or increased (**k**) translational activity as identified in (**i**). Values represent means with SEM. *P* values were assessed by unpaired, two-tailed Student’s *t* test (**b**, **d**, **h**), One-Way ANOVA with Dunnett’s multiple comparisons test (**g**) or two-tailed Fisher’s exact test (**j**, **k**). The experiment was repeated twice with similar results (**c**). The average values of the quantification of western blots were indicated (**c**). Source data are provided as a Source Data file.

Acute steatosis was previously observed in liver early regeneration stage^1,2^. We then characterized the content of acute steatosis via lipidomic analysis of liver tissues collected before and 24 h after surgery (Supplementary Data 3). A remarkable increase in the level of triacylglycerols (TAG), diacylglycerol (DAG), cholesteryl ester (CE), phosphatidylethanolamine (PE), among others, was observed in liver at the early regeneration stage (Fig. 4a, S6c, S6d, S6e). It is well received that the systematic catabolism of adipose tissue, and consequently the release of free fatty acids (FFA) to circulation and uptake by liver tissue, explains the majority of accumulated hepatic lipids during the early phase of liver regeneration^2,11,36–38^. We thus next calculated the major fatty acids that comprise the increased lipids. The results showed that the palmitic acid (PA, 16:0), stearic acid (SA, 18:0), oleic acid (OA, 18:1), and linoleic acid (LA, 18:2) are the most abundant fatty acids in the lipid composition (Fig. 4b, Supplementary Data 4).

We thus treated the primary hepatocytes with these FFAs. Interestingly, we observed rapid, time and dose-dependent decrease of MIER1 expression when cells were exposed to FFAs, especially SA and PA (Fig. 4c, S6f). No significant change at the *Mier1* mRNA level was observed after PA treatment for 10 or 20 h, suggesting that the decreased MIER1 expression after FFA treatment was primarily transcription-independent (Fig. 4d). We next assessed whether the MIER1 reduction was due to enhanced protein degradation. We thus co-treated the cells with PA and individual inhibitors to assess any possible protein degradation pathways that may account for the decreased MIER1 protein level, including MG132 (proteasome inhibitor), Calpeptin (cysteine protease inhibitor), Z-VAD (pan-Caspase inhibitor) and 3-MA (autophagy inhibitor) (Fig. S7a). However, no significant difference was observed (Fig. S7a). We thus suspect that PA treatment caused affected protein translation. Indeed, clearly decreased protein synthesis rate was noticed after PA treatment using the puromycin labeling assay^39^ (Fig. 4e). Induction of EIF2S1 phosphorylation, which limits ternary complex recycling, and inhibition of mTORC1 are two major mechanisms of translational suppression^40^. Interestingly, we observed a significant increase in phosphorylation at the S51 site in EIF2S1 after PA treatment in primary hepatocytes (Fig. 4f). More importantly, the signal of p-EIF2S1-S51 showed a transient increase at 24 h post-surgery in vivo during liver regeneration, consistent with the peak of acute steatosis (at ~24 h post-surgery) (Fig. 4g). We further treated the animals with an ISR inhibitor, ISRIB, which blunts the ISR by allosterically antagonizing the inhibitory effect of p-eIF2 on eIF2B^41^, during liver regeneration. Indeed, ISRIB treatment led to significantly inhibited MIER1 downregulation during normal liver regeneration (Fig. S7b), further suggesting a causative association between EIF2S1 phosphorylation and MIER1 downregulation. In addition, mTORC1 signaling pathway showed a consistent activation after surgery as demonstrated by S240/S242 phosphorylation of RPS6 (Fig. S7c). We thus later focused on the EIF2S1 pathway.

To further confirm that MIER1 downregulation was due to attenuated translation, we performed sucrose density gradient centrifugation to separate translated (associated with polysome fraction), and non-translated (associated with sub-polysome fraction) transcripts in extracts from mouse livers before (0 h) and 24 h post-surgery. Indeed, compared to liver tissues before surgery, extracts from tissues 24 h post-surgery had significantly fewer transcripts associated with polysomes (25.615% reduction), in which *Mier1* transcripts with polysomes were also significantly reduced (32.087% reduction), confirming a reduction in MIER1 protein synthesis activity at 24 h post-surgery (Fig. 4h, S8a). Consistent with increased p-EIF2S1-S51, a significant increase in polysome-associated *Atf4* transcripts was observed in liver tissues from 24 h post-surgery, suggesting the translational induction of ATF4, which is known to be upregulated when ISR is induced (Fig. S8b). Further RNA-seq and qRT-PCR analyses in transcripts associated with polysomes and sub-polysomes before and 24 h post-surgery showed a significant number of transcripts (8.069%) with decreased translational activity at 24 h post-surgery, which were functionally enriched in mitochondrial complex I biogenesis and respiratory electron transport (Figs. 4i, 4j, S8c, Supplementary Data 5). Meanwhile, a portion of gene transcripts (0.56%) also showed increased translational activity, which were mostly functionally associated with ribosome biogenesis and protein translation initiation (Figs. 4i, 4k, S8d, Supplementary Data 5). Collectively, these results demonstrated a significant and dynamic change of protein translational activity at the early phase of liver regeneration, and identified that *Mier1* was among the transcripts with significantly reduced protein synthesis activity, contributing to MIER1 reduction at around 24–36 h post-surgery.

**Fig. 5:**
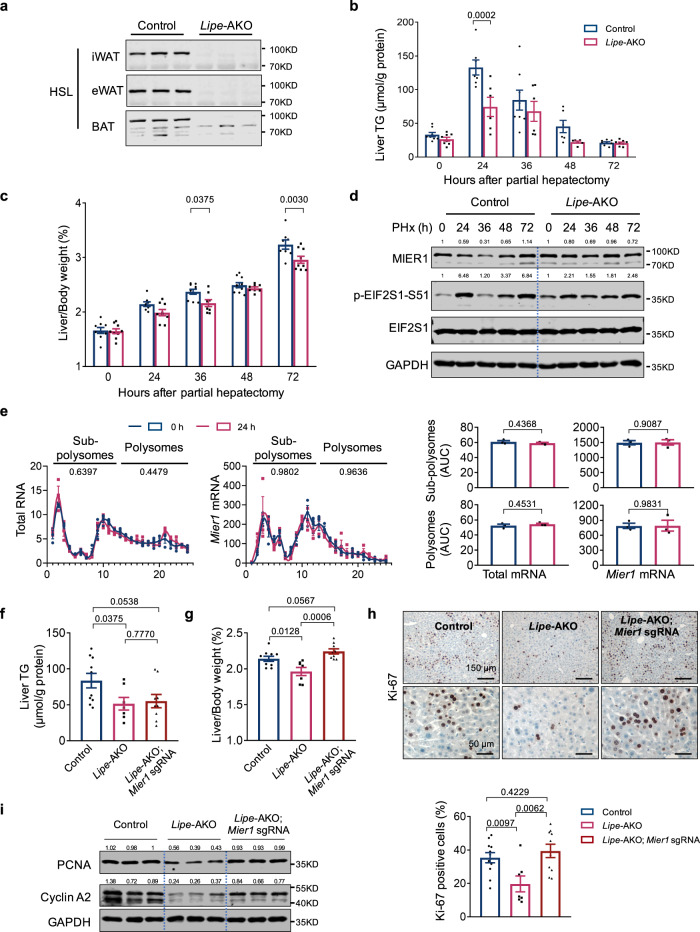
Decreased lipid accumulation due to adipose lipolysis dysfunction leads to compromised MIER1 regulation and impaired liver regeneration. **a** Immunoblot (HSL) in Control and *Lipe-*AKO adipose tissues. The liver triglycerides (TG) content (**b**) (*n* = 8, 7, 8, 6, 8 in Control group, and *n* = 8, 6, 6, 6, 8 in *Lipe-*AKO group, at different time points after PHx), liver/body weight ratio (%) (**c**) (*n* = 9, 8, 10, 10, 8 in Control group, and *n* = 9, 9, 8, 8, 9 in *Lipe-*AKO group, at different time points after PHx), immunoblots of liver MIER1 and EIF2S1 phosphorylation (**d**) in Control or *Lipe*-AKO animals before or after surgery. **e** Polysome profiles and qRT-PCR analysis of distributions of indicated transcripts in liver tissues collected before or 24 h after hepatectomy from *Lipe*-AKO animals. *n* = 3. The liver TG content (**f**) (Control, *n* = 10; *Lipe*-AKO, *n* = 7; *Lipe*-AKO; *Mier1* sgRNA, *n* = 9), liver/body weight ratio (%) (**g**) (Control, *n* = 10; *Lipe*-AKO, *n* = 7; *Lipe*-AKO; *Mier1* sgRNA, *n* = 9), liver Ki-67 immunostaining (**h**) (Control, *n* = 10; *Lipe*-AKO, *n* = 7; *Lipe*-AKO; *Mier1* sgRNA, *n* = 9), and liver immunoblots (PCNA, Cyclin A2 and GAPDH) (**i**) in Control, *Lipe*-AKO or *Lipe*-AKO; *Mier1* sgRNA animals at 36 h after hepatectomy. Values represent means with SEM. *P* values were assessed by unpaired, two-tailed Student’s *t* test (**e**), Two-Way ANOVA with post hoc Šídák’s multiple-comparison tests (**b**, **c**), or One-Way ANOVA with Dunnett’s multiple comparisons test (**f**, **g**, **h**). The experiments were repeated for three times with similar results (**d**, **i**). The average values of the quantification of western blots were indicated (**d**, **i**). Source data are provided as a Source Data file.

EIF2S1 is a well-known key component of the integrated stress response (ISR)^42,43^. The transient increase in EIF2S phosphorylation suggests that there exists an acute stress at 24 h post-surgery, probably due to lipid accumulation^44,45^. We thus next wanted to further assess that it is the acute steatosis during liver regeneration that causes ISR and hence the reduction of MIER1 protein synthesis in vivo. Consistent with the previous discovery of adipose tissue catabolism in liver regeneration^2,11,36–38^, a significant reduction in weights of epididymal and inguinal white adipose tissues (eWAT and iWAT) was observed after the onset of liver regeneration, whereas liver MIER1 depletion or overexpression had no effect on the catabolism process (Fig. S9a, S9b). In order to modulate early phase liver steatosis during liver regeneration, we partially blocked the adipose catabolism via generating the animals with *Lipe* specifically knocked out in adipose tissues (*Lipe*-AKO) (Fig. 5a). *Lipe* encodes hormone-sensitive lipase (HSL), one of the major lipases in lipid catabolism, which catalyzes the hydrolysis of triacylglycerols (TAGs), diacylglycerols (DAGs), monoacylglycerols (MAGs), with a higher preference of DAGs over TAGs and MAGs^46,47^. A significant increase of HSL phosphorylation was noticed in adipose tissues at the early onset of liver regeneration, suggesting the involvement of HSL in lipid redistribution induced by liver regeneration (Fig. S9c, S9d).

We then performed partial hepatectomy in the *Lipe*-AKO as well as the *Lipe* ^loxp/loxp^ animals as controls. Consistent with the function of HSL in adipose catabolism, the hepatic lipid accumulation in *Lipe*-AKO animals was remarkably reduced, with only half the content in liver triglycerides, when compared to control livers at 24 h post-surgery (Fig. 5b), together with compromised reduction in adipose tissue weights and reduced serum free fatty acids (FFA) during liver regeneration (Fig. S9e, S9f). Moreover, loss of HSL in adipose tissues significantly delayed the progress of liver regeneration, as revealed by a significant reduction in the ratio of liver/body weight in *Lipe*-AKO animals at 36 h and 72 h (Fig. 5c). Interestingly, both the phosphorylation of EIF2S1-S51 and MIER1 protein level remained largely constant in *Lipe*-AKO animals, in comparison to the dynamic alteration in wild-type animals during liver regeneration (Fig. 5d), indicating of the disappearance of liver ISR and MIER1 downregulation, possibly due to less lipid accumulation. Consistently, the ribosome fractionation analysis showed minimal change in protein synthesis or distribution of the *Mier1* transcripts at different time points (0 h vs. 24 h) after surgery in *Lipe*-AKO animals (Fig. 5e). To confirm that the compromised reduction of MIER1 observed in *Lipe*-AKO animals at the early phase actually accounts for affected liver regeneration, we further depleted hepatic MIER1 in *Lipe*-AKO animals. MIER1 depletion did not affect the liver lipid accumulation (Fig. 5f), however, successfully rescued the liver regenerative capacity (Fig. 5g–i), and cell cycle-relevant gene expression (Fig. S9g) in *Lipe*-AKO animals, pointing out a critical role of affected MIER1 reduction in compromised liver regeneration in *Lipe*-AKO animals.

**Fig. 6:**
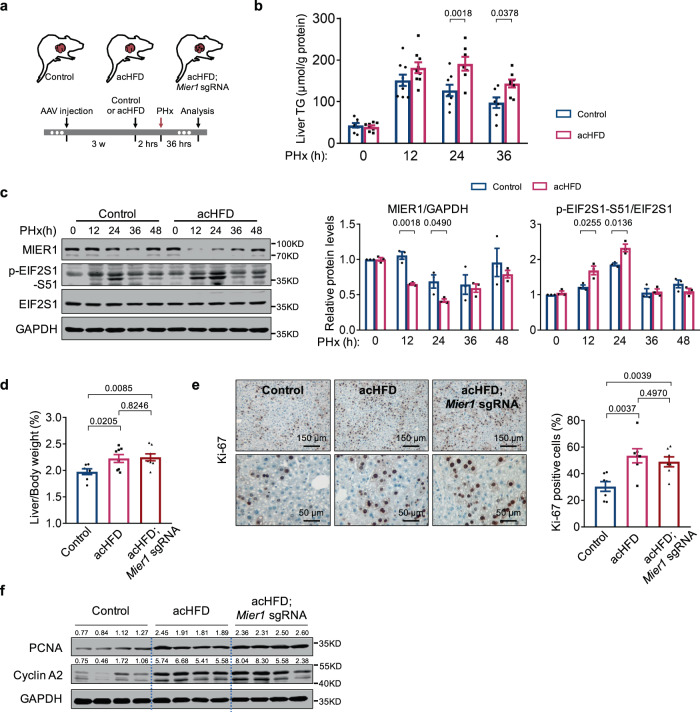
Acute HFD treatment improves liver regeneration. **a** Schematic presentation of acute HFD (acHFD) treatment. The liver TG content (**b**) (*n* = 8, 9, 8, 7 in Control group, and *n* = 8, 9, 7, 8 in acHFD group, in different time points after PHx), and liver immunoblots of MIER1 and EIF2S1 phosphorylation (**c**) (*n* = 3) in Control or acHFD animals before or after hepatectomy. The liver/body weight ratio (%) (**d**) (Control, *n* = 7; acHFD, *n* = 8; acHFD; *Mier1* sgRNA, *n* = 8), liver Ki-67 staining (**e**) (*n* = 7), and liver immunoblots (PCNA, Cyclin A2 and GAPDH) (**f**) in Control, acHFD, and acHFD; *Mier1* sgRNA animals at 36 h after hepatectomy. Values represent means with SEM. *P* values were assessed by Two-way ANOVA with post hoc Šídák’s multiple-comparison tests (**b**, **c**), or One-Way ANOVA with Dunnett’s multiple comparisons test (**d**, **e**). The experiment was repeated for four times with similar results (**f**). The average values of the quantification of western blots were indicated (**f**). Source data are provided as a Source Data file.

We next asked whether an earlier boost in liver lipid accumulation can cause earlier MIER1 reduction and accelerated liver regeneration. We thus pretreated the animals with an acute HFD (acHFD) shortly before hepatectomy (Fig. 6a). Compared with NCD animals, acHFD animals displayed increased accumulation of liver lipids (Fig. 6b), and accordingly, a significant enhancement of the p-EIF2S1 signal and the reduction in MIER1 level at as early as 12 h after surgery (Fig. 6c, S10a). Furthermore, the regenerative capacity was increased in acHFD-treated animals, in comparison to control animals (Fig. 6d–f). In addition, liver MIER1 depletion did not further increase the regenerative capacity in animals with acHFD treatment, suggesting a primary function of MIER1 depletion in the acHFD-promoted liver regeneration (Fig. 6d–f). Compared to NCD, more sugar is also added in HFD. To further exclude that sugar may also pose an effect to MIER1 regulation, we pre-fed animals with extra glucose and fructose before surgery. The results showed that extra sugar did not change the MIER1 regulation, cell proliferation, nor liver lipid accumulation during regeneration (Fig. S10b, S10c). Taken together, these results demonstrated that at the early phase of liver regeneration, the acute liver lipid accumulation, mostly due to catabolism in adipose tissues, caused acute stress to hepatocytes, which induced activation of the EIF2S signaling pathway, leading to a reduction in *Mier1* translation activity and MIER1 level.

### Animals with chronic liver steatosis show dysregulated MIER1 regulation and hepatic MIER1 depletion leads to improved regeneration in these animals

Our previous studies suggest the function of the early phase acute steatosis in promoting liver regeneration via MIER1 regulation. However, liver tissues with chronic steatosis were known to have compromised regenerative capacity^17–22^. We were therefore curious to see whether the reduced liver regenerative capacity in these conditions associates with MIER1 regulation.

**Fig. 7:**
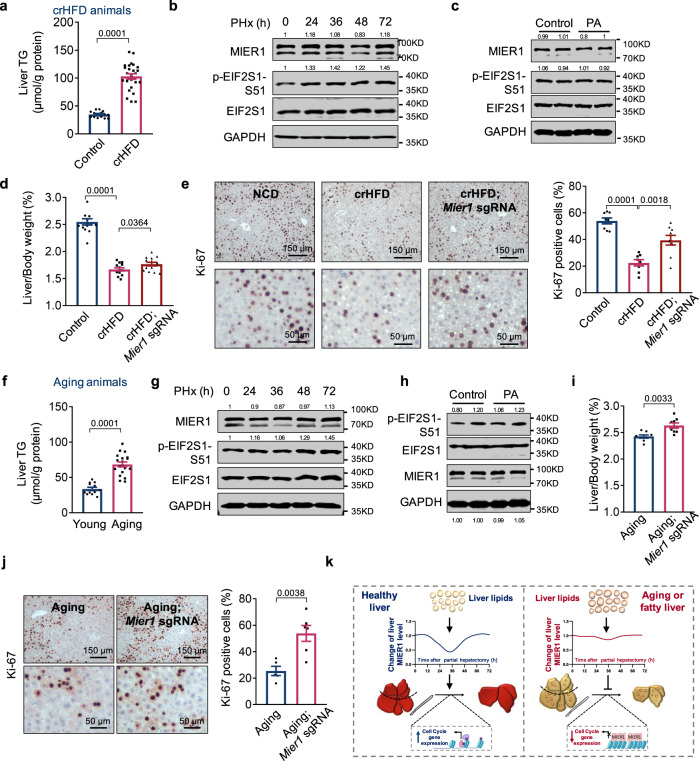
Animals with chronic liver steatosis show dysregulated MIER1 expression and improved liver regeneration upon MIER1 depletion. **a** Liver TG analysis in animals fed with NCD or HFD for 8 weeks (crHFD) (Control, *n* = 15; crHFD, *n* = 25). **b** Immunoblots of liver MIER1 and EIF2S1 phosphorylation in crHFD animals before or after hepatectomy. **c** Immunoblots of MIER1 and EIF2S1 phosphorylation in primary hepatocytes from crHFD animals after PA (0.5 mM) treatment for 10 h. The liver/body weight ratio (%) (**d**) (*n* = 14), and liver Ki-67 immunostaining (**e**) (Control, *n* = 8; crHFD, *n* = 8; crHFD; *Mier1* sgRNA, *n* = 9) in NCD, crHFD, and crHFD; *Mier1* sgRNA animals at 48 h after hepatectomy. **f** Liver TG analysis in young (~8 weeks’ old) and aging (~12 months’ old) animals (Young, *n* = 12; Aging, *n* = 19). **g** Immunoblots of liver MIER1 and EIF2S1 phosphorylation in aging animals before or after hepatectomy. **h** Immunoblots of MIER1 and EIF2S1 phosphorylation in primary hepatocytes from aging animals after PA (0.5 mM) treatment for 10 h. The liver/body weight ratio (%) (**i**) (*n* = 8), and liver Ki-67 immunostaining (**j**) (Young, *n* = 5; Aging, *n* = 6) in aging, and aging; *Mier1* sgRNA animals at 48 h after hepatectomy. **k** The mechanistic model illustrating the function of acute liver steatosis in promoting liver regeneration via MIER1. In healthy liver, after the onset of liver regeneration, the liver transiently accumulates lipids, which leads to attenuated *Mier1* translation. MIER1 downregulation in turn promotes cell cycle gene expression and cell proliferating; Whereas in aging or fatty liver, the signaling function of liver lipids is impaired and the MIER1 regulation is compromised, resulting in repressed cell cycle gene expression and liver regeneration. Values represent means with SEM. *P* values were assessed by unpaired, two-tailed Student’s *t* test (**a**, **f**, **i**, **j**) or One-Way ANOVA with Dunnett’s multiple comparisons test (**d**, **e**). The experiments were repeated twice with similar results (**b**, **c**, **g**, **h**). The average values of the quantification of western blots were indicated (**b**, **c**, **g**, **h**). Source data are provided as a Source Data file.

We first examined the change of MIER1 during liver regeneration in animals fed with HFD for 8 weeks (crHFD), which led to more than 2-fold accumulation of liver triglycerides compared to ones in animals under NCD (Fig. 7a). Interestingly, different from NCD animals, the MIER1 and p-EIF2S1-S51 levels were largely constant during liver regeneration in crHFD animals (Fig. 7b). Consistently, PA treatment to primary hepatocytes derived from crHFD animals did not cause significant reduction in MIER1 protein, nor increase in p-EIF2S1-S51 signal (Fig. 7c), or reduction in translational activity (Fig. S11a), altogether demonstrating a compromised MIER1 regulation during liver regeneration in crHFD animals. We further studied whether ablation of MIER1 in liver in crHFD animals can rescue the liver regenerative capacity. Consistent with previous discoveries, crHFD animals had compromised liver regeneration, as demonstrated by decreased ratio of liver/body weight (Fig. 7d), less Ki-67^+^ hepatocytes (Fig. 7e), and reduced expression of cell cycle proteins and genes, in comparison with NCD animals (Fig. S11c, d). And interestingly, in all aspects mentioned above, we noticed significant rescue of liver regenerative capacity after MIER1 depletion in crHFD animals (Figs. 7d, 7e, S11b–d).

Aging livers also displayed phenotypes as chronic steatosis (Fig. 7f), we thus next went on and examined the liver regeneration in aging animals. Similarly as seen in crHFD animals, both MIER1 protein and p-EIF2S1-S51 signal were constant during liver regeneration in aging animals (Fig. 7g). In addition, PA treatment to primary hepatocytes from aging livers did not cause significant alteration in MIER1 expression or p-EIF2S1-S51 (Fig. 7h), or protein synthesis activity (Fig. S11g). Further depletion of MIER1 in aging liver also significantly enhanced liver regeneration (Figs. 7i, 7j, S11h–j). In short conclusion, our results demonstrated that both crHFD and aging liver became insensitive to the acute lipid accumulation and had impaired MIER1 regulation during liver regeneration; whereas MIER1 depletion resulted in significantly enhanced liver regeneration in these animals.

## Discussion

It has long been recognized that the early regenerating liver accumulates lipids transiently, which functionally supports liver regeneration. However, it remains unclear whether acute liver steatosis can serve as a signal to promote liver regeneration rather than simply providing cell building blocks to sustain cell proliferation. Here in our study, through in vivo high-throughput CRISPR screenings, we have identified MIER1 as an important epigenetic regulator in liver regeneration that bridges the acute lipid accumulation and chromatin remodeling and cell proliferating in liver regeneration. After the onset of liver regeneration, acute liver steatosis (peaks at 12–24 h post-surgery) sends signals through lipids-induced acute stress, resulting in activated EIF2S1 pathway (at 24 h post-surgery), and consequently decreased translation of the *Mier1* mRNA (at 24 h post-surgery), leading to reduced MIER1 level (around 24–36 h post-surgery); reduced MIER1 in turn increased cell cycle gene expression and cell proliferating (peaks at 40–48 h post-surgery) (Fig. 7k). Overall, our study revealed a simple but effective and smart signaling manner, adopted by acute liver steatosis, to inform for chromatin remodeling via the acute stress induced alternation in the EIF2S-MIER1 signaling pathway during liver regeneration.

Importantly, in our study, we found that in liver tissues with chronic steatosis, e.g. from aging or crHFD animals, hepatocytes did not respond to lipid accumulation induced acute stress, thus had compromised MIER1 regulation; whereas hepatic MIER1 depletion significantly recovered the liver regenerative capacity in these animals. We did not notice significant difference in the MIER1 or the p-EIF2S1 levels between animals on NCD or on crHFD for 8 weeks (Fig. S11e, S11f). However, the lipidomic analyses demonstrated remarkable differences in lipid content between chronic steatosis after 8 weeks’ HFD and acute steatosis in normal liver regeneration. For example, lipids in chronic steatosis contain clearly more cholesteryl ester (CE), diacylglycerol (DAG), but less phosphatidylethanolamine (PE), phosphatidylcholine (PC) and lyso-PE (LPE) (Fig. S12, Supplementary Data 3). In addition, the relative changes in lipid levels between 0 and 24 h after surgery in crHFD animals are considerably less than ones in NCD animals (Fig. S12, Supplementary Data 3, 4). We therefore suspect that liver tissues with chronic steatosis have already been well-adapted with high liver lipid content, thus become insensitive to the additional stress posed by acute liver steatosis in early regeneration, although further investigation is needed to depict the detailed molecular mechanism underlying this phenomenon.

Regarding the direct regulator that controls the dynamic change in translational activity during liver regeneration, our initial observation suggests the EIF2S1 signaling pathway, which is a well-known sensor of ISR, and can be activated by lipids-induced ER stress in hepatocytes^45^. It was also previously reported that the physiological responses during liver regeneration involved a rapid increase and activation of ER-stress genes after hepatectomy. Besides EIF2S1, IRE1 α pathway was also activated, along with an increased expression of CHOP in the early regenerating liver^48–50^, suggesting the active function of the acute ER stress during liver regeneration. However, whether the acute ER stress is the direct inducer of ISR, or different mechanisms other than ISR, that regulates MIER1 synthesis during liver regeneration warrants further investigation. Besides MIER1, several other genes have also been identified that were under translational control induced by steatosis, although their functional contribution to liver regeneration remains unknown. Furthermore, how the MIER1 regulation pathway interacts with cytokines and growth factors mediated signaling pathways, and other epigenetic events during regeneration is also an intriguing question to be answered.

Before us, a couple of studies have been performed with in vivo screenings for potential regulators in liver regeneration. For example, one was published at as early as 2013^51^, with in total 631 shRNAs targeting genes that were found in focal genomic deletions of human hepatocellular carcinomas, which successfully identified MKK4 as a negative regulator in liver regeneration; another one was published in 2019^52^, focusing on 147 mutant genes identified from diseased liver samples with a pool of 882 sgRNAs, which also successfully validated several regulators in liver regeneration; and a recent study used both pooled CRISPR knockout and activation screening systems, targeting primarily 165 chromatin regulatory proteins, and identified BAZ2 as a druggable regulator in liver regeneration^53^. All these studies have beautifully demonstrated the power of high-throughput screenings in identifying regulators in liver regeneration. Differently from the above-mentioned studies, which have adopted the transposon system to incorporate shRNAs or sgRNAs to liver genome, we here used lentiviral system for in vivo sgRNA integration, and focused on over 1500 genes with over 9000 sgRNAs, presenting so far the largest library used for in vivo liver regeneration screening. Besides MIER1, several other interesting candidates also popped out in the screening that may involve in liver regeneration, although further detailed investigations to these genes are needed. In addition, with our experimental settings, we technically cannot discriminate between sgRNAs that delete a critical factor for liver regeneration from sgRNAs that randomly were not incorporated in the cells, which is a potential caveat of the screening. Nonetheless, our study offers another exciting case with significantly more targeting genes to start with, and a different approach. More importantly, our study identifies a new regulator in liver regeneration, and an exciting mechanism that reveals the intriguing signaling function of acute liver steatosis and explains how steatosis and liver regeneration couples in normal liver but uncouples in chronic fatty liver.

## Methods

### Animals

All animals were maintained and used in accordance with the guidelines of, and under approval by, the Institutional Animal Care and Use Committee of the Shanghai Institute for Nutrition and Health (ethical committee approval no. SINH-2020-DQR3). The animals presented a healthy status and male C57BL/6 J mice were used for all experiments. The light was on from 7 a.m. to 7 p.m., with the temperature kept at 21–24 °C and humidity at 40–70%. The two-thirds hepatectomy surgery was performed as previously described^54^. The left lateral lobe and the median lobe were surgically removed while mice were under anesthesia.

For in vivo CRISPR screening for regulators in liver regeneration, *Fah*^−/−^ mice, provided by Dr. Xin Wang, were bred with *Cas9*^LSL+/+^ mice to generate *Cas9*^LSL+/+^*Fah*^−/−^ animals. All *Fah*^−/−^ animals were kept on the drug NTBC (2-[2-nitro-4-{trifluoromethyl}benzoyl] cyclohexane-1,3-dione) at 19.2 mg per liter drinking water to prevent liver failure. To determine the necessary titers for lentivirus delivery, 8-week-old male *Cas9*^LSL+/+^ animals were administrated with lentiviral vectors at different titers by tail vein injection. Seven days after virus injection, animals were sacrificed and hepatocytes were dissociated for FACS analysis of GFP positive cells. GFP antibody (Invitrogen, A-21311) was applied for further increasing the GFP signal in FACS analysis.

*Cas9*^LSL+/+^ mice were used for liver-specific gene knockout^25^. Adeno-associated virus 8 (AAV8) expressing *Cre* recombinase and *sgRNA* targeting *Mier1* was administered by tail vein injection. A firefly luciferase expression cassette was also included in the AAV vector to assist in vivo evaluation of delivery efficiency. AAV vectors with *Cre* recombinase and luciferase cassettes and no sgRNA were used as control viruses. Viruses were dissolved in 250 µL phosphate-buffered saline (PBS) and were administered at a dose of 2 × 10^11^ vector genomes (vg) per mouse. For liver regeneration analysis in chronic high-fat diet-fed mice, 6-week-old *Cas9*^LSL+/+^ mice were fed with normal chow diet (NCD) (Shanghai Laboratory Animal Center, P1103F) or high-fat diet (HFD) (60% of energy from fat; Research Diets, D12492) for 8 weeks to induce hepatic steatosis. After 5 weeks on HFD, AAV8 vectors were administered by tail vein injection to deplete MIER1 in hepatocytes. For liver regeneration analysis in aging mice, about one-year-old *Cas9*^LSL+/+^ mice were administered with AAV8 vectors expression *Cre* recombinase and *Mier1* sgRNA to deplete MIER1 in liver cells. For liver regeneration analysis in acute high fat diet-fed mice, 8-week-old Cas9^LSL+/+^ mice were fed with high-fat diet (60% of energy from fat; Research Diets, D12492) 2 h before hepatectomy, with diet control mice fed with normal chow diet (NCD) (Shanghai Laboratory Animal Center, P1103F). Animals were subjected to partial hepatectomy (PHx) surgery for analysis 3 weeks after viral delivery. For liver regeneration analysis in sugar-treated animals, 8-week-old C57BL/6 J animals were fed with NCD and high sugar water containing 23.1 g/L fructose and 18.9 g/L d-glucose 2 h before hepatectomy, with control mice fed with NCD and normal water. For liver regeneration analysis in ISRIB treated mice, 8-week-old C57BL/6 J mice were injected with ISRIB (Selleck, S0706) at a dose of 5 mg/kg by intraperitoneal injection three times per day after hepatectomy until sacrificing for analysis. Animals injected with equal volume of DMSO (YEASEN, 60313ES60) were used as control. For sham surgery, animals were subjected to midline laparotomy only with no liver section.

For hepatic MIER1 overexpression, 8 weeks’ old male C57BL/6 J mice were purchased from Shanghai SLAC Laboratory Animal Co.,Ltd. Animals were administered with AAV8 vectors expressing MIER1-FLAG under liver-specific TBG promoter at a dose of 2 × 10^12^ vector genomes (vg) per mouse for 3 weeks before PHx.

*Lipe*^loxp/loxp^ mice were generated by a homologous recombination and classic embryonic stem cell targeting strategy (Shanghai Biomodel Organism Science & Technology Development Co.,Ltd). *Loxp* sequences were inserted flanking the second exon. Adipose tissue-specific *Lipe* knockout mice were generated by crossing *Lip*^loxp/loxp^ mice with Adiponectin-*Cre* mice (Jackson Laboratory). *Lipe*^loxp/loxp^ mice were then bred with *Lipe*^loxp/loxp^ mice: Cre^/+^ animals to generate *Lipe*^loxp/loxp^ mice (used as control) and *Lipe*^loxp/loxp^: Cre^/+^ mice (*Lipe*-AKO) as adipose-specific knockout animals. To further deplete MIER1 in the *Lipe*-AKO animals, *Cas9*^LSL+/+^ mice were bred with the *Lipe*-AKO mice to generate the *Lipe*-AKO; Cas9 ^LSL+/−^ animals, which were then subjected to MIER1 depletion via treatment with AAV-delivered *Mier1* sgRNA. Animals were subjected to PHx surgery for liver regeneration analysis at around 8–11 weeks’ old.

### In vivo CRISPR screening

For CRISPR library construction, we first modified the lentiCRISPR v2 vector (Addgene, #52961), by switching the U6-*sgRNA*-EFS-*Cas9*−*2a*-*Puromycin* cassette to the TBG-*Cre*-2a-*Fah*-U6-*sgRNA* cassette via PCR strategy. We then generated a customized sgRNA library using the above-modified vector. The sequences of the sgRNA pool were extracted from the GeCKO library A and B, with 6 sgRNAs targeting each gene as well as control sgRNAs. The oligo pool was then synthesized in CustomeArray (WA, USA). The resulting customized CRISPR library contains in total 9094 sgRNAs, targeting 1514 genes and 10 negative controls.

Lentiviral vectors carrying sgRNA library were produced in HEK 293 T cells. HEK 293 T cells were obtained from Cell Bank, Type Culture Collection Committee, Chinese Academy of Sciences, Shanghai. Briefly, HEK 293 T cells in each 15-cm dish were transfected with 14.7 μg pMDLG, 7.9 μg pVSVG, 5.7 μg pREV, 22.5 μg CRISPR plasmids using polyethyleneimine (Polysciences, 23966-1). Medium was changed 4~6 h after transfection. Lentiviral supernatants were then collected at 48 and 72 h post-transfection and centrifuged at 20,000 rpm at 4 °C for 2 h. Viral pellets were then re-suspended in DMEM at 4 °C overnight and titer was calculated using a PCR-based titration kit (Abm, LV900).

For in vivo CRISPR screening, 8 weeks’ old *Cas9*^LSL+/+^*Fah*^−/−^ mice were injected with 1×10^9^ lenti-sgRNA library viral vectors or control virus per mouse by tail vein injection. NTBC was withdrawn one week later after viral injection. Control animals died within 40 days, whereas animals injected with CRISPR viral vectors were sacrificed for further analysis.

Genomic DNA of whole livers from CRISPR-treated animals was extracted and the sgRNAs were amplified by two-step PCR as previously described^55^. The KOD DNA polymerase (TOYOBO, KOD-401) was used for PCR amplification. In the first step, a total of 50 μg genomic DNA (1 μg per 100 μL PCR reaction; 50 separate reactions for each sample) from liver tissues collected from each animal was used as DNA template; the PCR program used was 94 °C 5 min, 98 °C 30 s, 57 °C 30 s, 68 °C 35 s, 18 cycles. The primers were as following: 5ʹ- AATGGACTATCATATGCTTACCGTAACTTGAAAGTATTTCG-3ʹ and 5ʹ- CAAGGAGGAGAAAATGAAAGCCATACGGGAAGCAATAGCATG-3ʹ. The products (336 bp) were then applied in the second round of PCR as template, the PCR program used was 94 °C 5 min, 98 °C 30 s, 57 °C 30 s, 68 °C 35 s, 24 cycles. PCR primers used for amplification were as following: 5ʹ-Barcode+TGAAAGTATTTCGATTTCTTGGCTT-3ʹ, and 5ʹ-Barcode+CGGTGCCACTTTTTCAAGTT-3ʹ. An 8 bp barcode for multiplexing of different biological samples was added at 5ʹ of each primer. Products (142 bp) were gel-purified and quantified. In total 3.6 μg PCR products from each group were pooled together and subjected for deep sequencing (Illumina HiSeq4000 system).

For data analysis, the raw reads of sgRNA were demultiplexed using the FASTX-Toolkit and processed to contain only the unique sgRNA sequence. The sgRNA sequences were then assembled into a Burrows-Wheeler index using the Bowtie2build-index function^56^. The sgRNA information from deep sequencing was further aligned to the index using the Bowtie2 aligner. After alignment, the read count statistics for each library sequence were performed using MAGeCK^27^. Significant genes were based on the positive robust rank aggregation^26^ score identified in the MAGeCK analysis.

### Polysome fractionation and analysis

Liver tissues collected from animals were lysed in 450 μL of hypotonic buffer (5 mM Tris–HCl, 2.5 mM MgCl_2_, 1.5 mM KCl, 100 μg/mL cycloheximide, 2 mM DTT, 0.5% Triton X-100, 0.5% sodium deoxycholate, pH 7.5) by using the electric tissue grinder (Tiangen, OSE-Y40). Lysed tissues were centrifuged for 2 min at 14,000 rpm and the clear lysate supernatants were collected. The collected lysates were then loaded onto the top of the 10–50% (wt/vol) sucrose density gradient (20 mM HEPES-KOH, 100 mM KCl, 5 mM MgCl_2,_ pH 7.6) and subjected to ultracentrifugation at 36,000 rpm (SW 41 Ti rotor, Beckman) for 2 h at 4 °C. After ultracentrifugation, the fractionations at different gradients were sent to continuous recording of absorbance at 260 nm using the automatic density gradient separation system (Biocomp), and separated into 25 fractions with equal volume. Total RNA was later isolated from each fraction, and analyzed for individual gene expression by qRT-PCR or subjected to RNA-seq analysis.

### Puromycin incorporation assay

Primary hepatocytes were treated with puromycin (10 μg/mL) for 15 min, before lysed and processed for immunoblotting. Primary antibody (Millipore, MABE343, 1/1000) was used to detect puromycin incorporation, with GAPDH protein quantification applied for internal control of the total amount of protein present in each lane of the gel.

### Primary hepatocyte culture and treatment

Mouse primary hepatocytes were isolated from 8–10 weeks’ old C57BL/6 J male mice, or from C57BL/6 J male mice after HFD for 8 weeks in HFD related studies, or from about 12 months’ old C57BL/6 J male animals in aging related studies. Mice were anesthetized with pentobarbital sodium, before infused with 50 mL perfusion medium (1 × HBSS (Corning, 21-021-CVR), 190 mg/L EDTA, 6000 mg/L HEPES, pH 7.4) through the portal vein for 6 min using the peristaltic pump (Longer, LEAD-2). The liver was then perfused with the digestion medium (DMEM (Gibco 11885), 100 CDU/mL Collagenase I (Worthington, LS004196), 1 × PS, 15 mM HEPES) for another 5 min. Liver tissue was then placed into the 10-cm dish with digestion medium and tore apart with gentle shake. The suspension from the digested liver tissue was later filtered through a 70 μm cell strainer and centrifuged at 50 × *g* for 2 min to remove debris. Dead cells were further excluded by removing the floating part after centrifugation at 200 × *g* for 10 min in 36% percoll solution (Sigma, P4937). The remaining hepatocytes were re-suspended in DMEM medium containing 1 × PS, 10% fetal calf serum (Gibco, 10091148), 100 nmol/L dexamethasone (Sigma, D4902). The viability of isolated hepatocytes was further determined by trypan blue staining (Yeasen, 40207ES20). Hepatocytes were then seeded in collagen-coated plates at 6×10^5^ viable cells per well in a 6-well plate in DMEM medium as noted above. After 4–6 h incubation, the medium was replaced with DMEM containing FFA, cytokines or growth factors to examine the change in MIER1 expression level. Low glucose medium (Gibco, 11885) and high glucose medium (Gibco, 11995) were applied to assess the glucose effect to MIER1. OA (Sigma, O1008), LA (Sigma, L1376), SA (Sigma, S4751), PA (Sigma, P0500) were used for FFA treatments. IL-6 (Peprotech, 216-10-10), TNFα (Novoprotein, CF09), HGF (Peprotech, 100-39H), EGF (Peprotech, AF-100-15), Insulin (Sigma, I3536) were used for cytokines or growth factors treatments. MG-132 (MCE, HY-13259), Calpeptin (Targetmol, T6432), Z-VAD (MCE, HY-16658), 3-MA (Selleck, S2767) were used for inhibitor treatments.

### Generation of human liver organoids (HLOs) from human pluripotent stem cells (hPSCs) and functional analysis of HLOs

The HUES9 human embryonic stem cells (hESCs) used in this study was kindly provided by the Department of Stem Cell and Regenerative Biology in Harvard University and Harvard Stem Cell Institute. The use of hESCs in this study was approved by the ethics committee in Shanghai Institute of Nutrition and Health (SINH). The hESCs were grown in feeder-free adherent culture in chemically defined mTeSR1 (STEMCELL, 05850) supplemented with 1% penicillin/streptomycin (Beyotime, C0222). Culture plates were precoated with Geltrex (Gibco, A1413202). Lentiviruses carrying CRISPR were packaged using the same method as the in vivo CRISPR screening. hESCs were infected with lentivirus carrying *MIER1* sgRNA to knockout MIER1, with cells infected with control lentivirus carrying non-targeting sgRNA as control. Since the lentiviral vector carries the puromycin resistance gene, 48 h after virus infection, puromycin at a final concentration of 0.5 μg/mL was added to the culture medium to remove uninfected cell. Three days after puromycin treatment, the cells left were disassociated with accutase (Gibco, A1110501) and passaged for later differentiation and analysis.

The hESCs were then differentiated into foregut using a previously described^34^. Briefly, cells were seeded on Geltrex-coated 6 well culture plates with 3 × 10^5^ cells/well. The medium was then changed to RPMI 1640 (Gibco, 11875119) with 100 ng/mL activin A (Peprotech, 120-14E) and 50 ng/mL BMP4 (Peprotech, 120-05) at day 1, 100 ng/mL activin A and 0.2% FBS (Gibco, 16000-044) at day 2, and 100 ng/mL activin A and 2% FBS at day 3. On days 4 to 6, cells were cultured in advanced DMEM/F12 (Gibco, 12634010) with B27 (Gibco, 12587010), N2 (Gibco, 17502048), 500 ng/mL FGF4 (Peprotech, AF-100-31), 3 μmol/L CHIR99021 (Sigma, SML1046) and 1% penicillin/streptomycin. The medium was replaced every day. At day 6 the foregut cells were dissociated into single cells by accutase. Then Cells was then resuspended in Matrigel. About 100,000 cells were embedded in 50 μL Matrigel drop and expanded with 5 F medium for 4 days. 5 F medium contains advanced DMEM/F12, 1×B27, 1×N2, 5 ng/mL FGF2 (Peprotech, 100-18B), 20 ng/mL EGF (Peprotech, AF-100-15), 10 ng/mL VEGF (Peprotech, 100-20-10), 3 μM CHIR99021, 0.5 μM A83-01 (Tocris, 2939), 50 μg/mL ascorbic acid (Sigma, A5960) and 1% penicillin/streptomycin^57^. After 4 days’ treatment, the 5 F medium was switched to RA medium for another 4 days, which contains advanced DMEM/F12, B27, N2 and 2 μM retinoic acid (Sigma, R2625). At day 15, organoids were removed from Matrigel and re-embedded on the ultra-low attachment multiwall plate (Corning, 3473). During day 15–24, the media was switched to Hepatocyte Culture Medium (Lonza, 185319) supplemented with 10% Matrigel, 10 ng/mL hepatocyte growth factor (PeproTech, 100-39H), 0.1 mM dexamethasone (Sigma, D4902), 20 ng/mL Oncostatin M (Peprotech, 300-10) and 1% penicillin/streptomycin. The medium was replaced every 2 days. Cells were maintained at 37 °C in humidified air with 5% CO_2_.

To measure albumin secretion of HLOs, supernatants of HLOs from 24-well plate were collected at the day 24. Levels of human albumin were determined by the human serum albumin duoset ELISA (R&D system, DY1455) according to the manufacturer’s instructions.

For immunostainings of albumin and Ki-67 in HLOs, organoids were harvested at the day 24 and fixed with 4% paraformaldehyde for 30 min, followed by permeabilization with 0.1% Triton-X-100 for 1 h and 1% BSA blockage for 1 h. Samples were then incubated with primary antibodies against Albumin (1:100, R&D systems, MAB1455) and Ki-67 (1:300, CST, 12202 S) at 4 °C for 24 h, and the following secondary antibodies Alexa 488 donkey-anti-rabbit (1:200, Invitrogen, R37118), and Alexa 647 goat-anti-mouse (1:1000, Invitrogen, A21235) for 3 h. Nuclei were stained with DAPI (1:2000, Beyotime, C1002). Stained organoids were imaged by confocal microscope (Zeiss LSM 880). All image analysis was performed using the ImageJ-Fiji software.

### Histology and immunohistochemistry

Liver tissue samples were fixed in 4% paraformaldehyde and embedded in paraffin. For histopathological evaluation, sections were stained with hematoxylin and eosin according to standard protocols. For detection of FAH (1:200, Abcam, ab83770), MIER1 (1:100, Proteintech, 11452-1-AP), and Ki-67 (1:400, CST, 12202 S), immunostaining staining was performed via standard protocols. Antigen retrieval was performed for 20 min in Tris/EDTA buffer (10 mM Tris-HCl, 1 mM EDTA, 0.05% Tween-20, pH 9.0) at 100 °C. The HRP-conjugated secondary antibody was used for signal detection (CST, 8125 S).

### Lipid analysis in blood and liver tissues

For lipid analysis in liver tissues, lipids were extracted using a chloroform: methanol (2:1) method. In brief, liver tissue samples of around 20–30 mg were homogenized in 500 µL ice-cold PBS. From these, 100 µL extracts were used for measurement for protein concentration, and the remaining 400 µL extracts were mixed with 1.6 mL chloroform: methanol (2:1) mixture and vortexed. The suspension was then centrifuged at 2,500 rpm for 10 min at 4 °C, and lipid fractions in the lower organic phase were transferred into new 1.5 mL tubes and completely dried overnight in the chemical hood. The residue was later resuspended in 500 µL of 1% Triton X-100 in ethanol for subsequent triglyceride measurement by using triglyceride kit (Shanghai UNF, 1030280) and cholesterol kit (Shanghai UNF, 1040280), respectively. Lipid amount was normalized to protein concentration.

For lipid analysis in blood, blood serum was collected after centrifugation at 2,500 rpm for 20 min at 4 °C, and stored at −80 °C, or used immediately for triglyceride or cholesterol measurement using the above-mentioned kits. All procedures were performed on ice.

### Blood free fatty acid (FFA) analysis

For analysis of the blood FFA during liver regeneration, blood was collected at different time points after liver hepatectomy. Blood was then centrifuged at 2500 rpm for 20 min at 4 °C, and serum was collected and frozen at −80 °C. The serum FFA level was later measured by using the free fatty acid assay kit (Shanghai UNF, 2050152).

### RNA isolation and qRT-PCR

Total RNA was extracted from liver tissues by using TRIzol reagent (Thermo Fisher Scientific, 15596018) according to the manufacturer’s instructions. RNA from polysome fractionation was extracted by TRIzol LS reagent (Thermo Fisher Scientific, 10296028). Reverse transcription of isolated RNA was performed using the PrimeScript reverse transcription kit (Takara, RR047A). Quantitative real-time PCR was carried out on the Q6 System using SYBR Green supermix (Applied Biosystems, 4472908).

The sequences of primers used were as follows:

*Gapdh*-F: TGCGACTTCAACAGCAACTC

*Gapdh*-R: ATGTAGGCAATGAGGTCCAC

*Pcna*-F: TTTGAGGCACGCCTGATCC

*Pcna*-R: GGAGACGTGAGACGAGTCCAT

*Ccna2*-F: ACAGAGTGTGAAGATGCCCTGGCT

*Ccna2*-R: AGCATGTGGTGATTCAAAACTGCCA

*Ccnb1*-F: AGGCTGCTTCAGGAGACCATGT

*Ccnb1*-R: TGGCCGTTACACCGACCAGC

*Ccnd1*-F: GAAGGAGACCATTCCCTTGA

*Ccnd1*-R: GTTCACCAGAAGCAGTTCCA

*Ccne1*-F: ACGGGTGAGGTGCTGATGCCT

*Ccne1*-R: AGCAGAAGCAGCGAGGACACC

*Ccne2*-F: ATGTCAAGACGCAGCCGTTTA

*Ccne2*-R: GCTGATTCCTCCAGACAGTACA

*Mier1*-F: CTTCCACACCTTTCTCATCCC

*Mier1*-R: GGGCTCCATGTTTCAAGCTGA

*Cox8a*-F: GCTCAGGTCCACTCGAAG

*Cox8a* -R: CTTGTAGCTCTCCAGGTGTG

*Ndufb9*-F: AGGATGGAGAGCTGGGATC

*Ndufb9*-R: CCGAGGTCTGGTCACAATATG

*Ndufa7*-F: TCATGTCCTCACAAAAGGCC

*Ndufa7*-R: GCAGGGTCACAGGTATGG

*Atf4-F:* GGTTCTCCAGCGACAAGG

*Atf4-R:* GCATCGAAGTCAAACTCTTTCAG

*Rpl30*-F: GTCCATCACTACAGTGGCAAT

*Rpl30*-R: CTTTCTTGTTTACTTCTCACCAGTC

*Rpl11*-F: CCAAACACAGAATCAGCAAGG

*Rpl11*-R: GGATCAAGTTTATTTTCCAGGAAGG

*Rps15*-F: TGTACAACGGCAAGACCTTC

*Rps15*-R: CGGGTTTGTAGGTGATGGAG

*ALB*-F: GCACACTTTCTGAGAAGGAGAG

*ALB*-R: CACTTCTCTACAAAAGCTGCG

*AFP*-F: TCAGTGAGGACAAACTATTGGC

*AFP*-R: GGGTTTACTGGAGTCATTTCATG

*ASGPR*-F: GAGCAGAAATTTGTCCAGCAC

*ASGPR*-R: CCTCCAGTTCTTGAAGCCC

*A1AT*-F: GGAACCTATGATCTGAAGAGCG

*A1AT*-R: TGGTCAGCACAGCCTTATG-

*GLUT2*-F: TTTCAGTCAAGGACCACGTC

*GLUT2*-R: GAGCACTCCAGCAAAGAGG

*CYP3A4*-F: TTCACCGTGACCCAAAGTAC

*CYP3A4*-R: TGAGAGCAAACCTCATGCC

*18S*-F: CGGCTACCACATCCAAGGA

*18S*-R: GCTGGAATTACCGCGGCT

### RNA-seq analysis

RNA-seq analysis was performed following standard procedures in Shanghai Majorbio Bio-pharm Technology Co.,Ltd. For RNA profiles in liver tissues at different time points after PHx from different animal groups, each sample contained pooled RNA from three biological replicas that were mixed with an equal mass of RNA to minimize variation across samples. Two samples from each group (containing RNA from six animals) were included for sequencing. For RNA-seq in polysome fractionation analysis, RNA was collected after polysome fractionation and separated to sub-polysomes RNA (a mixture of 1–12 fractions) and polysomes RNA (a mixture of 13–25 fractions), before subjected for sequencing, respectively. Three samples collected from independent animals were included for sequencing from each group. Differentially expressed genes were then identified by DEseq by using *P* ≤ 0.05 as cutoff. Gene enrichment analyses were performed with online software (http://geneontology.org/; and https://david.ncifcrf.gov/).

### ChIP-seq analysis

For the ChIP-seq analysis, chromatin was prepared from 3 biological replicates of animals in each group, and ChIP-seq assays were performed using an antibody against FLAG M2 agarose (Sigma, A2220) by Active Motif Inc. and H3K27ac (Active Motif, 38133) by DIATRE Biotechnology (China). The 75-nt sequence reads generated by Illumina sequencing (using NextSeq 500) were mapped to the genome (mm10) using the BWA algorithm with default settings. Only reads that pass Illumina’s purity filter, align with no more than 2 mismatches, and map uniquely to the genome were used in the subsequent analysis. Duplicate reads (“PCR duplicates”) are removed, unless stated otherwise. For comparative analysis, standard normalization was achieved by down-sampling the usable number of tags (the aligned reads) for each sample in a group to the level of the input control. Peaks were called using the MACS algorithms (MACS 2.1.0) with an irreproducible discovery rate (IDR) of <0.05. Peak filtering was performed by removing false ChIP-Seq peaks as defined with the ENCODE blacklist by bedtools subtract function (bedtools 2.30.0). To compare peak metrics between 2 or more samples, we used bedtools merge function to stitch overlapping intervals (peak regions) into “Merged Regions”, which are defined by the start coordinate of the most upstream Interval and the end coordinate of the most downstream Interval (= union of overlapping Intervals; “merged peaks”). In locations where only one sample has an Interval, this Interval defines the Merged Region. After defining the Intervals and Merged Regions, their genomic locations along with their proximities to gene annotations are determined.

### ChIP-qPCR analysis

ChIP-qPCR assays were carried out using the SimpleChIP Enzymatic ChIP Kit (Cell Signaling Technology, #9005) following the manufacturer’s protocol. Briefly, cells were crosslinked with 1% formaldehyde for 20 min and stopped with glycine for 5 min. The cells were later lysed with the lysis buffer and sonicated using a Bioruptor Sonicator. The samples were immunoprecipitated overnight at 4 °C with the following antibodies: HDAC1 (CST 34589 S), HDAC2 (CST 57156 S). Each reaction was incubated with protein G magnetic beads for another 2 h at 4 °C. After standard washes, elution buffer was added to all immunoprecipitation samples and the input samples. DNA from each sample was then purified for real-time PCR. Primers used in the real-time PCR were as follows:

*Ccne2*-ChIP-F: TCCGGACCAGGATGGCTAA

*Ccne2*-ChIP R: CTGCGCACCCTCAGGC

*Cdc25a*-ChIP-F: CTCTTCCTCCACGGAGTTTGTT

*Cdc25a*-ChIP-R: GGACCGGCGAGCGTAGG

*Ccpg1*-ChIP-F: CCTGGCAGGTGTTAGGCG

*Ccpg1*-ChIP-R: CACGCGGCATCTCAGCATA

### ATAC-seq analysis

Nuclei were extracted from frozen liver tissue as previously described^58^. Briefly, 30 mg frozen liver tissue from 3 different samples was added into a pre-chilled 1 mL Dounce with 1 mL cold homogenization buffer, then subjected to dounce with loose pestle for 10 strokes and dounce with tight pestle for 25 strokes. After centrifugation to remove larger cellular debris from homogenized liver fluid, the supernatant containing nuclei was further purified by the gradient density centrifugation. About 50,000 purified nuclei were used to generate the ATAC-seq library using the TruePrep DNA Library Prep Kit V2 for Illumina (Vazyme, TD501), which was then purified using the VAHTS DNA Clean Beads (Vazyme, N411), and sequenced using 2×150 bp Illumina HiSeq.

For data analysis, pair-end reads from ATAC-seq library were trimmed by using Trim Galore (v0.6.7) and aligned to the reference mouse genome UCSC mm10 by Bowtie 2 (v2.2.5) with default parameters. After removing duplicate, multiple mapping reads, and reads mapping to the mitochondrial genome, high quality reads were picked by SAMtools (v1.7) view function with following settings “-f 1 -F 4 -F 8 -F 256 -F 2048 -q 30”. To identify reads from nucleosome-free region, extraction of fragments with the insert size of <100 bp was performed by SAMtools and awk. ATAC-seq peaks were called by using HOMER (v4.11) findPeaks function with following parameters “-style dnase -minDist 200 -size 100”. Peak regions overlapping with mm10 ENCODE exclusion list regions were discarded. For visualization purposes, deepTools (v3.5.1) was used to normalize ATAC-seq read counts to Counts Per Million value with following parameters “--normalizeUsing CPM -bs 50”. In order to identify regions with more accessibility in different group, peak files from both 24 h and 0 h after partial hepatectomy group were merged into one master peak file. Tag count at each merged region were extracted and compared between two groups. Average profiles of regions of interest in each group were performed by using ngs.plot (v2.61) with following parameters “-L 3000 -FL 100 -MW 4 -SE 0”.

### Lipidomic analysis

The lipidomics analysis was performed following standard procedures in Shanghai Biotree Biomedical Technology Co., Ltd. For the lipidomics analysis, 10 biological replicates of mice liver were prepared before and 24 h after partial hepatectomy.

In the data processing stage, all peaks and metabolites were left after relative standard deviation de-noising. The missing values were then filled up by half of the minimum value, with normalization method also employed in this data analysis. The final dataset containing the information of peak number, sample name and normalized peak area was further imported to the SIMCA16.0.2 software package (Sartorius Stedim Data Analytics AB, Umea, Sweden) for multivariate analysis. Data were scaled and logarithmically transformed to minimize the impact of both noise and high variance of the variables. After these transformations, PCA (principal component analysis, PCA) was carried out to visualize the distribution and the grouping of the samples. 95% confidence interval in the PCA score plot was used as the threshold to identify potential outliers in the dataset.

The supervised orthogonal projections to latent structures-discriminate analysis (OPLS-DA) was applied to visualize group separation and find significantly changed metabolites. The sevenfold cross validation was performed to calculate the value of R2 and Q2, with R2 indicating how well the variation of a variable is explained and Q2 how well a variable could be predicted. A 200 times-permutation was further conducted to check the robustness and predictive ability of the OPLS-DA model. Afterward, the R2 and Q2 intercept values were obtained, with the intercept value of Q2 representing the robustness of the model, the risk of overfitting and the reliability of the model (the smaller the better). Furthermore, the value of variable importance in the projection (VIP) of the first principal component in OPLS-DA analysis was obtained, which summarizes the contribution of each variable to the model. The metabolites with VIP > 1 and *P* < 0.05 (Student’s *t*-test) were considered as significantly changed metabolites. CE, Cholesteryl Ester; Cer, Ceramide; DAG, Diacylglycerol; FFA, Free fatty acid; Hex2Cer, Lactosylceramide; HexCer, Hexosylceramides; LPC, lyso-PC; LPE, lyso-PE; PC, Phosphatidylcholine; PE, Phosphatidylethanolamine; SM, Sphingomyelin; TAG, Triacylglycerol.

### Western analysis and co-immunoprecipitation

Protein from cells or tissues was extracted by the RIPA buffer (Millipore, 20–188) and subjected to the regular western procedure. Protein concentrations were determined by the BCA protein assay (Thermo Fisher Scientific, 23227), and 40 μg of total protein was subjected for western analysis. A Li-COR Odyssey system (Li-COR Biosciences) and Image Studio Lite Ver 5.2 were used for fluorescent immunoblotting signal collection, and signal quantification. The following primary antibodies were used in the experiments: MIER1 (1:1000, Sigma, HPA019589), GAPDH (1:10000, ABclonal, AC002), GAPDH (1:5,000, Proteintech, 60004-1-Ig), PCNA (1:10000, Proteintech, 10205-2-AP), PCNA (1:5000, Proteintech, 60097-1-lg), Cyclin A2 (1:5000, Proteintech, 18202-1-AP), Cyclin D1 (1:1000, ABclonal, A0310), HDAC1 (1:1000, Abcam, ab7028), HDAC2 (1:1000, Abcam, ab12169), FLAG (1:1000, Sigma, F7425), puromycin (1:100000, Millipore, MABE341), EIF2S1 (1:500, ABclonal, AP0764), p-EIF2S1-S51 (1:1000, Beyotime, AF5803), RPS6 (1:1000, ABclonal, A6058), p-RPS-S240/S242 (1:500, ABclonal, AP0537), HSL (1:1000, ABclonal, A15686), p-HSL (1:1000, ABclonal, AP1242), HSP90 (1:1000, CST, 4874 S). Secondary antibodies used in the experiment were IRDye 680RD Donkey anti-Mouse (1:10000, LI-COR Biosciences, 926–68072) and IRDye 800CW Donkey anti-rabbit (1:10000, LI-COR Biosciences, 925–32213).

For endogenous co-immunoprecipitation, MIER1 was immunoprecipitated overnight at 4 °C from 1000 μg of total protein using 5 μL of Anti-MIER1 antibodies (Sigma, HPAO19589 RO8028). Immunoprecipitates were then incubated with protein A/G PLUS-Agarose beads (Santa Cruz, sc-2003) for 2 h and then washed out three times in lysis buffer before analysis by SDS-PAGE and immunoblotting. Normal rabbit IgG (Cell Signaling, 2729 S) was used as a negative control for immunoprecipitations.

### Statistics and reproducibility

The data in most figure panels reflect multiple experiments performed on different days using independent animals. Statistic differences between different groups were analyzed using the unpaired, two-tailed Student’s *t* test, Mann–Whitney test, One-Way with Dunnett’s multiple comparisons test, Two-Way ANOVA with post hoc Šídák’s multiple-comparison tests, or Two-Way ANOVA with post hoc Tukey’s multiple-comparison tests as indicated in the figure legend. Analyses were performed using GraphPad Prism software. Statistical significance was determined as *P* < 0.05, with the exact *P* values, or 0.0001 when the *P* value was equal or less than 0.0001, displayed in the figures. All data were presented as means with SEM. All western analyses were repeated for at least three times from independent samples with similar results. All micrographs were taken from at least four independent samples with similar results.

### Reporting summary

Further information on research design is available in the Nature Portfolio Reporting Summary linked to this article.

## Supplementary information

Supplementary Information

Peer Review File

Description of Additional Supplementary Files

Supplementary Data 1

Supplementary Data 2

Supplementary Data 3

Supplementary Data 4

Supplementary Data 5

Reporting Summary

## Data Availability

All data associated with this study are presented in the paper or the Supplementary Materials. Source data are also provided with this paper. The CRISPR screening data were deposited in the Sequence Read Archive under the accession NO. SRR16916496. The RNA-seq data have been deposited in the Gene Expression Omnibus under the accession NO. GSE188421, NO. GSE188768, and NO. GSE212628. The ChIP-seq data have been deposited in the Gene Expression Omnibus under the accession NO. GSE188747 and GSE188742. The ATAC-seq data have also been deposited in the Gene Expression Omnibus under the accession NO. GSE212626. The lipidomic data have been deposited in the National Omics Data Encyclopedia under the accession NO. OEP003146. The mouse genome (GRCm38/mm10) data used in this study are available in the UCSC Genome Browser [https://hgdownload.soe.ucsc.edu/goldenPath/mm10/bigZips/]. Source data are provided with this paper.

## Source data

Source data
