# Supplementary material for: Acute liver steatosis translationally controls the epigenetic regulator MIER1 to promote liver regeneration in a study with male mice
Source: Nat Commun. 2023 Mar 18;14:1521. doi: 10.1038/s41467-023-37247-9 (PMC10024732; doi:10.1038/s41467-023-37247-9)
Supplement: Supplementary file 1 — Supplementary Information [file 41467_2023_37247_MOESM1_ESM.pdf]

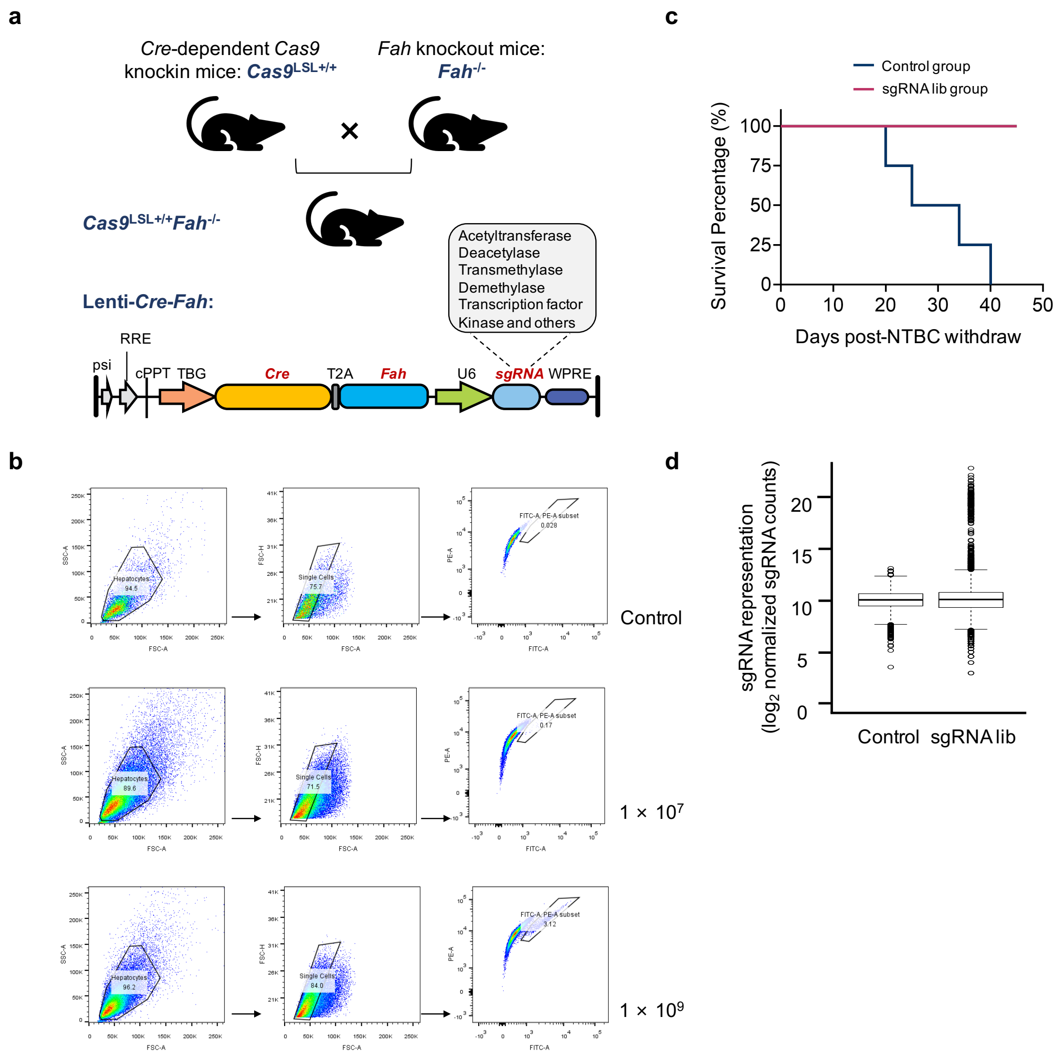

Supplementary Figure 1 CRISPR *in vivo* screening identifies key regulators in liver regeneration. (**a**) Schematic representation for the animals and the CRISPR library used in the *in vivo* screening. (**b**) FACS analysis for GFP positive hepatocytes after intravenous lentiviral delivery using different titers as indicated. Hepatocytes were disassociated for analysis 7 days after lentiviral delivery. (**c**) Survival rate analysis of the *Cas9*$^{LSL+/+}$*Fah*$^{-/-}$ animal after receiving lenti-CRISPR library expression *Fah*, *Cre* and *sgRNAs* (SgRNA lib group) or empty lentiviral vectors (Control group). (**d**) Box plot showing the distribution of sgRNA frequencies in liver tissue collected from lenti-CRISPR library-treated animals (sgRNA lib) or the CRISPR library plasmid pool (Control). n = 9094. In each boxplot, the band indicates the median, the box indicates the first and third quartiles, the whiskers indicate ± 1.5 × interquartile range.

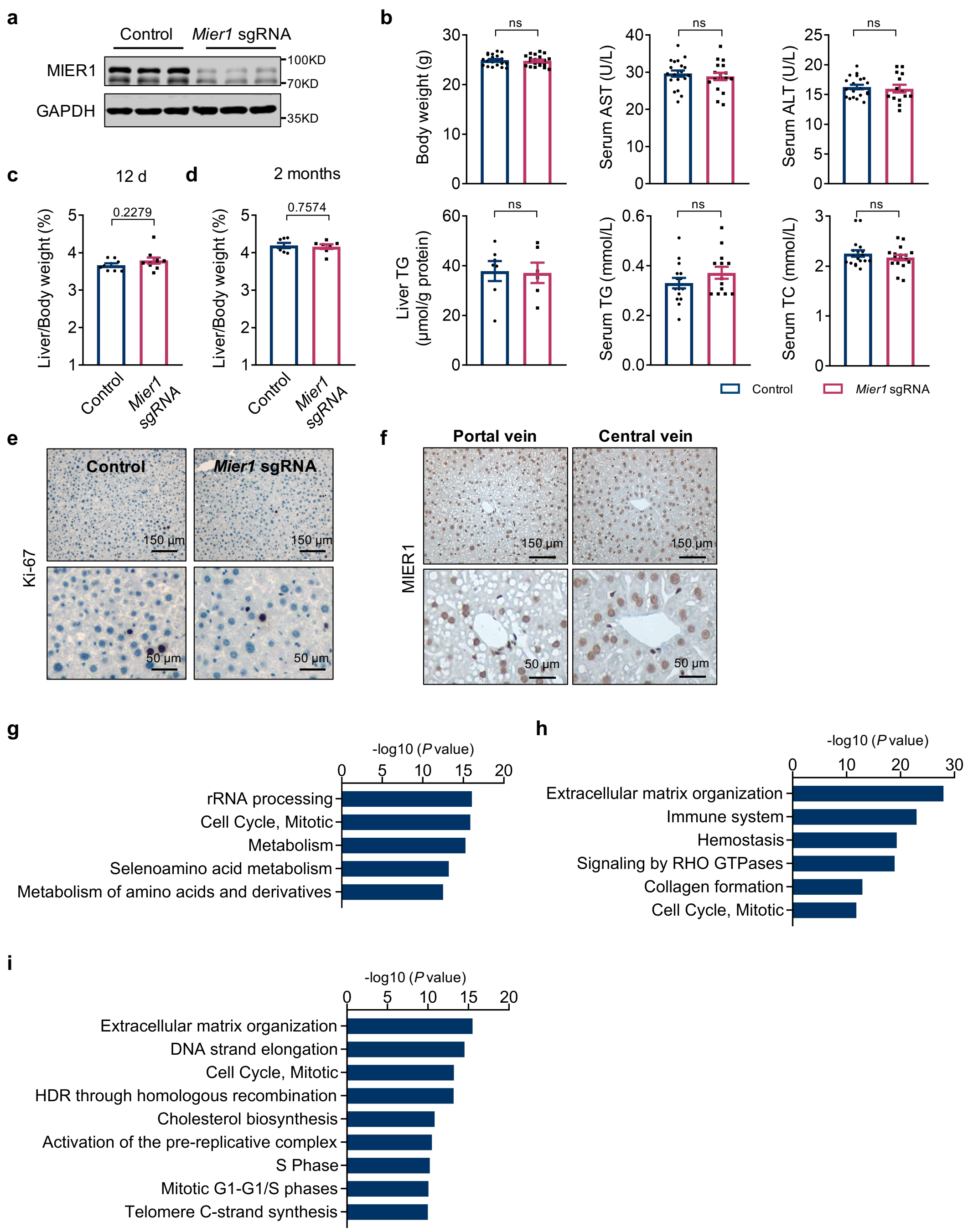

Supplementary Figure 2 Characterization of animals after hepatic MIER1 depletion during regeneration. (**a**) Liver immunoblot of MIER1 in Control and *Mier1* sgRNA animals. (**b**) Measurement of body weight (n = 20), aspartate aminotransferase (AST) (Control, n = 20; *Mier1* sgRNA, n = 17), alanine aminotransferase (ALT) (Control, n = 20; *Mier1* sgRNA, n = 14), liver triglycerides (TG) (Control, n = 7; *Mier1* sgRNA, n = 6), serum TG (Control, n = 15; *Mier1* sgRNA, n = 13), and serum total cholesterol levels (Control, n = 18; *Mier1* sgRNA, n = 17) in control and *Mier1* sgRNA animals. (**c-e**) The liver/body

weight ratio (%) at 12 days (**c**) (Control, n = 9; *Mier1* sgRNA, n = 10) or 2 months (**d**) (n = 7), and liver Ki-67 immunostaining at 2 months (**e**) after partial hepatectomy in Control and *Mier1* sgRNA animals. (**f**) Liver MIER1 immunostaining at 36 h after partial hepatectomy in Control animals. (**g-i**) Gene enrichment analysis of differentially expressed genes in liver collected at 36 h (**g**), 48 h (**h**) and 72 h (**i**) after hepatectomy from Control and *Mier1* sgRNA animals. Values represent means with SEM. *P* values were assessed by unpaired, two-tailed Student's *t*-test (**b, c, d**) or two-tailed Fisher's exact test **(g, h, i)**. Source data are provided as a Source Data file.

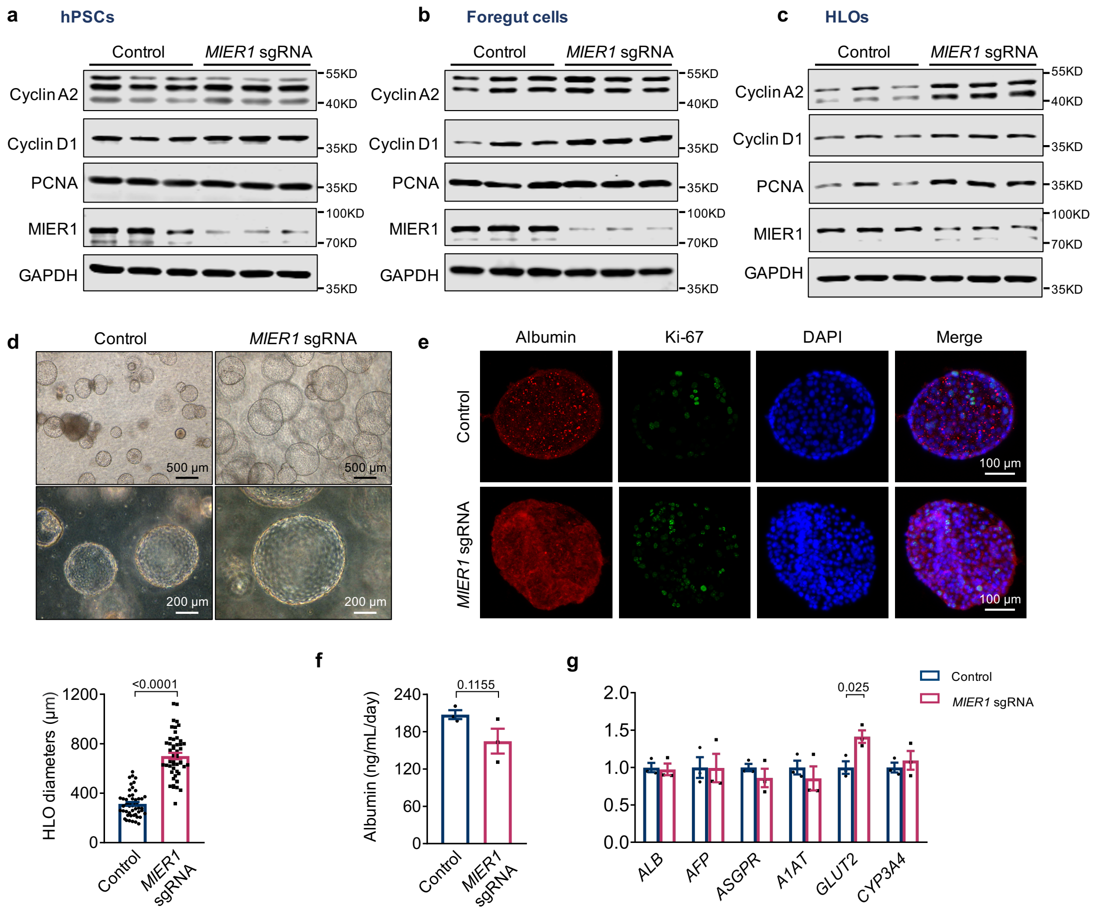

Supplementary Figure 3 MIER1 depletion promotes cell proliferation in human liver organoids (HLOs) derived from human pluripotent stem cells (hPSCs). (**a-c**) Immunoblots of cell cycle factors (Cyclin A2, Cyclin D1, PCNA, MIER1) in hPSCs (**a**), foregut cells after 6 days' differentiation (**b**), and HLOs after 24 days' differentiation (**c**). (**d**) Size calculation of HLOs in different groups as indicated. n = 50. (**e**) Immunostaining for Ki-67 and albumin in HLOs. (**f**) Measurement of albumin secretion in HLOs. n = 3. (**g**) Expression levels of several hepatic markers of HLOs. n = 3. Values represent means with SEM. *P* values were assessed by unpaired, two-tailed Mann-Whitney test (**d**) or unpaired, two-tailed Student's *t*-test (**f, g**). Source data are provided as a Source Data file.

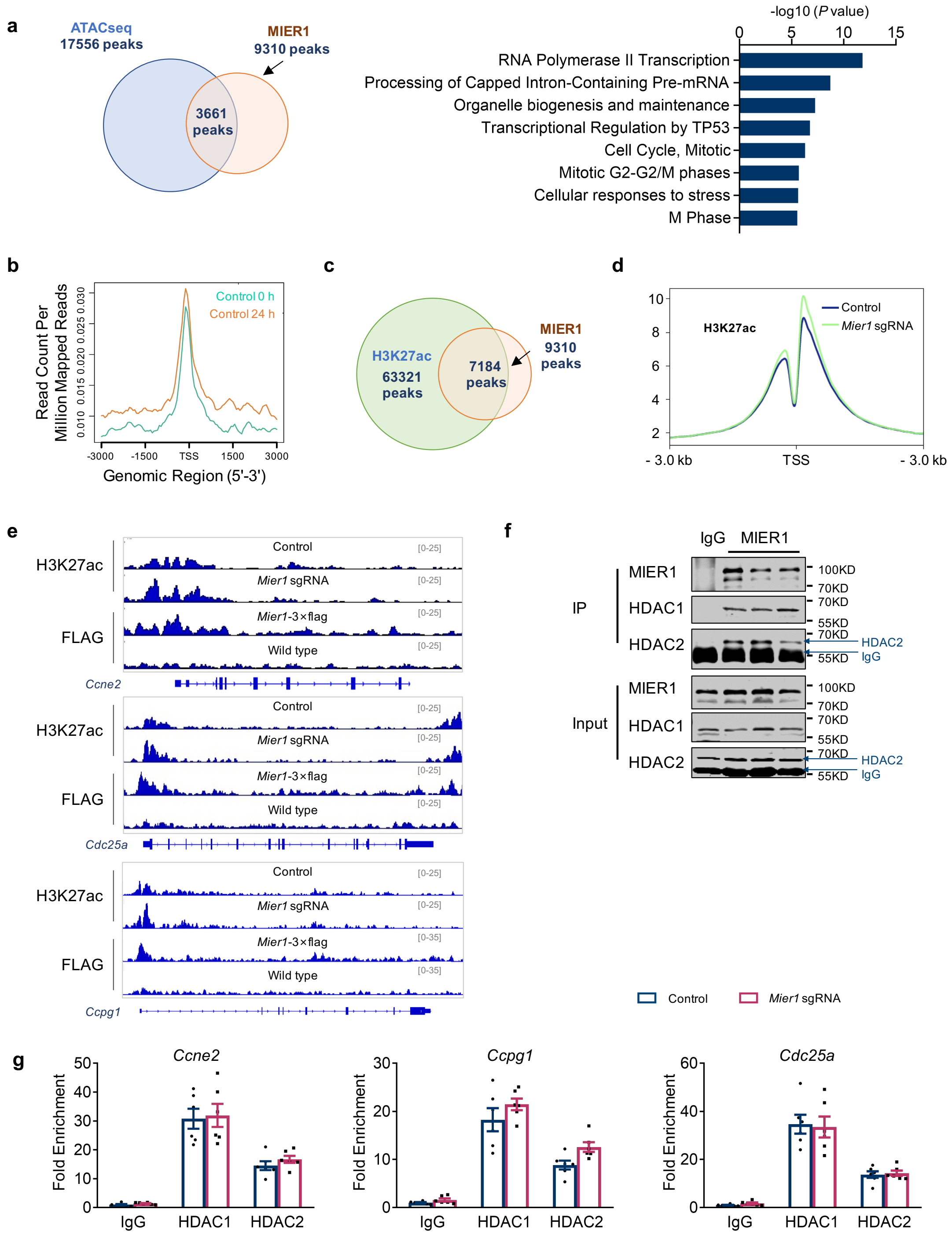

Supplementary Figure 4 MIER1 targets and regulates cell cycle gene expression through chromatin remodeling. (a) Overlap of ATAC-seq peaks and MIER1-bound peaks in quiescent liver tissues (left); Gene enrichment analysis of genes near overlapped peaks (right). (**b**) ATAC-seq signals in liver tissues before or 24 h after surgery. (**c**) Overlap peaks of liver MIER1 with H3K27ac cistrome. (**d**) Average intensity of H3K27ac signals surrounding the transcription start site (TSS) in Control and *Mier1* sgRNA groups at 24 h post

hepatectomy. (**e**) ChIP-seq tracks of MIER1 and H3K27ac signals near liver *Ccne2, Cdc25a,* and *Ccpg1* genes in different groups as indicated. (**f**) Interaction analysis of MIER1 and HDAC1 and HDAC2 in liver tissue. (**g**) ChIP-qPCR analysis of HDAC1 and HDAC2 near several cell cycle genes in liver tissues from 24 h after surgery. n = 6. Values represent means with SEM. *P* values were assessed by two-tailed Fisher's exact test **(a)**. Source data are provided as a Source Data file.

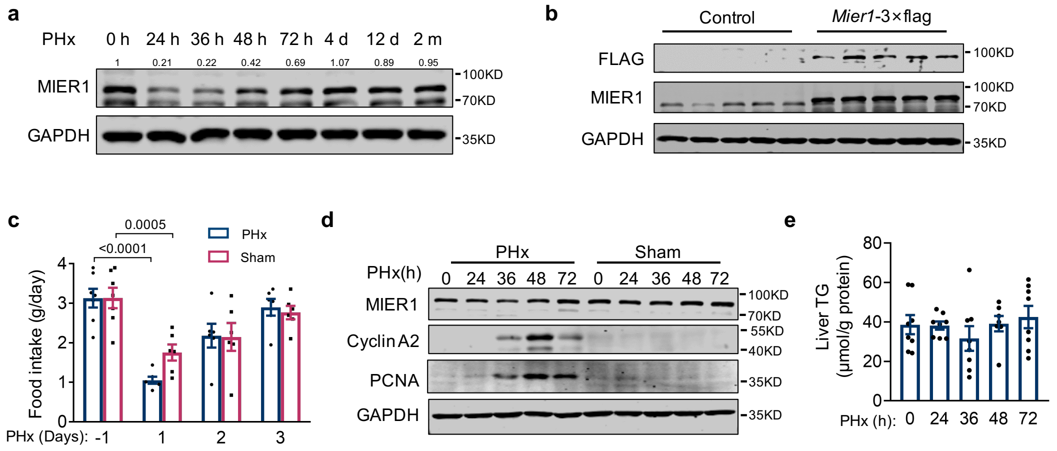

Supplementary Figure 5 MIER1 shows a transient decrease in early liver regeneration. (**a**) Immunoblot of liver MIER1 before or at different time points after hepatectomy**.** (**b**) Immunoblot of exogenous MIER1 as determined by both FLAG and MIER1 antibodies**.** (**c**) Food intake analysis in animals before or after liver hepatectomy or sham surgery (n = 7, 6, 6, 6 at different time points). (**d**) Liver immunoblots (MIER1, Cyclin A2, PCNA, and GAPDH) in groups as in (**c**). (**e**) Liver triglyceride analysis in animals with sham surgery (n = 8, 8, 8, 7, 8 at different time points). Values represent means with SEM. *P* values were assessed by Two-Way ANOVA with *post hoc*Šídák's multiple comparisons test (**c**). Source data are provided as a Source Data file.

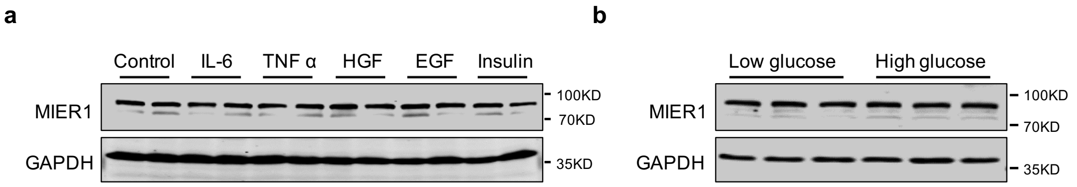

c

Conc. (mg/g)	TAG	SM	PE	PC	LPE	LPC	HexCer	Hex2Cer	FFA	DAG	Cer	CE
**0h**	10.00832	1.137107	2.752532	17.63879	0.137463	0.159877	0.035041	0.003128	3.649549	2.016099	0.356144	1.689411
**24h**	79.47927	1.297691	3.706209	19.07111	0.152102	0.172028	0.068925	0.006992	4.205505	3.72701	0.45412	4.901113

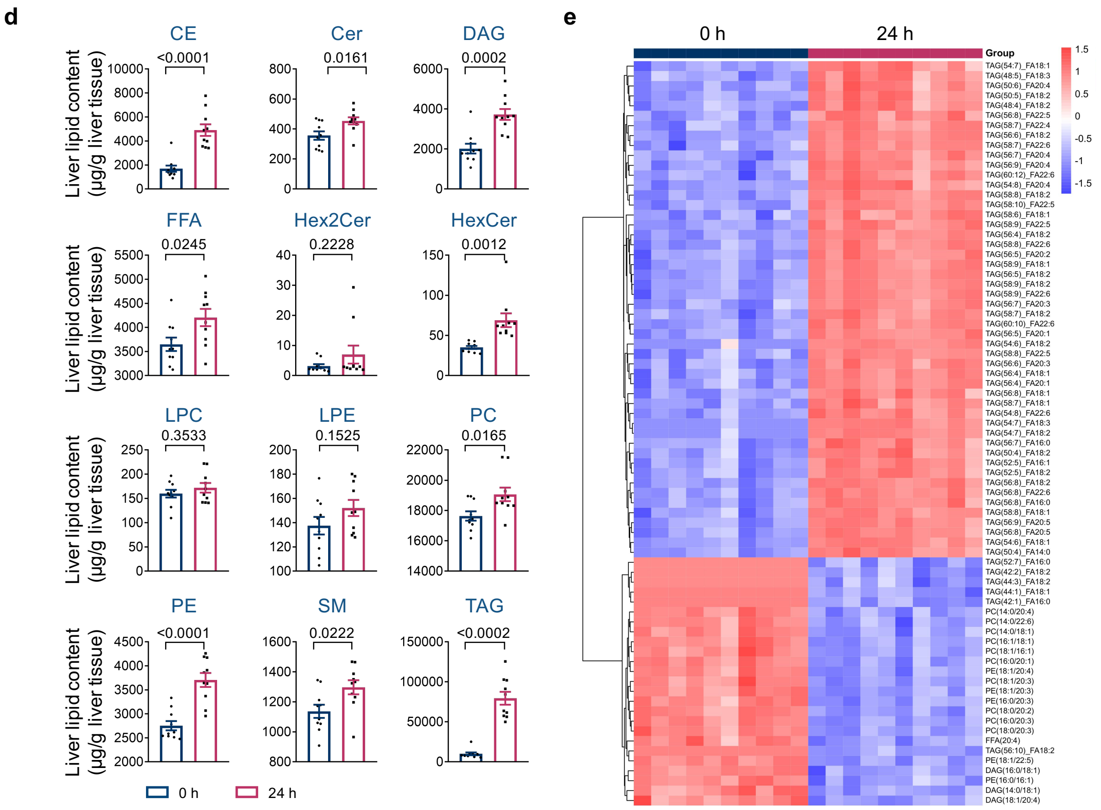

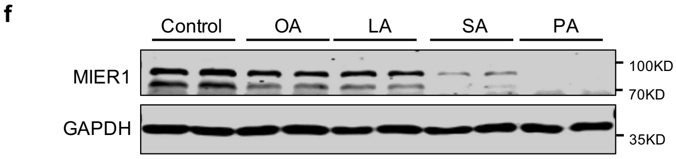

Supplementary Figure 6 Acute hepatic lipid accumulation regulates MIER1 expression during liver regeneration. (**a-b**) Immunoblot of MIER1 in primary hepatocytes after treatment with interleukin-6 (30 ng/mL), TNF α (30 ng/mL), HGF (50 ng/mL), EGF (50 ng/mL) and insulin (10 μM) for 10 h (**a**), or cultured in medium with low (1.0 g/L) or high (4.5 g/L) glucose for 10 h (**b**). (**c, d**) Quantification of different lipid species in liver tissues from NCD animals at 0 h and 24 h after PHx (n = 10). (**e**) The heatmap showing the top 75 differentially regulated lipids between liver tissues before and 24 h after

hepatectomy. Values present as fold change over the average value of each lipid in both groups. (**f**) Immunoblot of MIER1 in primary hepatocytes after treatment with 1 mM oleic acid (OA), linoleic acid (LA), stearic acid (SA) and palmitic acid (PA) for 10 h. Values represent means with SEM. *P* values were assessed by unpaired, two-tailed Student's *t*-test (**d**). Source data are provided as a Source Data file.

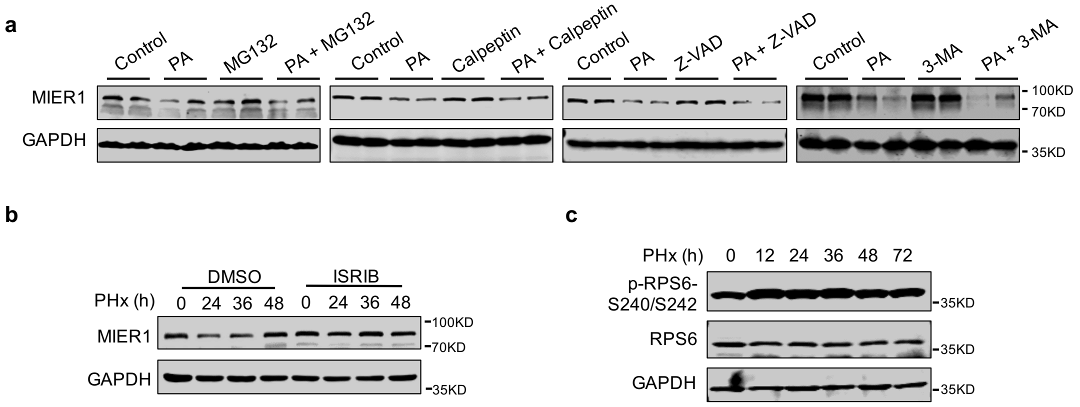

Supplementary Figure 7 Acute hepatic steatosis does not regulate MIER1 expression through protein stabilization. (**a**) Immunoblots of MIER1 in primary hepatocytes after treatment with 0.5 mM PA with or without MG132 (25 μM), Calpeptin (10 μM), Z-VAD (25 μM) and 3-MA (5 mM). (**b**) Liver immunoblots (MIER1 and GAPDH) in animals before and after liver hepatectomy, treated with DMSO or ISRIB. (**c**) Immunoblots of liver RPS6 phosphorylation before and after liver hepatectomy. Source data are provided as a Source Data file.

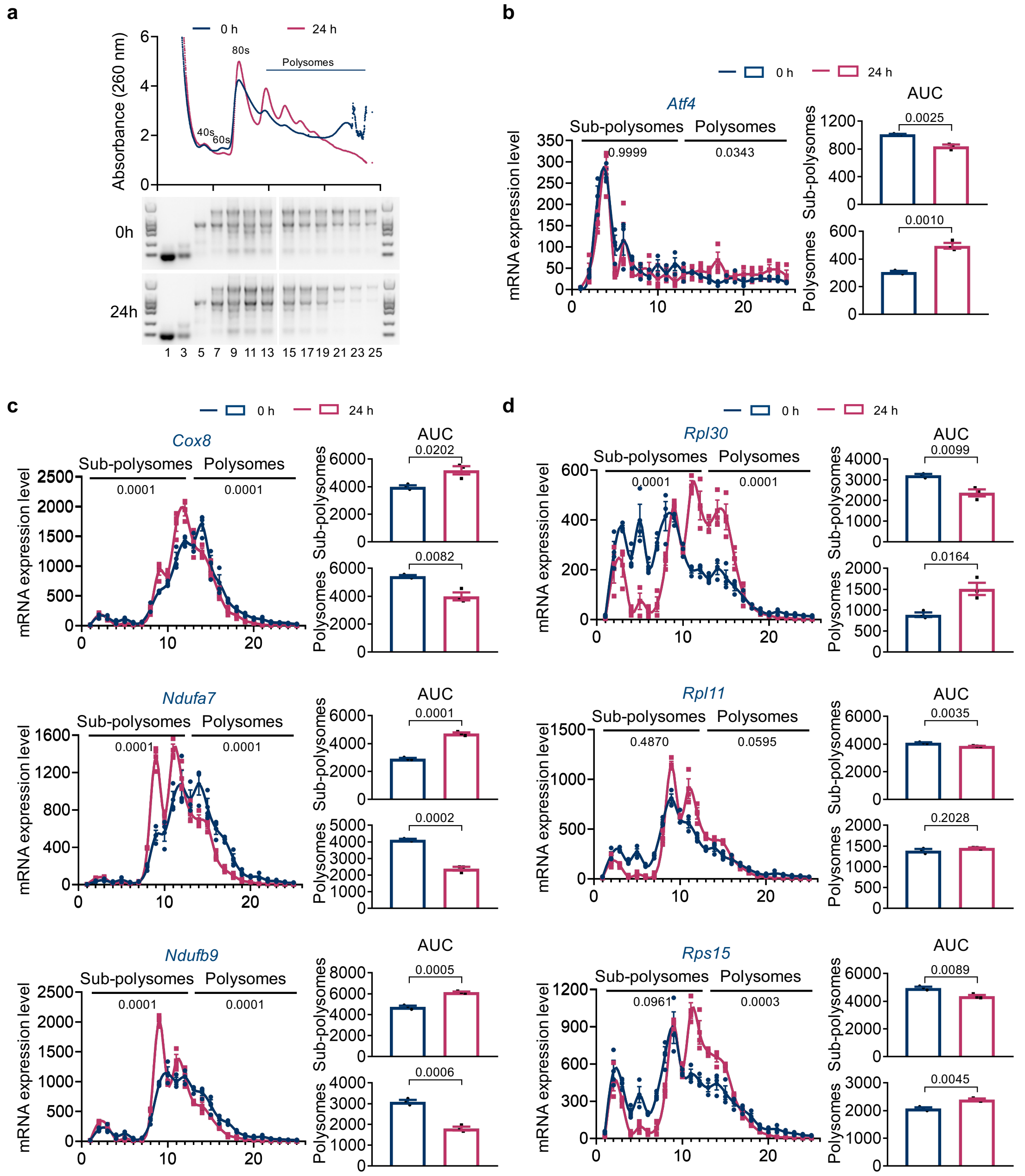

Supplementary Figure 8 Transient hepatic steatosis regulates MIER1 expression via acute stress-induced translational control. (**a**) Representative polysome profiles of liver tissues in animals before or 24 h after liver hepatectomy. Upper: the absorbance values in OD260 nm; Below: gel picture of RNAs in each fraction. **(b)** qRT-PCR analysis of the distribution of *Atf4* transcripts in liver tissues collected before or 24 h after surgery. n = 3. **(c-d)** qRT-PCR analysis of the distribution of representative mRNA transcripts with decreased (**c**) (n = 3) and increased (**d**) (n = 3) translational activity in liver tissues collected before or 24 h after hepatectomy. Values represent means

with SEM. *P* values were assessed by unpaired, two-tailed Student's *t*-test (**b, c, d**). Source data are provided as a Source Data file.

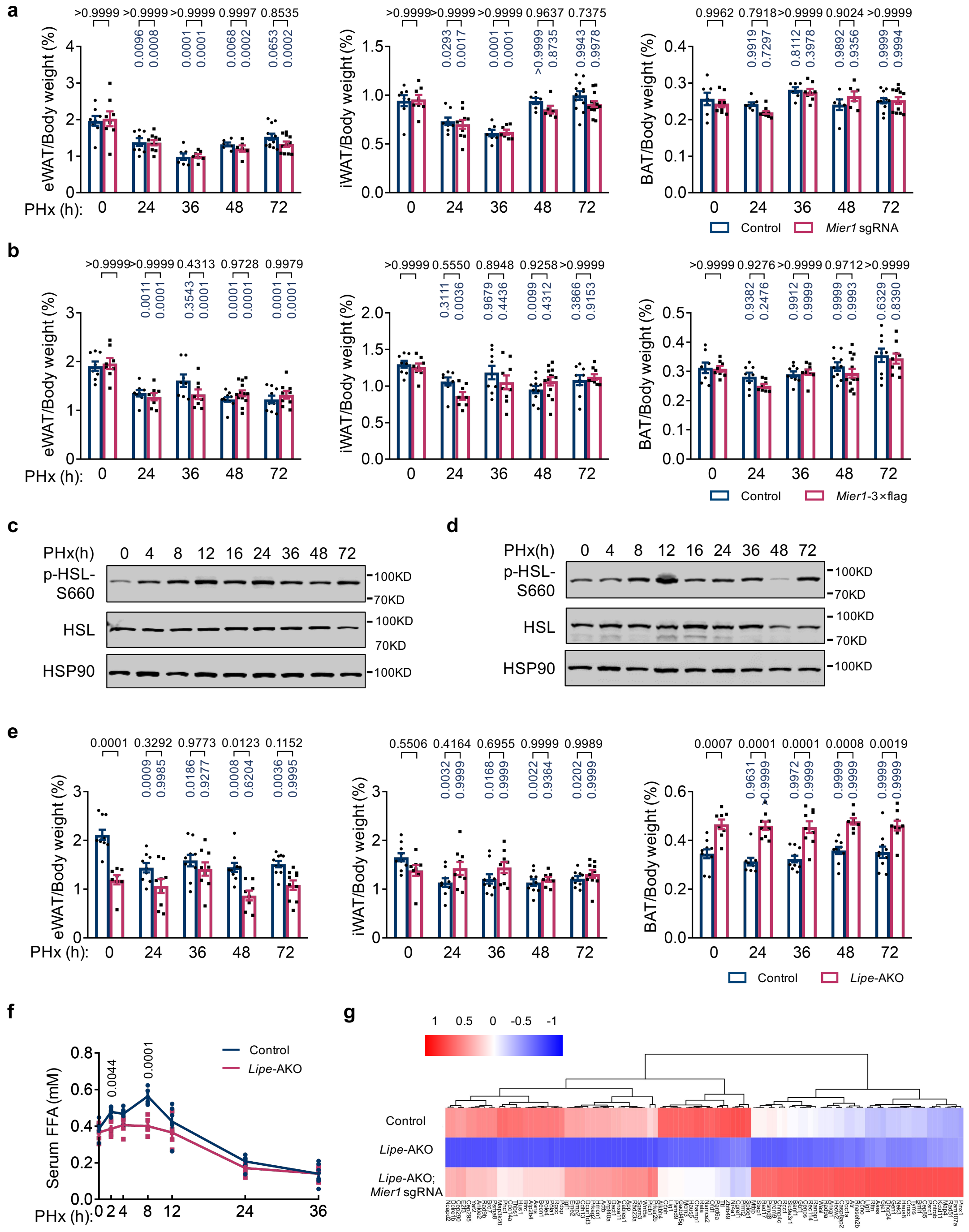

Supplementary Figure 9 Analyses of adipose catabolism during liver regeneration between different animal models. (**a-b**) Ratio of eWAT / Body weight (%), iWAT / Body weight (%), and BAT / Body weight (%), before and after hepatectomy in Control and *Mier1* sgRNA animals (**a**) (n = 8, 8, 7, 6, 12 in Control group, and n = 8, 10, 7, 6, 11 in *Mier1* sgRNA-treated group), or Control and *Mier1*-3×flag animals (**b**) (n = 8, 8, 8, 10, 9 in Control group, and n = 8, 8, 8, 13, 9 in *Mier1*-3×flag group). (**c-d**) Immunoblots (p-HSL-S660, HSL and HSP90) in epididymal white adipose tissues (**c**) and inguinal white adipose

tissues (**d**) from animals before or after surgery. (**e**) Ratio of eWAT / Body weight (%), iWAT / Body weight (%), and BAT/ Body weight (%), before and after hepatectomy in Control and *Lipe*-AKO animals (n = 10, 9, 10, 10, 10 in Control group, and n = 8, 9, 9, 8, 10 in *Lipe*-AKO group). (**f**) Serum free fatty acid (FFA) levels before and after hepatectomy in Control (n = 7) and *Lipe*-AKO animals (n = 6). (**g**) Heatmap presentation of top 100 differentially expressed cell cycle-relevant genes in livers from Control, *Lipe*-AKO, and *Lipe*-AKO; *Mier1* sgRNA animals at 36 h after surgery. Values represent means with SEM. *P* values were assessed by Two-Way ANOVA with *post hoc* Tukey's multiple-comparison tests. *P* values in blue show the comparison between ratio in different time points after hepatectomy and the one before surgery in the same group (**a**, **b**, **e**). Source data are provided as a Source Data file.

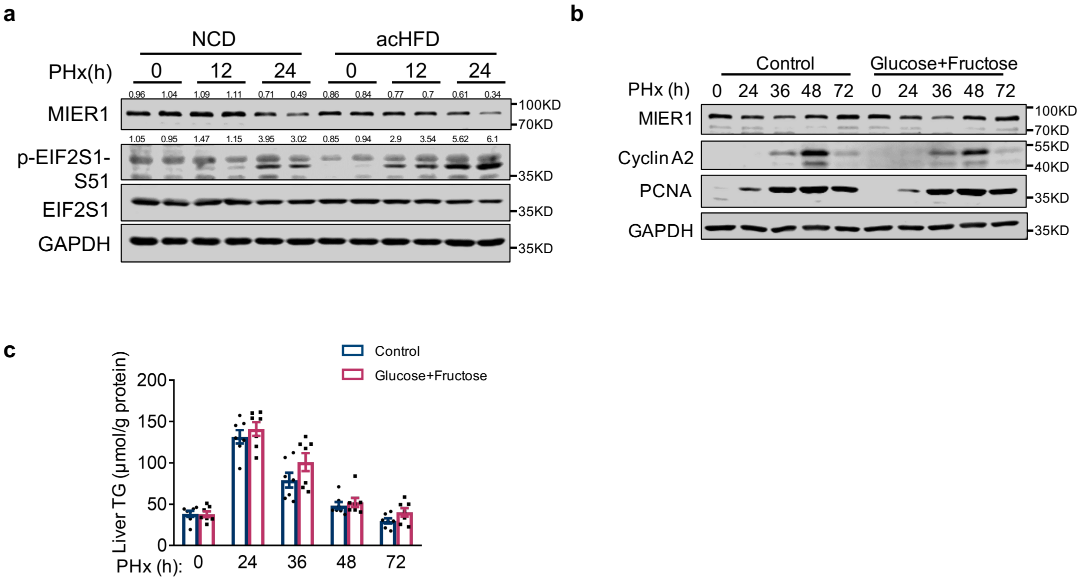

Supplementary Figure 10 Acute HFD treatment improves liver regeneration. (**a**) Immunoblots of liver MIER1 and EIF2S1 phosphorylation before or at different time points after hepatectomy in NCD and acHFD animals. (**b, c**) Liver immunoblots (MIER1, Cyclin A2, PCNA and GAPDH) (**b**) and liver triglyceride measurement (**c**) (n = 7) in control animals or animals pre-fed with glucose and fructose before and after liver hepatectomy. Values represent means with SEM. Source data are provided as a Source Data file.

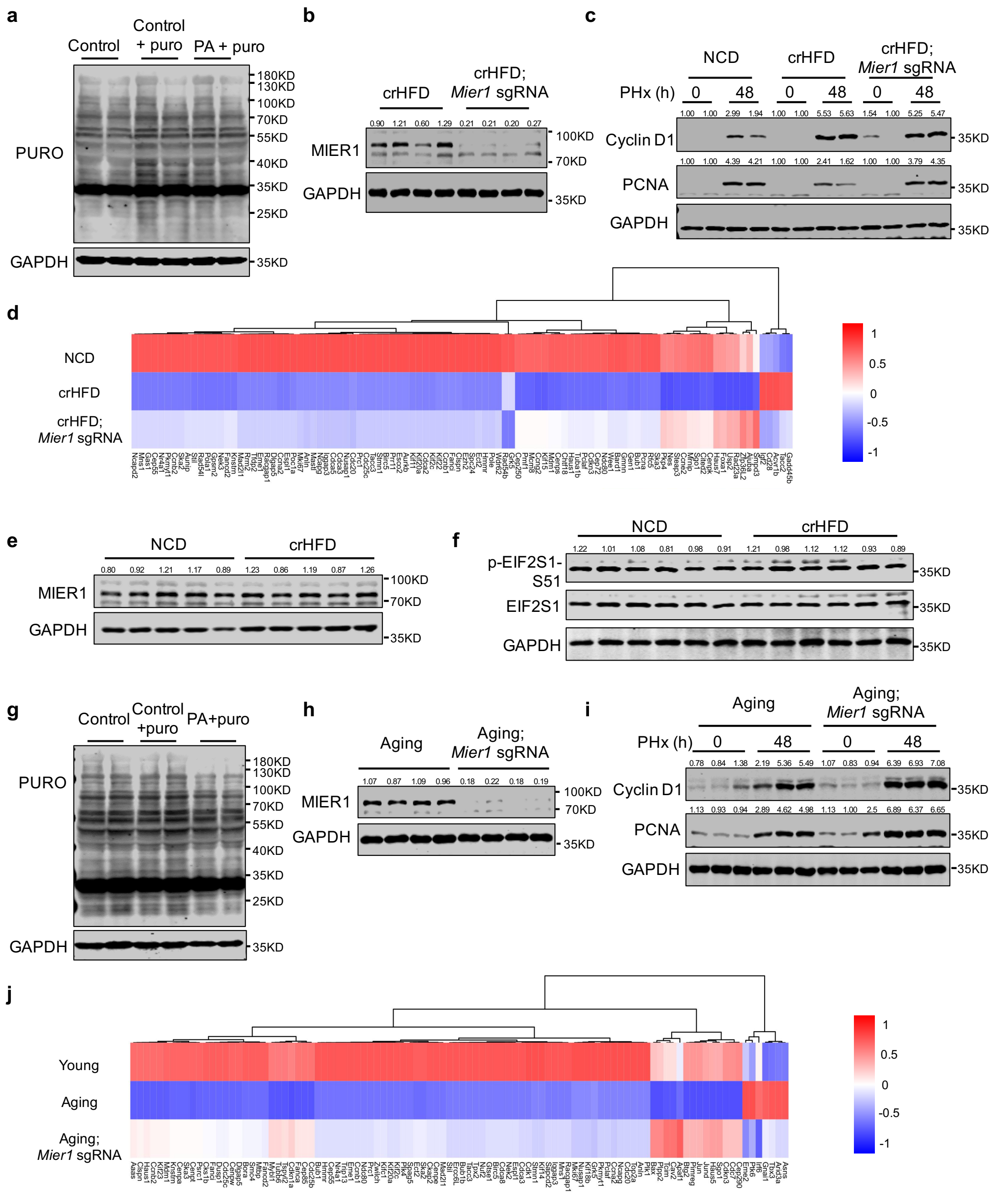

Supplementary Figure 11 Animals under long time HFD feeding and aging animals show dysregulated MIER1 expression and improved regeneration upon MIER1 depletion. (**a**) Protein synthesis analysis in primary hepatocytes derived from crHFD animals treated with or without 0.5 mM PA for 10 h. (**b**) Immunoblot of endogenous liver MIER1 in crHFD animals administrated with AAV-*Cre-2a-Luci* (Control) or AAV-*Cre-2a-Luci-Mier1* sgRNA (*Mier1* sgRNA). (**c**) Immunoblots of liver Cyclin D1 and PCNA before or 48 h after hepatectomy in NCD, crHFD, and crHFD; *Mier1* sgRNA animals. (**d**) Heatmap presentation of top 100 differentially expressed cell cycle-relevant

genes in livers from NCD, HFD, and HFD; *Mier1* sgRNA animals at 48 h after surgery. (**e-f**) Immunoblots of liver MIER1 (**e**) and EIF2S phosphorylation (**f**) in NCD and crHFD animals. (**g**) Protein synthesis analysis in primary hepatocytes derived from aging animals with or without treatment of 0.5 mM PA for 10h. (**h**) Immunoblots of endogenous liver MIER1 levels in aging animals administrated with AAV-*Cre-2a-Luci* (Control) or AAV-*Cre-2a-Luci-Mier1* sgRNA (*Mier1* sgRNA). (**i**) Immunoblots of liver Cyclin D1 and PCNA before or 48h after hepatectomy in Aging and Aging; *Mier1* sgRNA animals. (**j**) Heatmap presentation of top 100 differentially expressed cell cycle-relevant genes in livers from Young, Aging, and Aging; *Mier1* sgRNA animals at 48 h after surgery. Source data are provided as a Source Data file.

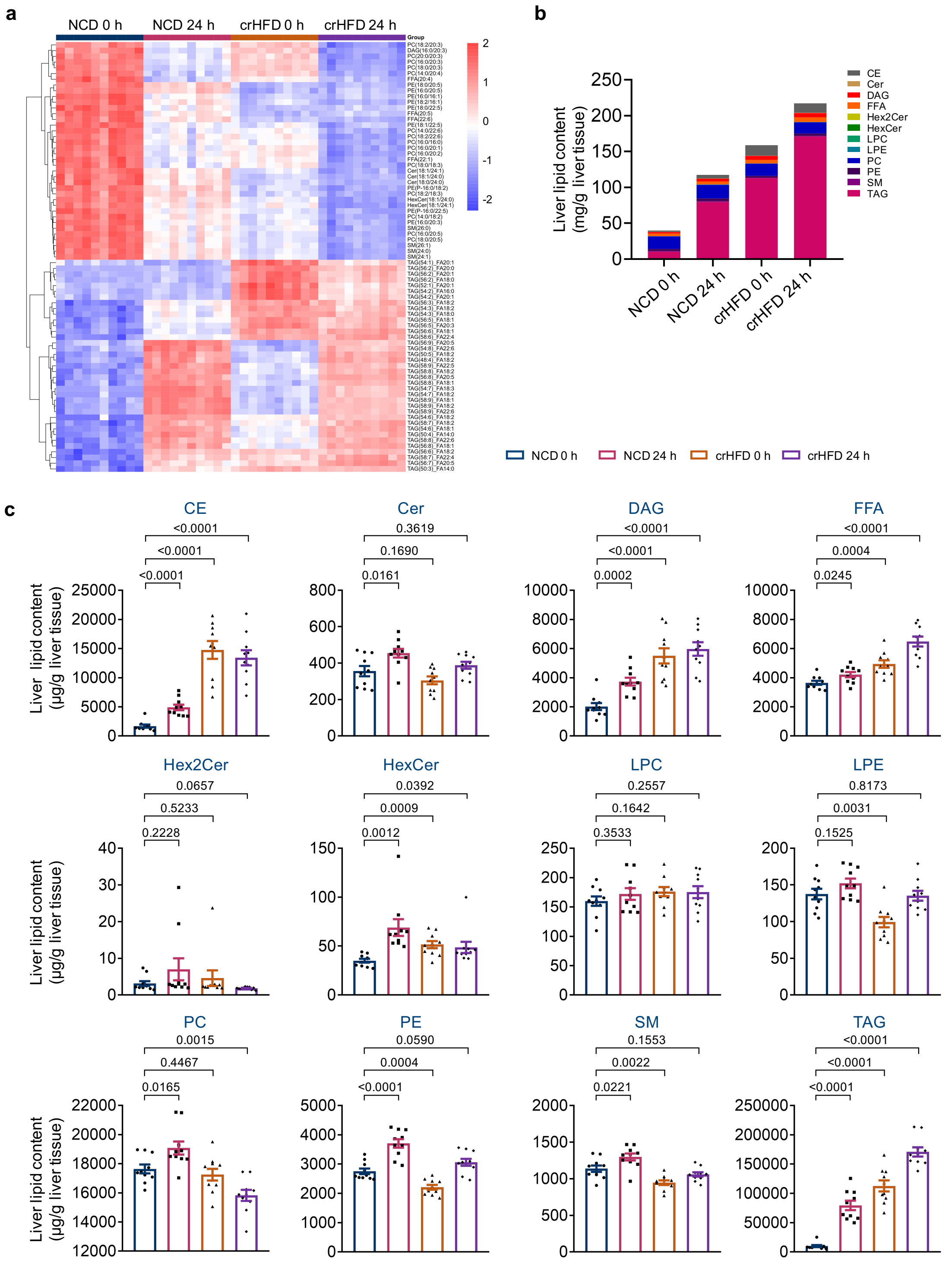

Supplementary Figure 12 Lipid content analysis in liver tissues from NCD or crHFD-fed animals at 0 h and 24 h after PHx.

(a) The heatmap showing the top 75 differentially regulated lipids between liver tissues from different groups as indicated. Values present as fold change over the average value of each lipid in all groups. (**b-c**) Quantification of different lipid species in liver tissues from groups as in (**a**) (n = 10). Values represent

means with SEM. *P* values were assessed by One-Way ANOVA with Dunnett's multiple comparisons test. Source data are provided as a Source Data file.
