# Supplementary material for: Acute liver steatosis translationally controls the epigenetic regulator MIER1 to promote liver regeneration in a study with male mice
Source: Nat Commun. 2023 Mar 18;14:1521. doi: 10.1038/s41467-023-37247-9 (PMC10024732; doi:10.1038/s41467-023-37247-9)
Supplement: Supplementary file 3 — Description of Additional Supplementary Files [file 41467_2023_37247_MOESM3_ESM.docx]

**Description of Additional Supplementary Files**

File Name: Supplementary Data 1.

Description: The scores of each gene in two independent CRISPR screens.

File Name: Supplementary Data 2.

Description: List of genes targeted by MIER1 and upregulated in MIER1-depleted liver tissues as compared to wild-type tissues at 24 h after partial hepatectomy.

File Name: Supplementary Data 3.

Description: The lipidomic analyses in liver tissues collected from NCD and crHFD animals before and 24 h after partial hepatectomy. The results are displayed as ug/g tissue.

File Name: Supplementary Data 4.

Description: Calculation of the fatty acid composition in all lipid species from the lipidomic analyses.

File Name: Supplementary Data 5.

Description: The differential genes identified in the RNA-seq analyses of polysome and sub-polysome-associated RNA transcripts before and 24 h after partial hepatectomy. A cutoff of *P* < 0.05 was used to generate this list.
