# Supplementary material for: Acute liver steatosis translationally controls the epigenetic regulator MIER1 to promote liver regeneration in a study with male mice
Source: Nat Commun. 2023 Mar 18;14:1521. doi: 10.1038/s41467-023-37247-9 (PMC10024732; doi:10.1038/s41467-023-37247-9)
Supplement: Supplementary file 9 — Reporting Summary [file 41467_2023_37247_MOESM9_ESM.pdf]

nature portfolio

Corresponding author(s): *Double-blind peer review submissions: write DBPR and your manuscript number here instead of author names.*

Last updated by author(s): *YYYY-MM-DD*

Reporting Summary

Nature Portfolio wishes to improve the reproducibility of the work that we publish. This form provides structure for consistency and transparency in reporting. For further information on Nature Portfolio policies, see our Editorial Policies and the Editorial Policy Checklist.

Statistics

For all statistical analyses, confirm that the following items are present in the figure legend, table legend, main text, or Methods section.

n/a	Confirmed	
☐	☒	The exact sample size (*n*) for each experimental group/condition, given as a discrete number and unit of measurement
☐	☒	A statement on whether measurements were taken from distinct samples or whether the same sample was measured repeatedly
☐	☒	The statistical test(s) used AND whether they are one- or two-sided<br>*Only common tests should be described solely by name; describe more complex techniques in the Methods section.*
☒	☐	A description of all covariates tested
☐	☒	A description of any assumptions or corrections, such as tests of normality and adjustment for multiple comparisons
☐	☒	A full description of the statistical parameters including central tendency (e.g. means) or other basic estimates (e.g. regression coefficient) AND variation (e.g. standard deviation) or associated estimates of uncertainty (e.g. confidence intervals)
☐	☒	For null hypothesis testing, the test statistic (e.g. *F*, *t*, *r*) with confidence intervals, effect sizes, degrees of freedom and *P* value noted<br>*Give P values as exact values whenever suitable.*
☒	☐	For Bayesian analysis, information on the choice of priors and Markov chain Monte Carlo settings
☒	☐	For hierarchical and complex designs, identification of the appropriate level for tests and full reporting of outcomes
☒	☐	Estimates of effect sizes (e.g. Cohen's *d*, Pearson's *r*), indicating how they were calculated

Our web collection on statistics for biologists contains articles on many of the points above.

Software and code

Policy information about availability of computer code

Data collection

1. q-PCR: ABI Q6
2. Western blot: Bio-Rad and LI-COR Odyssey DLX
3. In vivio CRISPR screening: Illumina HiSeq4000
3. ChIP-seq: NextSeq 500
4. RNA-seq: Illumina novaseq 6000
5. ATAC-seq: Illumina Hiseq X Ten
6. Flow cytometry: FACSAria II
7. Lipidomics: SCIEX ExionLC UPLC
8. Tissue section images: Olympus, FV1200
9. Immunofluorescence images: Zeiss, LSM880

Data analysis

1. Statistical analysis: Graphpad prism 8.0
2. Western blot: Image Studio Lite Ver 5.2
3. Immunofluorescence images analysis: ImageJ-Fiji 2.9.0
4. Flow cytometry: FlowJo 10.0.7
4. RNA-seq: Illumina Casava1.8 software used for basecalling. Sequenced reads were trimmed for adaptor sequence, and masked for low-complexity or low-quality sequence, then mapped to Mus musculus whole genome using hisat v2.1.0 with default parameter. The expression level of each transcript was calculated according to the fragments per kilobase of exon per million mapped reads (FRKM) method. RSEM v1.2.31 was used to quantify gene abundances. Differential gene expression analysis was done using R package DESeq2 v1.10.1.
5. ChIP-seq: Raw data were mapped to the genome (mm10) using the BWA algorithm with default settings. Peaks were called using MACS algorithms (MACS 2.1.0) with an irreproducible discovery rate (IDR) of <0.05. Peak filtering was performed by removing false ChIP-Seq peaks

as defined with the ENCODE blacklist by bedtools subtract function (bedtools 2.30.0).
6. ATAC-seq: Basecalls were performed using bcl2fastq v2.17. ATAC-seq reads were trimmed by using Trim Galore (v0.6.7), discarding reads left with < 20 bp, high quality reads were picked by SAMtools (v1.7). ATAC-seq reads were aligned to the reference mouse genome UCSC mm10 by Bowtie 2 (v2.2.5). Only uniquely mapping reads without mismatches were retained. Duplicate reads and reads mapping to the mitochondrial genome were discarded. ATAC-seq peaks were called and annotated by using HOMER (v4.11) findPeaks function with following parameters "-style dnase -minDist 200 -size 100". Peak regions overlapping with mm10 ENCODE exclusion list regions were discarded. For visualization purposes, deepTools (v3.5.1) was used to normalize ATAC-seq read counts. Average profiles of regions of interest in each group were performed by using ngs.plot (v2.61)
7. Lipidomic Analysis: All peaks and metabolites were left after relative standard deviation de-noising. The missing values were then filled up by half of the minimum value, with normalization method also employed in this data analysis. The final dataset containing the information of peak number, sample name and normalized peak area was further imported to the SIMCA16.0.2 software package for multivariate analysis.

For manuscripts utilizing custom algorithms or software that are central to the research but not yet described in published literature, software must be made available to editors and reviewers. We strongly encourage code deposition in a community repository (e.g. GitHub). See the Nature Portfolio guidelines for submitting code & software for further information.

Data

Policy information about availability of data

All manuscripts must include a data availability statement. This statement should provide the following information, where applicable:

- Accession codes, unique identifiers, or web links for publicly available datasets
- A description of any restrictions on data availability
- For clinical datasets or third party data, please ensure that the statement adheres to our policy

All data associated with this study are presented in the paper or the Supplementary Materials. Source data are also provided with this paper. The high-throughput sequencing data for this study have been deposited in Gene Expression Omnibus and Sequence Read Archive at National Center for Biotechnology Information. The accession number for the CRISPR screening data reported in this paper is SRR16916496 ([https://www.ncbi.nlm.nih.gov/sra/?term=SRR16916496XXX). The accession numbers for the RNA-seq data reported in this paper are GSE188421 ([https://www.ncbi.nlm.nih.gov/geo/query/acc.cgi?acc=GSE188421),], GSE188768 ([https://www.ncbi.nlm.nih.gov/geo/query/acc.cgi?acc=GSE188768),], and GSE212628 ([https://www.ncbi.nlm.nih.gov/geo/query/acc.cgi?acc=GSE212628).]. The accession numbers for the ChIP-seq data reported in this paper are GSE188747 ([https://www.ncbi.nlm.nih.gov/geo/query/acc.cgi?acc=GSE188747)] and GSE188742 ([https://www.ncbi.nlm.nih.gov/geo/query/acc.cgi?acc=GSE188742).]. The accession numbers for the ATAC-seq data reported in this paper are GSE212626 ([https://www.ncbi.nlm.nih.gov/geo/query/acc.cgi?acc=GSE212626).]. The lipidomic data for this study have been deposited in The National Omics Data Encyclopedia. The accession number for lipidomic data is OEP003146 [https://www.biosino.org/node/project/detail/OEP003146]. The mouse genome (GRCm38/mm10) data used in this study are available in the UCSC Genome Browser [https://hgdownload.soe.ucsc.edu/goldenPath/mm10/bigZips/]

Human research participants

Policy information about studies involving human research participants and Sex and Gender in Research.

Reporting on sex and gender	N/A
Population characteristics	N/A
Recruitment	N/A
Ethics oversight	N/A

Note that full information on the approval of the study protocol must also be provided in the manuscript.

Field-specific reporting

Please select the one below that is the best fit for your research. If you are not sure, read the appropriate sections before making your selection.

☒ Life sciences ☐ Behavioural & social sciences ☐ Ecological, evolutionary & environmental sciences

For a reference copy of the document with all sections, see nature.com/documents/nr-reporting-summary-flat.pdf

Life sciences study design

All studies must disclose on these points even when the disclosure is negative.

Sample size	For animals studies, we did not predetermine the sample sizes but used group sizes typically for this type of work on basis of previous experiments using similar methodologies. To minimize any potential bias, we randomly assigned mice of same genotype to different treatments. At least 6 mice per group at each time point were operated to ensure adequate sample size. In the HLO experiments, all differentiations were repeated four times with similar results. All the Western blot experiment was repeated at least three times from different samples with similar results. All the micrographs were taken from at least four samples with similar results.
Data exclusions	No data was excluded.

Replication	All experimental data were reproduced in multiple independent experiments as indicated in the figure legends.
Randomization	For cell experiments, samples were randomly allocated to each group. For animal experiments, mice were randomly assigned to control and experimental groups.
Blinding	Experiments performance was not completely blinded. Because each operation lasts 3-5h, followed by the sacrifice of mice in a short time, in order to reduce the effect of biorhythms on mice, the control group and the experimental group of mice alternately perform surgery. However, when the sample was processed, the experimenter only knew the number of the mice, and did not know the grouping of the number. Meantime, standard laboratory procedures of randomization were followed. Each Experiment was associated with proper controls, and compared samples were collected and analyzed under the same conditions.

Reporting for specific materials, systems and methods

We require information from authors about some types of materials, experimental systems and methods used in many studies. Here, indicate whether each material, system or method listed is relevant to your study. If you are not sure if a list item applies to your research, read the appropriate section before selecting a response.

Materials & experimental systems

n/a	Involved in the study
☐	☒ Antibodies
☐	☒ Eukaryotic cell lines
☒	☐ Palaeontology and archaeology
☐	☒ Animals and other organisms
☒	☐ Clinical data
☒	☐ Dual use research of concern

Methods

n/a	Involved in the study
☐	☒ ChIP-seq
☐	☒ Flow cytometry
☒	☐ MRI-based neuroimaging

Antibodies

Antibodies used

GFP (Invitrogen, A-21311),
FAH (Abcam, ab83770),
Ki-67 (Cell Signaling Technology, 12202S),
H3K27ac (Active Motif, 39133),
MIER1 (Sigma, HPA019589),
MIER1 (Proteintech, 11452-1-AP),
GAPDH (ABclonal, AC002),
GAPDH (Proteintech, 60004-1-Ig),
PCNA (Proteintech, 10205-2-AP),
PCNA (Proteintech, 60097-1 -Ig),
Cyclin A2 (Proteintech, 18202-1-AP),
Cyclin D1 (ABclonal, A0310),
HDAC1 (Abcam, ab7028),
HDAC2 (Abcam, ab12169),
HDAC1 (Cell Signaling Technology, 34589S),
HDAC2 (Cell Signaling Technology, 57156S),
FLAG (Sigma, F7425),
puromycin (Millipore, MABE341),
EIF2S1 (ABclonal, A0764),
p-EIF2S1-S51 (Beyotime, AF5803),
RPS6 (ABclonal, A6058),
p-RPS-S240/S242 (ABclonal, AP0537),
HSL (ABclonal, A15686),
p-HSL (ABclonal, AP1242)
HSP90 (Cell Signaling Technology, 4874S),
Albumin (R&D systems, MAB1455).
IRDye 680RD Donkey anti-Mouse (LI-COR Biosciences, 926-68072)
IRDye 800CW Donkey anti-rabbit (LI-COR Biosciences, 925-32213)
Alexa 647 goat-anti-mouse (Invitrogen, A21235)
Alexa 488 donkey-anti-rabbit (Invitrogen, R37118)

Validation

All antibodies were confirmed or the species and application through the validation statement on the manufacturer's website and their use in the literature. We are providing here the list of antibodies used.
GFP (Invitrogen, A-21311), https://www.thermofisher.cn/cn/en/antibody/product/GFP-Antibody-Polyclonal/A-21311
FAH (Abcam, ab83770), https://www.abcam.cn/fumarylacetoacetate-hydrolasefaa-antibody-ab151998.html
Ki-67 (Cell Signaling Technology, 12202S), https://www.cellsignal.cn/products/primary-antibodies/ki-67-d3b5-rabbit-mab-mouse-preferred-ihc-formulated/12202?site-search-type=Products&N=4294956287&Ntt=12202s&fromPage=plp&_requestid=3313810
H3K27ac (Active Motif, 39133),https://www.activemotif.com/catalog/details/39133/histone-h3-acetyl-lys27-antibody-pab
MIER1 (Sigma, HPA019589), https://www.sigmaaldrich.cn/CN/zh/product/sigma/hpa019589
MIER1 (Proteintech, 11452-1-AP), https://www.ptglab.com/products/MIER1-Antibody-11452-1-AP.htm

GAPDH (ABclonal, AC002), https://abclonal.com.cn/catalog/AC002
GAPDH (Proteintech, 60004-1-Ig), https://www.ptgcn.com/products/GAPDH-Antibody-60004-1-Ig.htm
PCNA (Proteintech, 10205-2), https://www.ptgcn.com/products/PCNA-Antibody-10205-2-AP.htm
PCNA (Proteintech, 60097-1 -Ig), https://www.ptgcn.com/products/PCNA-Antibody-10205-2-AP.htm
Cyclin A2 (Proteintech, 18202-1-AP), https://www.ptgcn.com/products/CCNA2-Antibody-18202-1-AP.htm
Cyclin D1 (ABclonal, A0310), https://abclonal.com.cn/catalog/A0310
HDAC1 (Abcam, ab7028), https://www.abcam.cn/hdac1-antibody-ab7028.html
HDAC2 (Abcam, ab12169), https://www.abcam.cn/hdac2-antibody-hdac2-62-ab12169.html
HDAC1 (Cell Signaling Technology, 34589S), https://www.cellsignal.cn/products/primary-antibodies/hdac1-d5c6u-xp-rabbit-mab/34589
HDAC2 (Cell Signaling Technology, 57156S), https://www.cellsignal.cn/products/primary-antibodies/hdac2-d6s5p-rabbit-mab/57156
FLAG (Sigma, F7425), https://www.sigmaaldrich.cn/CN/zh/product/sigma/f7425
puromycin (Millipore, MABE341), https://www.sigmaaldrich.cn/CN/zh/product/mm/mabe341
EIF2S1 (ABclonal, A0764), https://abclonal.com.cn/catalog/A0764
p-EIF2S1-S51 (ABclonal, AP0745), https://abclonal.com.cn/catalog/AP0745
RPS6 (ABclonal, A6058), https://abclonal.com.cn/catalog/A6058
p-RPS-S240/S242 (ABclonal, AP0537), https://abclonal.com.cn/catalog/AP0537
HSL (ABclonal, A15686), https://abclonal.com.cn/catalog/A15686
p-HSL (ABclonal, AP1242), https://abclonal.com.cn/catalog/AP1242
HSP90 (Cell Signaling Technology, 4874S), https://www.cellsignal.cn/products/primary-antibodies/hsp90-antibody/4874?site-search-type=Products&N=4294956287&Ntt=4874s&fromPage=plp&_requestid=3314927
Albumin (R&D systems, MAB1455), https://www.rndsystems.com/cn/products/human-serum-albumin-antibody-188835_mab1455
IRDye 680RD Donkey anti-Mouse (LI-COR Biosciences, 926-68072), https://www.licor.com/bio/reagents/irdye-680rd-donkey-anti-mouse-igg-secondary-antibody
IRDye 800CW Donkey anti-rabbit (LI-COR Biosciences, 925-32213), https://www.licor.com/bio/reagents/irdye-800cw-donkey-anti-rabbit-igg-secondary-antibody
Alexa 647 goat-anti-mouse (Invitrogen, A31571), https://www.thermofisher.cn/cn/zh/antibody/product/Goat-anti-Mouse-IgG-H-L-Cross-Adsorbed-Secondary-Antibody-Polyclonal/A-21235
Alexa 488 donkey-anti-rabbit (Invitrogen, R37118),https://www.thermofisher.cn/cn/zh/antibody/product/Donkey-anti-Rabbit-IgG-H-L-Secondary-Antibody-Polyclonal/R37118

Eukaryotic cell lines

Policy information about cell lines and Sex and Gender in Research

Cell line source(s)	HEK293T cell lines were obtained from Cell Bank, Type Culture Collection Committee, Chinese Academy of Sciences, Shanghai.<br>The HUES9 human embryonic stem cells used in this study was kindly provided by the department of stem cell and regenerative biology in Harvard University and Harvard stem cell institute.
Authentication	None of the cell lines used have been authenicationed.
Mycoplasma contamination	All cells used in this study were found negative for mycoplasma contamination.
Commonly misidentified lines (See ICLAC register)	No commonly misidentified cell lines were used.

Animals and other research organisms

Policy information about studies involving animals; ARRIVE guidelines recommended for reporting animal research, and Sex and Gender in Research

Laboratory animals

Animal maintenance:
All animals were maintained and used in accordance with the guidelines of, and under approval by, the Institutional Animal Care and Use Committee of the Shanghai Institute for Nutrition and Health. All animals presented a healthy status and male mice were employed for all experiments. The light was on from 7 a.m. to 7 p.m., with the temperature kept at 21-24 °C and humidity at 40-70%. All Fah-/- animals were kept on the drug NTBC at 19.2 mg per liter drinking water to prevent liver failure. To determine the necessary titers for lentivirus delivery, 8-week-old male Fah-/- Cas9LSL+/+ animals were administrated with lentiviral vectors at different titers by tail vein injection. Animals were subjected to PHx surgery for liver regeneration analysis at around 8-11 weeks' old.For liver regeneration analysis in chronic high-fat diet-fed mice, 6-week-old Cas9LSL+/+ mice were fed with normal chow diet (NCD) (Shanghai Laboratory Animal Center, P1103F) or high-fat diet (HFD) (60% of energy from fat; Research Diets, D12492) for 8 weeks to induce hepatic steatosis. After 5 weeks on HFD, AAV8 vectors were administered by tail vein injection to deplete MIER1 in hepatocytes. For liver regeneration analysis in aging mice, about one-year-old Cas9LSL+/+ mice were administered with AAV8 vectors expression Cre recombinase and Mier1 sgRNA to deplete MIER1 in liver cells. For liver regeneration analysis in acute high fat diet-fed mice, 8-week-old Cas9LSL+/+ mice were fed with high-fat diet (60% of energy from fat; Research Diets, D12492) 2 hours before hepatectomy, with diet control mice fed with normal chow diet (NCD) (Shanghai Laboratory Animal Center, P1103F). Animals were subjected to partial hepatectomy (PHx) surgery for analysis 3 weeks after viral delivery. For liver regeneration analysis in sugar-treated animals, 8-week-old C57BL/6J animals were fed with NCD and high sugar water containing 23.1 g/L fructose and 18.9 g/L d-glucose 2 hours before hepatectomy, with control mice fed with NCD and normal water. For liver regeneration analysis in ISRIB treated mice, 8-week-old C57BL/6J mice were injected with ISRIB (Selleck, S0706) at a dose of 5 mg/kg by intraperitoneal injection three times per day after hepatectomy until sacrificing for analysis. Animals injected with equal volume of DMSO (YEASEN, 60313ES60) were used as control. For sham surgery, animals were subjected to midline laparotomy only with no liver section. For hepatic MIER1 overexpression, 8 weeks' old male C57BL/6J mice were administered with AAV8 vectors expressing MIER1-FLAG per mouse for 3 weeks before PHx.

	Animal source:<br>Fah-/- mice were provided by Dr. Xin Wang.<br>The colony of Cre-dependent Cas9 knockin mouse (Jackson laboratory) was maintained by crossing with wild-type C57BL/6 mice (Shanghai Laboratory Animal Co.Ltd, China).<br>Lipe loxp/loxp mice were generated by a homologous recombination and classic embryonic stem cell targeting strategy (Shanghai Biomodel Organism Science & Technology Development Co.,Ltd). Loxp sequences were inserted flanking the second exon. Adipose tissue-specific Lipe knockout mice were generated by crossing Lipe loxp/loxp mice with Adiponectin-Cre mice (Jackson Laboratory). Lipe loxp/loxp mice were then bred with Lipe loxp/loxp mice: Cre/+ animals to generate Lipeloxp/loxp mice (used as control) and Lipe loxp/loxp: Cre/+ mice (Lipe-AKO) as adipose-specific knockout animals. To further deplete MIER1 in the Lipe-AKO animals, Cas9 LSL+/+ mice were bred with the Lipe-AKO mice to generate the Lipe-AKO; Cas9 LSL+/- animals.
Wild animals	No wild animals were involved in this study.
Reporting on sex	All mice used in this study were male
Field-collected samples	This study did not involved samples collected from field.
Ethics oversight	All animals were maintained and used in accordance with the guidelines of, and under approval by, the Institutional Animal Care and Use Committee of the Shanghai Institute for Nutrition and Health (ethical committee approval no. SINH-2020-DQR3).

Note that full information on the approval of the study protocol must also be provided in the manuscript.

ChIP-seq

Data deposition

☒ Confirm that both raw and final processed data have been deposited in a public database such as GEO.

☒ Confirm that you have deposited or provided access to graph files (e.g. BED files) for the called peaks.

Data access links<br>*May remain private before publication.*	The accession numbers for the MIER1-FLAG ChIP-seq data reported in this paper is GSE188747 (Token: whsrqsogjnghbar. Website: https://www.ncbi.nlm.nih.gov/geo/query/acc.cgi?acc= GSE188747). The accession numbers for the H3K27ac ChIP-seq data reported in this paper is GSE188742 (Token: oxwluacgpjqrtmn. Website: https://www.ncbi.nlm.nih.gov/geo/query/acc.cgi?acc= GSE188742).
Files in database submission	Raw and processed data
Genome browser session<br>(e.g. UCSC)	No longer applicable

Methodology

Replicates	ChIP-seq experiment was performed from 3 biological replicates of animals in each group.
Sequencing depth	The 75-nt sequence reads generated by Illumina sequencing (using NextSeq 500).
Antibodies	FLAG M2 agarose (Sigma, A2220)<br>H3K27ac (Active Motif, 39133)
Peak calling parameters	Peaks were called using the MACS algorithms (MACS 2.1.0) with an irreproducible discovery rate (IDR) of <0.05. Peak filtering was performed by removing false ChIP-Seq peaks as defined with the ENCODE blacklist.
Data quality	Only reads that pass Illumina's purity filter, align with no more than 2 mismatches, and map uniquely to the genome were used in the subsequent analysis. Duplicate reads ("PCR duplicates") are removed, unless stated otherwise.
Software	ChIPseeker v1.18.0<br>bedtools v2.25.0

Flow Cytometry

Plots

Confirm that:

☒ The axis labels state the marker and fluorochrome used (e.g. CD4-FITC).

☒ The axis scales are clearly visible. Include numbers along axes only for bottom left plot of group (a 'group' is an analysis of identical markers).

☒ All plots are contour plots with outliers or pseudocolor plots.

☒ A numerical value for number of cells or percentage (with statistics) is provided.

Methodology

Sample preparation	Primary hepatocytes were collected from mice injected with different titers of lentivirus.
Instrument	BD FACSAria II flow cytometer
Software	BD FACSDiva 8.0.2, FlowJo 10.0.7
Cell population abundance	Abundance of cell populations have been provided in figures.
Gating strategy	Starting cell population was determined by FSC-A/SSC-A. The FSC-A/FSC-H gating strategy was used to select single cells.

☒ Tick this box to confirm that a figure exemplifying the gating strategy is provided in the Supplementary Information.
