# Supplementary material for: Acute liver steatosis translationally controls the epigenetic regulator MIER1 to promote liver regeneration in a study with male mice
Source: Nat Commun. 2023 Mar 18;14:1521. doi: 10.1038/s41467-023-37247-9 (PMC10024732; doi:10.1038/s41467-023-37247-9)
Supplement: Supplementary file 2 — Peer Review File [file 41467_2023_37247_MOESM2_ESM.pdf]

nature portfolio

Peer Review File

Acute liver steatosis translationally controls the epigenetic regulator MIER1 to promote liver regeneration

Open Access This file is licensed under a Creative Commons Attribution 4.0 International License, which permits use, sharing, adaptation, distribution and reproduction in any medium or format, as long as you give appropriate credit to the original author(s) and the source, provide a link to the Creative Commons license, and indicate if changes were made. In the cases where the authors are anonymous, such as is the case for the reports of anonymous peer reviewers, author attribution should be to 'Anonymous Referee' followed by a clear attribution to the source work. The images or other third party material in this file are included in the article's Creative Commons license, unless indicated otherwise in a credit line to the material. If material is not included in the article's Creative Commons license and your intended use is not permitted by statutory regulation or exceeds the permitted use, you will need to obtain permission directly from the copyright holder. To view a copy of this license, visit http://creativecommons.org/licenses/by/4.0/.

REVIEWER COMMENTS</B>

Reviewer #1 (Remarks to the Author):

In the manuscript "The transient liver steatosis translationally controls the epigenetic regulator MIER1 to promote liver regeneration" the authors present an intriguing mechanism by which the acute liver steatosis in liver regeneration functions as an important signal via translationally control of MIER1 to modulate chromatin status and promote liver regeneration, and explains why the lipid accumulation and regeneration couples in healthy livers but uncouples in fatty livers. In general, it is a very interesting work with a clear and interesting mechanism. But some minor points should be better discussed:

1. The screening system is well-designed and also clearly described. For in vivo screening, the infection rate is 2% by injecting 1e9 vectors to mice. The authors should further explain the reason for choosing these experimental conditions for better readability.
2. According to the conclusion from this manuscript, MIER1 functions as a regulator for cell cycle and cell proliferation, which is derived from its significant role in the regulation of cell cycle genes. It is understandable that hepatocyte-specific MIER1 depletion shows completely no effect on cell cycle genes in the time 0 group because cyclin D is not required and is generally suppressed on the normal condition (Figure 1F). However, it is still deserved to have a try using cultured hepatocytes or other proliferation cells to test the effects of MIER1 depletion.
3. Immunoblots in liver tissues after hepatectomy showed that MIER1 protein levels did not change significantly at 24h based on statistic analysis (Actually very clear change in left panel figure 3A). However, as shown in figure1F, the cyclin D level dramatically increased at 24h, which suggests there might be other important mechanisms for the regulation of cyclin D. Please provide a further discussion on it.
4. There's one minor issue needed to be addressed. There is a recent paper (PMID: 35090595), which is highly related to your study. The authors should discuss this paper and point out the difference and advantages in the discussion.
5. The authors need to at least discuss the reason why MIER1 could not be downregulated after PHx in crHFD and aging mouse models.

Reviewer #2 (Remarks to the Author):

The manuscript by Chen et al describes a novel finding that lipid accumulation in hepatocytes during the early stage of liver regeneration promotes cell cycle gene activation and regeneration via downregulation of MIER1. This study starts with a screen for epigenetic genes serving as negative regulators of liver regeneration, and find that downreglation of MIER1 allows the liver to repopulate better following injury and get bigger, faster, following partial hepatectomy. Part of this effect is attributed to MIER1 recruitment of HDAC1/2, however, that point is the least robust part of the story. The most interesting and novel finding is the inference that lipid influx into hepatocytes in the early

stages of regeneration from peripheral stores promotes regeneration by downregulating MIER1. The authors go on to show that providing a high fat diet for a short time before PH enhances liver regeneration, while chronic high fat diet reduces regenerative capacity and link this finding to MIER1 regulation. One set of experiments also implicates MIER1 in aging related regeneration deficiency.

Overall, this is a highly interesting finding and the work, as presented, is solid. It provides new insight into a mechanism by which steatosis impacts regeneration and for the first time, ties lipid redistrubtion from adipocytes to a mechanistic change in hepatocytes that makes them better at proliferating.

There are a few major points that, if addressed, could significantly enhance these findings. Most importantly are addressing the mechanism by which lipids impact the stabilization of MIER1 protein and clarifying whether HDAC1 is removed or chromatin opening is restricted at cell cycle genes in regeneration deficient animals. Moreover, some of their data is contradictory to other findings (Zhang et al 2021) which show that the cell cycle genes are in an open chromatin configuration in the liver, and this implies that histone acetylation is present on these genes, whereas this current study implies that MIER1 recruitment of HDAC1/2 in quiescent livers keeps those genes silenced. There are also some minor points that should be tended to.

Major:

1. The finding that MIER1 binds HDAC1 would imply that HDAC1 recruitment is to cell cycle genes is dependent on MIER1. This needs to be demonstrated.
2. Other investigators have shown that the chromatin is open and accessible on cell cycle regulatory genes in quiescent livers, however, the current study implies that these genes are made accessible by the MIER1 mediated removal of histone acetylation. These findings are not in agreement, and raises questions such as: Does the MIER1 occupancy pattern overlap with regions of open chromatin? Does HDAC1 or HDAC2 occupancy change in MIER1 deficient hepatocytes prior too partial hepatectomy? this
3. What is the long term consequence of MIER1 deficeincy? Do the livers keep growing? Do they stop growing and stay large? Late time points after PH would be useful to examine (at least a week or a month).
4. If the liver cannot adequately regenerate, survival can be impacted. Does the overexpression of MIER1 (Figure 3) or reduction in lipid redistrution (figure 5) decrease survival? Any other hepatic-function related outcomes (clotting, ascites etc)?
5. The implication that MIER1 is translationally regulated during regeneration is interesting, and that data is clear (Figure 4). However, it is not clear that this is due to a stress response, as the data linking EIF2S1 to this translational finding are not entirely supported. Moreover, although a semantic point, referring too the process of restoring homeostasis in the liver as an ER stress response may not be accurate. For instance, there is no indication in the current study that the translational regulation of MIER1 is ER stress related. Some experiments to address this could be performed or the text should be altered to reflect other possibilities.
6. One of the most striking findings in this study is presented in Figure 5. This finding would be significantly strengthened if the authors were too tie this finding back to the epigenetic regulation of the cell cycle genes that they propose MIER1 to regulate (i.e. genes identified through ChIPseq).

7. The aging study is very interesting. Is MIER1 occupancy different in aged animals? Does the cell cycle gene expression pattern restored in the aged animals where MIER1 is depleted? Similar questions for the mice on a chronic HFD.

Minor

1. The screen is described as focused on epigenetic regulators, but many of the genes on the list that were screened do not serve this function directly – for instance, there are lots of signaling molecules, metabolic enzymes, toxicant response factors and others that are, at best, distantly and indirectly associated with chromatin. There are also important epigenetic regulators that are not on the list. More accurate descrition of the pool of genes screened is needed.
2. H3k27ac is a mark of enhancers and it is not clear why is the H3k27ac enrichment at promotors is investigated (Figure 2F-G).
3. Many of the graphs lack information under each bar or legends are attached to a different panel (example Figue 1E, 7A, 7D, 7I). Labeling on all figures should be checked and corrected.
4. There are a number of grammatical or usage issues that need to be cleaned up (i.e. “the steatosis” is used throughout instead of “steatosis”)
5. Changing the use of words like “role for” or regulates without implying what the specific role or regulation would make the paper easier to read.
6. Figure 4A is very hard to interpret. It would be better to change the way this data is presented.

Reviewer #3 (Remarks to the Author):

The manuscript by Chen and Chen et al identified a new role for protein MIER1 in hepatic regeneration. The manuscript uses a nice in vivo CRISPR screening system and a Fah-/- mice to identify new regulators in liver regeneration. Depletion of Mier1 was discovered to improve liver regeneration. Using genetic, molecular and biochemical tools, the study suggests that acute steatosis impairs MIER1 translation, which in turn changes chromatin accessibility to promote transcription of cell cycle genes and liver regeneration. Furthermore, this regulatory process was impaired in murine models of aging and chronic steatosis. This is an interesting study that has novelty. However, there are concerns involving experimental rigor and interpretation. The rigor of the MIER1 translation conclusion is not convincing and there are figures of insufficient quality. Another concern is that the study does not address these results to the human liver. No primary or immortalized human hepatocytes were studied in the context of the MIER1 mechanism. Addressing these concerns is important to support the stated conclusions.

Major concerns:

1. The key findings of this manuscript need to be assessed in human hepatocytes/tissues to address the potential translation of this work to human liver transplant/regeneration.
2. The manuscript should measure/show steatosis in the tissues to confirm their model. Furthermore, the manuscript indicates that mice received HFD for 8-9 weeks in the text of the results, but the rest of the manuscript indicates 8 weeks. Please clarify.
3. The levels of cyclin D1 increases with MIER1 loss in figure 1, but also increased with MIER1 overexpression, and shows no change with MIER1 KO in the HFD model. How to justify the inconsistent changes in cyclin D1 in the manuscript?
4. mRNA is translated, protein is synthesized. The explanation for looking at eIF2S1 (eIF2alpha) phosphorylation should be clearer. Saturated free fatty acids have been reported to induce ER stress, trigger eIF2 phosphorylation (PERK eIF2) kinase which broadly lowers translation initiation. The experiments in Fig. 4H, especially the Mer1 mRNA placement in in sub (difficult to see monosomes or free ribomses vs polysomes were not convincing and lacked controls (other mRNAs known to be differentially regulated by the UPR/ISR, such as does ATF4 shift towards suggested polysomes). Overall, the conclusion the conclusion that there is dynamic change in translational, contributing to MIER1 redfuction, was not sufficiently supported by the presented results.
5. There are also some concerns about the rigor of polysomes and measured mRNAs in fractions for assessing translation repression of MIER1 in Figure 5.
6. Quantification of western blots is needed in several figure panels (Figure 1F, Figure 3C, Figure 4C, Figure 5A, D, I, Figure 6F, Figure 7B, C, G and H, Figure S2I, Figure S7, Figure S8B, C, E, F, I), The quantitation is needed to support key conclusions and confirm consistency of biological replicates for experiments where only 2 replicates are shown in the WB. Some blots appear overexposed and/or of poor quality (Figure 4F, Figure 5A, Figure 6F PCNA, Figure 8H, EWIF2S1; Figure S8E)

Minor concerns:

1. Portions of the abstract are not clear. Factors and pathways, EID2S and Lipe knockdown are referred to without sufficient introduction. Furthermore, the subjects and meaning of some sentences are not fully clear. It would be helpful to simplify some sentences.
2. The title for Supplementary Figure 9 indicates a comparison between chronic and acute steatosis, but the names of the groups in comparison to the title is confusing. Provide better descriptions to improve clarity.
3. Several graphs appear to be missing labels, or the legends are in awkward locations and not near the graphs themselves. This hampers the clarity of the manuscript.

Figure 1E
Figure 3D and F
Figure 5D, F, G, I
Figure 6D and E
Figure 7A, D, I

Reviewer #4 (Remarks to the Author):

Chen and colleagues have conducted a series of well thought out and logical studies that have defined a novel role for Mier1 in regulating liver regeneration. There are a number of strengths to this paper including the use of an in vivo screen for factors that regulate liver regeneration, a mix of in vivo and in vitro studies with sophisticated molecular approaches, and the overall conceptual novelty of the work. However, there are some things to be improved.

The studies focus a great deal on the transient steatosis that occurs with liver resection in mice. However, it is less clear that this is relevant in humans for a variety of reasons. The severity of the partial hepatectomy likely causes anorexia in the mice and even acute fasting in mice can drive steatosis. In humans, caloric restriction for the same duration would lead to reduced liver fat and only after several days would liver fat accumulation manifest.

There are also many questions about the lipid accumulation in these mice.
1. Can the authors determine why adipose tissue lipolysis is increased (humoral, neuroendocrine, food intake)?
2. Is the phosphorylation of HSL and PLIN1 increased after PH?
3. Plasma free fatty acids and glycerol should be measured after PH and in the HSL KO mice.

Recent work has defined the zonal subpopulations of hepatocytes that form the major regenerative pools. Is Mier1 expressed uniformly across all zones of the liver? Although liver fat is measured by biochemical assays, there is no data on the spatial distribution of the fat. Is this zonal as well? Is there a negative correlation between Mier1 expressing cells and lipid accumulation?

There are some methodologic issues that need to be addressed.
1. A normal chow is not the appropriate control for the 60% fat diet. The HFD is made up of defined components and also has added sucrose. Normal chow is not made of refined components and has no added sugar. A matched low fat control chow needs to be used and the effect of added sugar also needs to be addressed. Without this important control, the conclusions from the HFD studies are not supported. These studies need to be re-done with this important control.
2. For the in vitro studies with fatty acids, I could not find specified what the concentration of fatty acids was in these studies. Please specify. Also, the saturated fats were found to have effects on Mier1 and translational control. Care must be taken in interpreting these results as high levels of sat fat are toxic. It's also not clear that high levels of saturated fatty acids are physiologic, since in vivo the liver is exposed to both saturated and unsaturated fatty acids.
3. There were no sham surgery animals included in these studies. Does hepatic steatosis occur with laparotomy but no PH? This important control should also be included.

The effect of the acute HFD feeding on liver TG is very modest. Moreover, the HFD did pre load the liver with lipid (as was the stated intention) since there was no increase in TG at time 0. It is not clear exactly what is happening in this experiment since the HFD also has added sucrose compared to the control

chow. Food intake after PH should also be assessed.

Minor:

Overall the manuscript is very well written. However the sentence beginning on line 72 and running through line 77 is very long and doesn’t completely make sense.

Line 71. Technically, “energy” is not produced. The amount of energy in the universe remains constant.

We are truly grateful, and wish to first express our sincere acknowledgements to the reviewers and the editor for the time and the effort that have been spent in reviewing our manuscript. We appreciate for the thorough and helpful comments. Please find the detailed responses below. We have where the revised results presented included after each response, with also results from newly-performed experiments displayed immediately below for easy access.

REVIEWER COMMENTS

Reviewer #1 (Remarks to the Author):

In the manuscript "The transient liver steatosis translationally controls the epigenetic regulator MIER1 to promote liver regeneration" the authors present an intriguing mechanism by which the acute liver steatosis in liver regeneration functions as an important signal via translationally control of MIER1 to modulate chromatin status and promote liver regeneration, and explains why the lipid accumulation and regeneration couples in healthy livers but uncouples in fatty livers. In general, it is a very interesting work with a clear and interesting mechanism. But some minor points should be better discussed:

1. The screening system is well-designed and also clearly described. For in vivo screening, the infection rate is 2% by injecting 1e9 vectors to mice. The authors should further explain the reason for choosing these experimental conditions for better readability.

Response: Many thanks for the comments. The reason we chose 1e9 vectors for injection per animal, which resulted in around 2% in vivo infection rate is mainly because we wanted the majority of cells to be infected by one virus thus one sgRNA and one gene knocked down in the screening system. We included this explanation in the revised manuscript.

2. According to the conclusion from this manuscript, MIER1 functions as a regulator for cell cycle and cell proliferation, which is derived from its significant role in the

regulation of cell cycle genes. It is understandable that hepatocyte-specific MIER1 depletion shows completely no effect on cell cycle genes in the time 0 group because cyclin D is not required and is generally suppressed on the normal condition (Figure 1F). However, it is still deserved to have a try using cultured hepatocytes or other proliferation cells to test the effects of MIER1 depletion.

Response: Thanks for this suggestion. In order to test the function of MIER1 in an *in vitro* system, we generated human liver organoids derived from human pluripotent stem cells (hPSCs), following a previously established protocol, which can partly recapitulate the organogenesis events during liver regeneration. Interestingly, when *MIER1* was knocked down using the lenti-Cas9/CRISPR system in hPSCs, the size of liver organoids is clearly larger than wild-type liver organoids (**revised Figure S3A**). In addition, expression levels of several cell cycle markers, including Cyclin A2, Cyclin D1, PCNA are significantly enhanced in liver organoids after MIER1 depletion (**revised Figure S3B**), suggesting a potential similar function of MIER1 in promoting human liver cell proliferation. No obvious difference was observed in hepatocyte markers, indicating that MIER1 depletion does not affect the liver characteristics (**revised Figure S3C**). Altogether, these data suggested that MIER1 depletion also promoted liver cell proliferation in hPSC-derived liver organoids.

Revised Figure S3

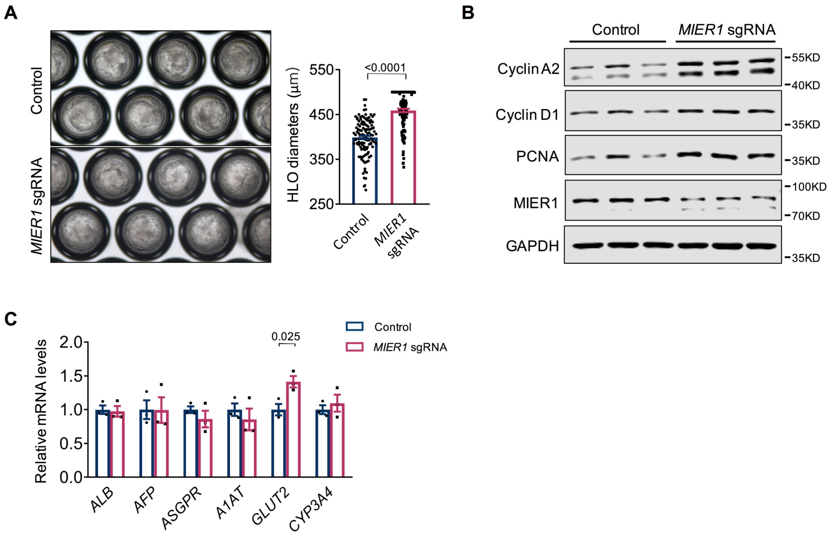

3. Immunoblots in liver tissues after hepatectomy showed that MIER1 protein levels did not change significantly at 24h based on statistic analysis (Actually very clear change in left panel figure 3A). However, as shown in figure1F, the cyclin D level dramatically increased at 24h, which suggests there might be other important mechanisms for the regulation of cyclin D. Please provide a further discussion on it.

Response: Thanks for pointing this out. The level of MIER1 protein did show a decrease trend in 24h after liver hepatectomy. Regarding the change of cyclin D, there are indeed many other mechanisms for its regulation, e.g the cytokine and growth factor-mediated pathways activated in early liver regeneration. We propose MIER1 here as an epigenetic "*break*" for cyclin D1 expression and cell cycle progression. During normal liver regeneration, the physiological downregulation of MIER1 in 24h-36h further promoted cyclin D1 expression and cell proliferation. When MIER1 was depleted in liver in our system, the earlier release of the "*break*" brought forward the cyclin D1 expression, which is why the cyclin D1 expression was considerably higher in 24h post Phx after MIER1 depletion.

4. There's one minor issue needed to be addressed. There is a recent paper (PMID: 35090595), which is highly related to your study. The authors should discuss this paper and point out the difference and advantages in the discussion.

Response: Many thanks for the suggestion. We included discussion of this recent paper together with the other two papers, which all adopted genetic screening for new regulators in liver regeneration in the revised manuscript.

"Before us, a couple of studies have been performed with *in vivo* screenings for potential regulators in liver regeneration. For example, one was published at as early as 2013[49], with in total 631 shRNAs targeting genes that were found in focal genomic deletions of human hepatocellular carcinomas, which successfully identified MKK4 as a negative regulator in liver regeneration; another one was published in 2019[50], focusing on 147 mutant genes identified from diseased liver samples with a pool of 882

sgRNAs, which also successfully validated several regulators in liver regeneration; and a recent study used both pooled CRISPR knockout and activation screening systems, targeting primarily 165 chromatin regulatory proteins, and identified BAZ2 as a druggable regulator in liver regeneration[51]. All these studies have beautifully demonstrated the power of high-throughput screenings in identifying regulators in liver regeneration. Differently from the above-mentioned studies, which have adopted the transposon system to incorporate shRNAs or sgRNAs to liver genome, we here used lentiviral system for *in vivo* sgRNA integration, and focused on over 1500 genes with over 9000 sgRNAs, presenting so far the largest library used for *in vivo* liver regeneration screening."

5. The authors need to at least discuss the reason why MIER1 could not be downregulated after PHx in crHFD and aging mouse models.

Response: Thanks for the suggestion. We included the discussion on the compromised MIER1 regulation during liver regeneration in crHFD and aging animals in revised manuscript. We suspect that the different liver lipid content in the crHFD and aging mouse models, as compared to the acute steatosis in healthy animals during liver regeneration, is one of the primary reasons. In order to have a detailed understanding of the lipid content differences between the chronic liver steatosis in crHFD and aging animals and the acute steatosis during liver regeneration in young and healthy animals, we performed lipidomic analysis in these animals before or 24h after PHx (**Revised Figure S12**). The lipidomic analyses demonstrated remarkable differences in lipid content between the chronic steatosis after 8 weeks' HFD and the acute steatosis in normal liver regeneration. For example, lipids in chronic steatosis contain clearly more cholesteryl ester (CE), diacylglycerol (DAG), but less phosphatidylethanolamine (PE), phosphatidylcholine (PC) and lyso-PE (LPE) (**Revised Figure S12, Supplementary Data 3**). In addition, the relative changes in lipid levels between 0 h and 24 h after surgery in crHFD animals are considerably less than ones in NCD animals (**Revised Figure S12, Supplementary Data 3, 4**). We therefore suspect that liver tissues with

chronic steatosis have already been well-adapted with high liver lipid content, thus become insensitive to the additional stress posed by the transient liver steatosis in early regeneration, although further investigation is needed to depict the detailed molecular mechanism underlying this phenomenon.

Reviewer #2 (Remarks to the Author):

The manuscript by Chen et al describes a novel finding that lipid accumulation in hepatocytes during the early stage of liver regeneration promotes cell cycle gene activation and regeneration via downregulation of MIER1. This study starts with a screen for epigenetic genes serving as negative regulators of liver regeneration, and find that downreglation of MIER1 allows the liver to repopulate better following injury and get bigger, faster, following partial hepatectomy. Part of this effect is attributed to MIER1 recruitment of HDAC1/2, however, that point is the least robust part of the story. The most interesting and novel finding is the inference that lipid influx into hepatocytes in the early stages of regeneration from peripheral stores promotes regeneration by downregulating MIER1. The authors go on to show that providing a high fat diet for a short time before PH enhances liver regeneration, while chronic high fat diet reduces regenerative capacity and link this finding to MIER1 regulation. One set of experiments also implicates MIER1 in aging related regeneration deficiency.

Overall, this is a highly interesting finding and the work, as presented, is solid. It provides new insight into a mechanism by which steatosis impacts regeneration and for the first time, ties lipid redistribution from adipocytes to a mechanistic change in hepatocytes that makes them better at proliferating.

There are a few major points that, if addressed, could significantly enhance these findings. Most importantly are addressing the mechanism by which lipids impact the stabilization of MIER1 protein and clarifying whether HDAC1 is removed or chromatin opening is restricted at cell cycle genes in regeneration deficient animals. Moreover, some of their data is contradictory to other findings (Zhang et al 2021) which show that the cell cycle genes are in an open chromatin configuration in the liver, and this implies

that histone acetylation is present on these genes, whereas this current study implies that MIER1 recruitment of HDAC1/2 in quiescent livers keeps those genes silenced. There are also some minor points that should be tended to.

Response: We appreciate very much the encouragements from this reviewer, and also the insightful points raised. To address these issues, we performed additional experiments, which are presented below specifically to each comment.

Major:

1. The finding that MIER1 binds HDAC1 would imply that HDAC1 recruitment is to cell cycle genes is dependent on MIER1. This needs to be demonstrated.

Response: Many thanks for the suggestion. To address this point, we did CHIP-qPCR of HDAC1 and HDAC2 at 24h after PHx in wild-type and *Mier1*-sgRNA-treated livers, at gene loci bound by MIER1, including *Ccne2, Ccpg1* and *Cdc25a*. The results showed minimal changes between the two groups, suggesting that the genomic recruitment of HDAC1 and HDAC2 to cell cycle genes is not dependent on MIER1 (**Revised Figure S4G**). The chromatin regulatory mechanism by MIER1 during liver regeneration warrants future investigation. Although the current investigations into the potential involvement of HDAC1/HDAC2 in MIER1 regulation presented negative results, we still decided to include this piece of data in supplemental information, which may still be helpful to the future investigation of MIER1 function.

Revised Figure S4

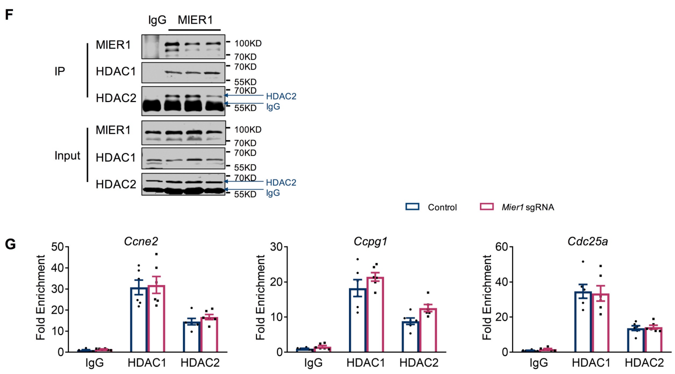

2. Other investigators have shown that the chromatin is open and accessible on cell cycle regulatory genes in quiescent livers, however, the current study implies that these genes are made accessible by the MIER1 mediated removal of histone acetylation. These findings are not in agreement, and raises questions such as: Does the MIER1 occupancy pattern overlap with regions of open chromatin? Does HDAC1 or HDAC2 occupancy change in MIER1 deficient hepatocytes prior too partial hepatectomy?

Response: We appreciate the comments, and apologize for presenting the data about HDAC1/2 with no firm demonstration about the functional relationship between HDAC1/2 and MIER1. As presented in the response to the above comment, the current finding suggested that although MIER1 interacts with HDAC1/2, the genomic recruitment of HDAC1 and HDAC2 to cell cycle genes is not dependent on MIER1.

To directly assess the status of chromatin accessibility on cell cycle regulatory genes in quiescent livers and livers after PHx, and the possible regulations by MIER1, we further performed ATAC-seq in livers before and after PHx, and with or without MIER1 depletion. The ATAC-seq analysis showed the following findings:

1) In total 17556 peaks were identified with ATAC-seq in quiescent livers before partial hepatectomy, in which 3661 peaks were also bound by MIER1 (**Revised Figure S4A**). GO analysis of genes near these 3661 peaks showed enrichment in transcriptional regulation, cell cycle, etc (**Revised Figure S4A**). This ATAC-seq result is consistent with the previous discovery (Zhang et al 2021), showing that even not in active transcription, cell cycle genes reside in open chromatin in quiescent liver, which are bound by MIER1.

2) Further ATAC-seq analysis of liver tissues at 24 h after partial hepatectomy identified 10801 peaks that showed higher intensity in comparison to ones in quiescent liver, suggesting increased chromatin accessibility near these genomic loci in early liver regeneration (**revised Figure 2D, S4B**). Of these 10801 peaks, 2221 peaks were bound by MIER1, which were close to cell cycle-relevant genes (**revised Figure 2D**).

Interestingly, although liver MIER1 depletion did not change the chromatin accessibility of these genomic loci in quiescent livers, MIER1 depletion resulted in a significant increase in their chromatin accessibility at 24 h after partial hepatectomy (**Revised Figure 2E, 2F**). These results were consistent with the RNAseq analysis, suggesting that liver MIER1 depletion increased the chromatin accessibility and transcription of cell cycle-relevant genes during liver regeneration, but not in quiescent livers.

Revised Figure S4

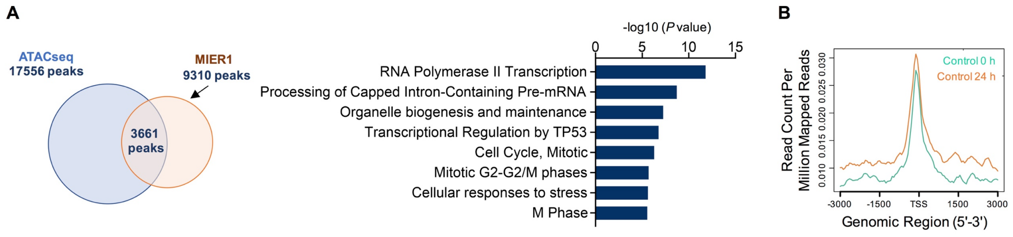

Revised Figure 2

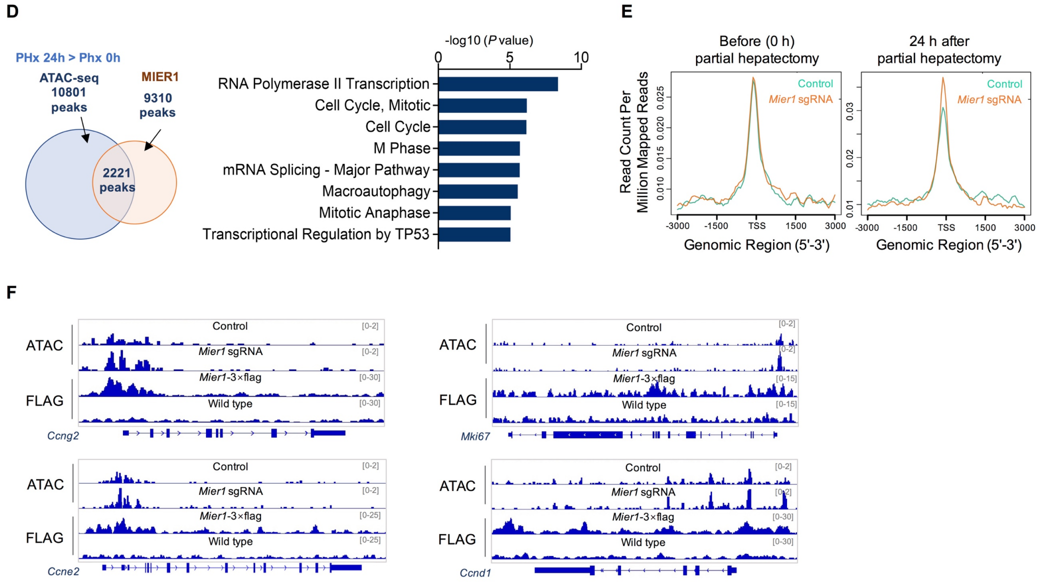

3. What is the long-term consequence of MIER1 deficiency? Do the livers keep growing? Do they stop growing and stay large? Late time points after PH would be useful to examine (at least a week or a month).

Response: We appreciate the suggestion. In the revised manuscript, we added analyses at 12 days, and 2 months post PHx. The results showed no significant difference

between two groups at 12 days or 2 months after PHx, and no tumor formation were observed in *Mier1-/-* livers up to 2 months after PHx (**Revised Figure S2D-E**). These results indicate that MIER1 liver depletion leads to enhanced proliferation during the liver regeneration, without disturbing the reestablishment of the homeostasis.

Revised Figure S2

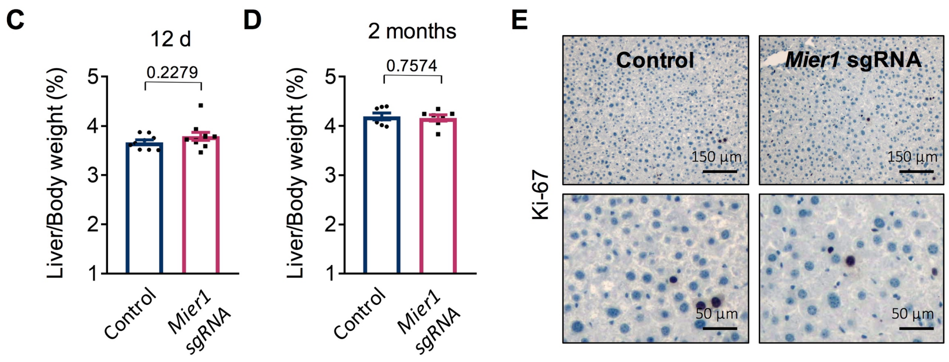

4. If the liver cannot adequately regenerate, survival can be impacted. Does the overexpression of MIER1 (Figure 3) or reduction in lipid redistrution (figure 5) decrease survival? Any other hepatic-function related outcomes (clotting, ascites etc)?

Response: Thanks for raising the point. We in general did not observe any severe effect to animal survival or clotting, ascites, etc, in animals with MIER1 overexpression or adipose-specific knockout of *Lipe*. Based on our observations, we propose that MIER1 acts as a sort of epigenetic "*brake*" during liver regeneration, which normally represses cell cycle gene expression; upon the start of liver regeneration, decreased MIER1 protein releases the "*brake*", resulting in increased cell cycle gene expression, and cell proliferation. Therefore, MIER1 overexpression and reduction in lipid redistribution after *Lipe* knockout only delay liver regeneration, due the existence of the MIER1 *"brake"*, but do not entirely disrupt the regeneration.

5. The implication that MIER1 is translationally regulated during regeneration is interesting, and that data is clear (Figure 4). However, it is not clear that this is due to a stress response, as the data linking EIF2S1 to this translational finding are not entirely

supported. Moreover, although a semantic point, referring too the process of restoring homeostasis in the liver as an ER stress response may not be accurate. For instance, there is no indication in the current study that the translational regulation of MIER1 is ER stress related. Some experiments to address this could be performed or the text should be altered to reflect other possibilities.

Response: Thanks very much for the comment. We recognized and agree with the reviewer that the causal link between EIF2S1 and the translational regulation of MIER1 is weak in this study. To further support that ISR is at least partly involved in MIER1 regulation during regeneration, we further treated the animals with an ISR inhibitor, ISRIB, after liver hepatectomy. ISRIB blunts the ISR by allosterically antagonizing the inhibitory effect of p-eIF2 on eIF2B. Indeed, ISRIB treatment led to significantly inhibited MIER1 downregulation during normal liver regeneration (**Revised Figure S7B**), further suggesting a causative association between EIF2S1 phosphorylation and MIER1 downregulation.

Revised Figure S7

B

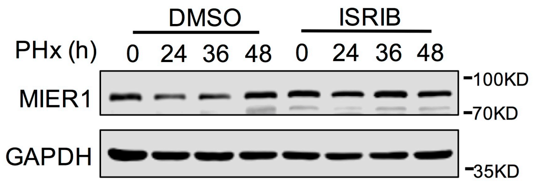

In the discussion part, we mentioned several previous studies, which also reported a rapid increase and activation of ER-stress related genes in the physiological responses during liver regeneration after hepatectomy. However, as advised by the reviewer, as we do not have evidences supporting that the MIER1 regulation is ER-stress related, we modified the text, and stressed that "*whether the acute ER stress is the direct inducer of ISR, or different mechanisms other than ISR, that regulates MIER1 synthesis during liver regeneration warrants further investigat*ion".

6. One of the most striking findings in this study is presented in Figure 5. This finding would be significantly strengthened if the authors were too tie this finding back to the epigenetic regulation of the cell cycle genes that they propose MIER1 to regulate (i.e. genes identified through ChIPseq).

Response: Thanks much for the suggestion. We further performed RNA-seq studies with these animals. We indeed observed a clear enhancement in expression of cell cycle-related genes upon MIER1 depletion in the *Lipe*-AKO animals (**revised Figure S9G**). The heatmap presented the top 100 differentially expressed cell cycle-relevant genes in liver tissues collected at 36 h post-surgery between wild-type (Control) and *Lipe*-AKO animals, in which we found that further liver MIER1 depletion in *Lipe*-AKO animals significantly recovered these gene expression.

Revised Figure S9

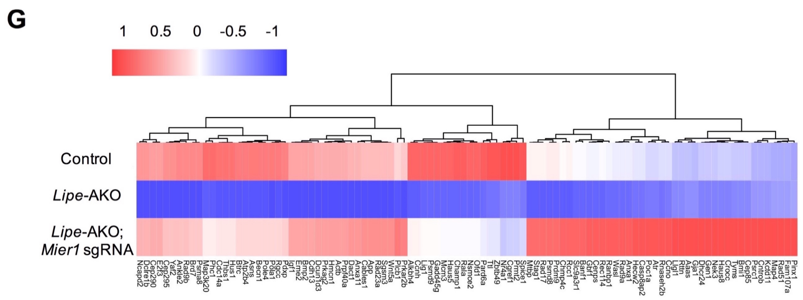

7. The aging study is very interesting. Is MIER1 occupancy different in aged animals? Does the cell cycle gene expression pattern restored in the aged animals where MIER1 is depleted? Similar questions for the mice on a chronic HFD.

Response: Thanks much for this great question. We apologize that we currently cannot directly assess the change of MIER1 occupancy in aged animals. Due to a lack of available MIER1 antibody for ChIP-seq analysis, we exogenously expressed MIER1-FLAG under a TBG promoter in liver tissues, and conducted the ChIP-seq using FLAG antibody. We thus currently are not able to assess the occupancy of MIER1 in aged animals, as it would take more than one year to prepare the animals. Instead, we performed RNA-seq analysis with liver tissues collected during liver hepatectomy in

aging animals and animals subjected to chronic HFD feeding. Similar to changes with liver MIER1 depletion in young animals with normal chow diet, we observed a significant increase in expression of cell cycle relevant genes in these animals (**Revised Figure S11D, S11J**). Based on the similar changes to cell cycle –relevant genes, we propose that MIER1 binds to similar cell cycle-relevant genes in aged and crHFD-fed animals, although direct analysis of MIER1-bound loci needs to be carried out before a conclusion can be finally made.

To be noted, genes listed in the heatmaps are the top 100 differentially expressed cell cycle-relevant genes in liver tissues collected at 48 h post-surgery between NCD-fed and crHFD-fed (**Revised Figure S11D**), or between young and aging animals (**Revised Figure S11J**). We found that liver MIER1 depletion in crHFD-fed and aging animals can significantly recover these cell cycle gene expression, although not to the level of the healthy or young control animals.

Revised Figure S11

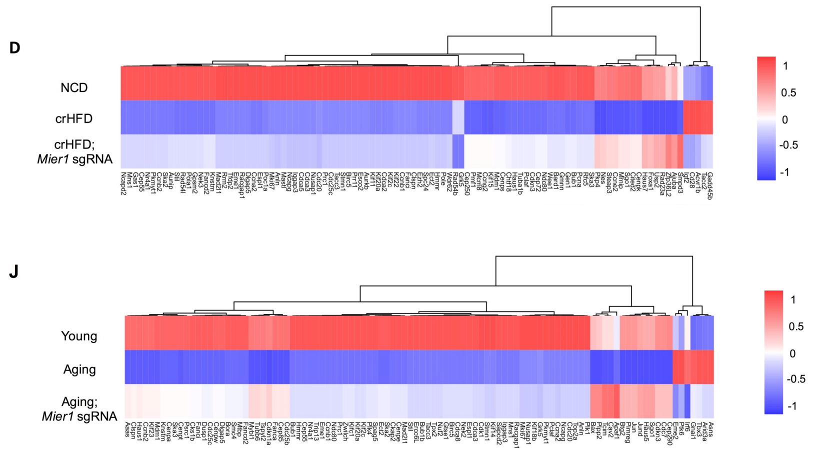

Minor

1. The screen is described as focused on epigenetic regulators, but many of the genes on the list that were screened do not serve this function directly – for instance, there are lots of signaling molecules, metabolic enzymes, toxicant response factors and others that are, at best, distantly and indirectly associated with chromatin. There are also important epigenetic regulators that are not on the list. More accurate descrition of the

pool of genes screened is needed.

Response: Thanks for noticing this. We modified the description of the library in revised manuscript, and to be more accurate, we also included the gene list in **Supplementary Data 1**.

2. H3k27ac is a mark of enhancers and it is not clear why is the H3k27ac enrichment at promotors is investigated (Figure 2F-G).

Response: Thanks for pointing this out. We initially are not sure about how MIER1 regulates gene expression. As previous reports suggested that MIER1 interacts with HDAC1 and HDAC2, and our data also showed that the majority of MIER1-bound peaks overlapped with H3K27ac signals, we thus wanted to look at H3K27ac. Nevertheless, in the revised manuscript, as we found that HDAC1 and 2 may not involve in MIER1-mediated chromatin remodeling, we moved H3K27ac-related data to **Revised Supplemental Figure S4C, S4D, and S4E**, in the hope that these data may still be helpful to the future investigation of MIER1 function.

3. Many of the graphs lack information under each bar or legends are attached to a different panel (example Figue 1E, 7A, 7D, 7I). Labeling on all figures should be checked and corrected.

Response: Many thanks. We have all figures carefully checked and revised if needed.

4. There are a number of grammatical or usage issues that need to be cleaned up (i.e. “the steatosis” is used throughout instead of “steatosis”)

Response: Many thanks. We have these grammatical or usage issues in manuscript carefully revised.

5. Changing the use of words like “role for” or regulates without implying what the specific role or regulation would make the paper easier to read.

Response: Many thanks for the careful reading. We have the manuscript thoroughly

checked and revised.

6. Figure 4A is very hard to interpret. It would be better to change the way this data is presented.

Response: Many thanks. We tried a couple of different graphs, and as the portion of many lipid species are very small, it is still hard to make it clear to see changes of all species within one graph. We thus included the changes of the concentrations of each lipid species in supplemental figures (**Revised Figure S6C, and S6D**).

Revised Figure S6

C

Conc. (mg/g)	TAG	SM	PE	PC	LPE	LPC	HexCer	Hex2Cer	FFA	DAG	Cer	CE
0h	10.00832	1.137107	2.752532	17.63879	0.137463	0.159877	0.035041	0.003128	3.649549	2.016099	0.356144	1.689411
24h	79.47927	1.297691	3.706209	19.07111	0.152102	0.172028	0.068925	0.006992	4.205505	3.72701	0.45412	4.901113

D

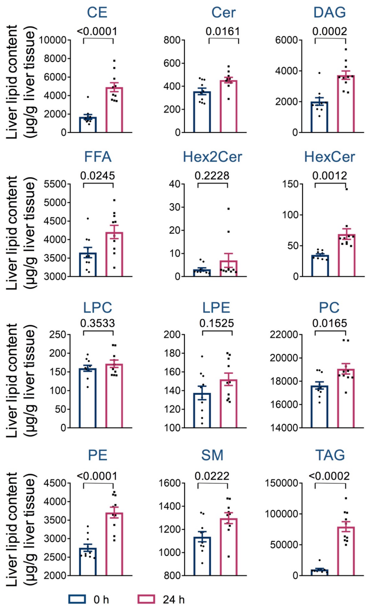

Reviewer #3 (Remarks to the Author):

The manuscript by Chen and Chen et al identified a new role for protein MIER1 in hepatic regeneration. The manuscript uses a nice in vivo CRISPR screening system and a Fah-/- mice to identify new regulators in liver regeneration. Depletion of Mier1 was discovered to improve liver regeneration. Using genetic, molecular and biochemical tools, the study suggests that acute steatosis impairs MIER1 translation, which in turn changes chromatin accessibility to promote transcription of cell cycle genes and liver

regeneration. Furthermore, this regulatory process was impaired in murine models of aging and chronic steatosis. This is an interesting study that has novelty. However, there are concerns involving experimental rigor and interpretation. The rigor of the MIER1 translation conclusion is not convincing and there are figures of insufficient quality. Another concern is that the study does not address these results to the human liver. No primary or immortalized human hepatocytes were studied in the context of the MIER1 mechanism. Addressing these concerns is important to support the stated conclusions.

Response: We appreciate the comments from this reviewer. To address these issues, we performed additional experiments, e.g. additional characterization of MIER1 in human liver organoids derived from human pluripotent stem cells. We have also improved quality of some figures. Please find below the specific responses to each comment.

Major concerns:

1. The key findings of this manuscript need to be assessed in human hepatocytes/tissues to address the potential translation of this work to human liver transplant/regeneration.

Response: Thanks for this very nice suggestion. We are unfortunately not able to obtain clinical human liver tissues relevant to the regeneration issue. And we do not think tumor cell lines are suitable here for studying the physiological function of MIER1 during liver regeneration. In order to further test the function of MIER1 in a human system, we generated human liver organoids derived from human pluripotent stem cells (hPSCs), following a previously established protocol, which can partly recapitulate the organogenesis events during liver regeneration. Interestingly, when *MIER1* was knocked down using the lenti-Cas9/CRISPR system in hPSCs, the size of liver organoids is clearly larger than wild-type liver organoids (**revised Figure S3A**). In addition, expression levels of several cell cycle markers, including Cyclin A2, Cyclin D1, PCNA, are significantly enhanced in liver organoids after MIER1 depletion (**revised Figure S3B**), suggesting a potential similar function of MIER1 in promoting human liver cell proliferation. No obvious difference was observed in hepatocyte

markers, indicating that MIER1 depletion does not affect the liver characteristics (**revised Figure S3C**). Altogether, these data suggested a potential similar function of MIER1 in promoting human liver cell proliferation during regeneration.

Revised Figure S3

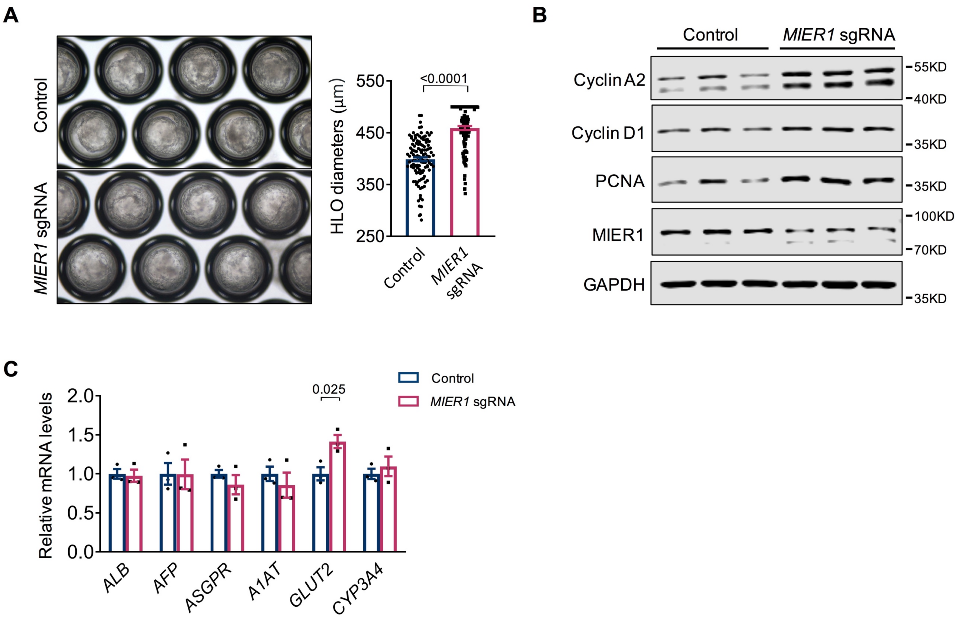

2. The manuscript should measure/show steatosis in the tissues to confirm their model. Furthermore, the manuscript indicates that mice received HFD for 8-9 weeks in the text of the results, but the rest of the manuscript indicates 8 weeks. Please clarify.

Response: Thanks for pointing this out. We included the analysis of liver triglyceride content in animals subjected to long-time HFD feeding (crHFD) and in aging animals (**Revised Figure 7A and 7F**), both of which showed significant increase in liver lipid level. We also revised the text and made it consistent in the description that the animals received HFD for 8 weeks before liver hepatectomy. Many thanks for noticing this.

Revised Figure 7

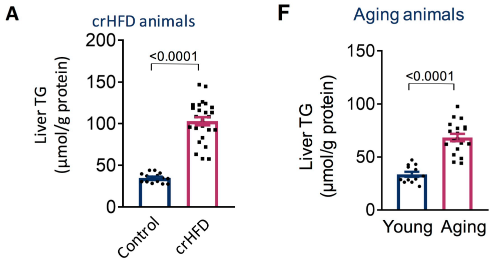

3. The levels of cyclin D1 increases with MIER1 loss in figure 1, but also increased with MIER1 overexpression, and shows no change with MIER1 KO in the HFD model. How to justify the inconsistent changes in cyclin D1 in the manuscript?

Response: Thanks for picking this out. And apologize that after carefully looking into the data, we noticed that in the previous data presentation, we have made a mistake that we mixed up the control and MIER1 overexpression group when calculating the change of *Ccnd1*. We confirmed that the levels of *Ccnd1* increased with MIER1 loss, and decreased with MIER1 overexpression. We have the corrected figure presented in the revised manuscript (**Revised Figure 3F**).

Revised Figure 3

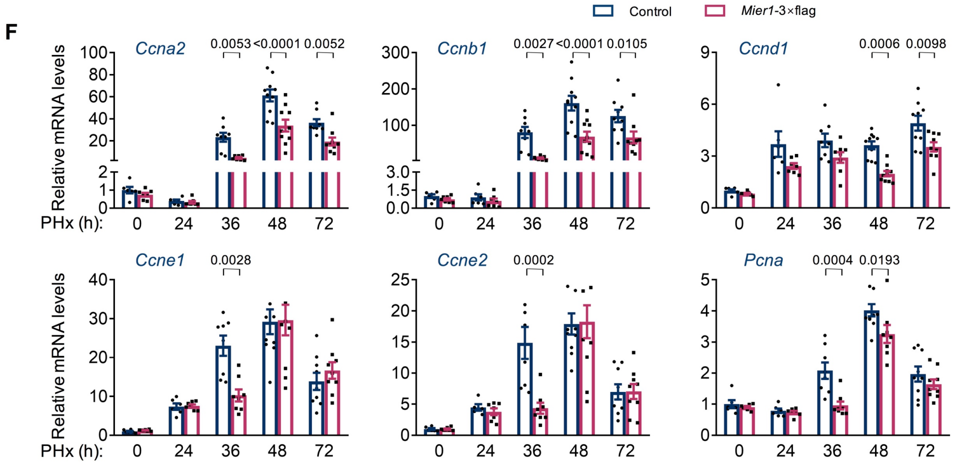

Regarding the change of cyclin D1 in crHFD animals, we indeed noticed an increase of cyclin D1 both in protein and in mRNA levels in crHFD animals, as compared to animals subjected to NCD feeding, suggesting that high-fat diet feeding may increase

liver cyclin D1 expression. Liver depletion of MIER1 in crHFD animals showed no further increase in cyclin D1 level in crHFD animals. We currently do not know the exact mechanism under this phenomenon. However, regarding the effect of MIER1 to liver regeneration capacity in crHFD animals, we did observe increased liver/body weight, Ki67-positive cells, and PCNA expression after liver MIER1 depletion (**Revised Figure 7D,7E, S11C**).

In addition, to get a thorough understanding of liver MIER1 depletion to cell cycle gene transcription in crHFD animals, we performed RNA-seq analysis in liver tissues collected from 48h after liver hepatectomy in crHFD animals with or without liver MIER1 depletion (**Revised Figure S11D**). We found that liver MIER1 depletion in crHFD-fed animals can significantly increase the cell cycle gene expression, although not to the level of the healthy control animals. To be noted, genes listed in the heatmap are the top 100 differentially expressed cell cycle-relevant genes in liver tissues between NCD-fed and crHFD-fed animals. These data altogether have supported our main point that MIER1 depletion increased cell cycle gene expression and liver regeneration in crHFD-fed animals.

Revised Figure S11

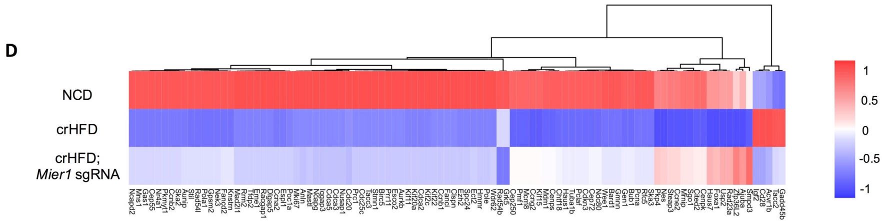

4. mRNA is translated, protein is synthesized. The explanation for looking at eIF2S1 (eIF2alpha) phosphorylation should be clearer. Saturated free fatty acids have been reported to induce ER stress, trigger eIF2 phosphorylation (PERK eIF2) kinase which broadly lowers translation initiation. The experiments in Fig. 4H, especially the Mier1 mRNA placement in in sub (difficult to see monosomes or free ribomses vs polysomes were not convincing and lacked controls (other mRNAs known to be differentially

regulated by the UPR/ISR, such as does ATF4 shift towards suggested polysomes). Overall, the conclusion the conclusion that there is dynamic change in translational, contributing to MIER1 reduction, was not sufficiently supported by the presented results.

Response: We appreciate the critical comments raised by this reviewer. To address the issues, we have the following explanations, also with additional characterizations.

1) We initially observed a transient decrease in MIER1 protein during early liver regeneration, and further studies suggested that *Mier1* is translationally regulated during liver regeneration. Induction of EIF2S1 phosphorylation, which limits ternary complex recycling, and inhibition of mTORC1 are two major mechanisms of translational suppression. As we observed a dynamic change of liver p-EIF2S1-S51 *in vivo* during liver regeneration that was highly associated with the change in MIER1 protein level, we decided to focus on the EIF2S1 signaling pathway.

We did recognize that the causal link between EIF2S1 and the translational regulation of *Mier1* is weak in this study. To further support that ISR is involved in MIER1 regulation during regeneration, we treated the animals with an ISR inhibitor, ISRIB, after liver hepatectomy. ISRIB blunts the ISR by allosterically antagonizing the inhibitory effect of p-eIF2 on eIF2B. Indeed, ISRIB treatment led to significantly inhibited MIER1 downregulation during normal liver regeneration (**Revised Figure S7B**), further suggesting a causative association between EIF2S1 phosphorylation and MIER1 downregulation.

Revised Figure S7

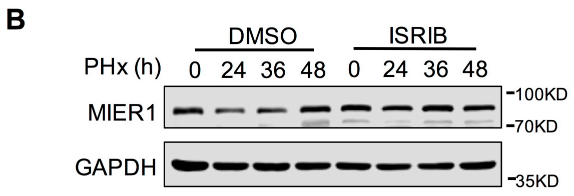

2) Please be noted that the polysome profiles presented in Figure 4H is in values of OD260 nm/OD280 nm after RNA extraction, which is different from the continuous recording of absorbance at 260 nm using the automatic density gradient separation system. In addition, to assess the profile of specific transcripts, the fractionations at different gradients after ultracentrifugation were separated into 25 fractions with equal volume, so the profiles presented in Figure 4H were in less resolution than the continuous recording of 260 nm absorbance.

In the revised manuscript, we have a representative figure with continuous OD 260 nm recording using the automatic density gradient separation system also presented, in which clear 40S, 60S, 80S and polysomes can be observed (**Revised Figure S8A**). Please be noted that above is the OD260 nm absorbance recording, below is the gel picture of extracted RNAs in each fraction of the collected 25 fractions. We can see from this result a clear shift of transcripts to sub-polysomes in liver tissues at 24 h after hepatectomy, as compared to ones before surgery.

Revised Figure S8

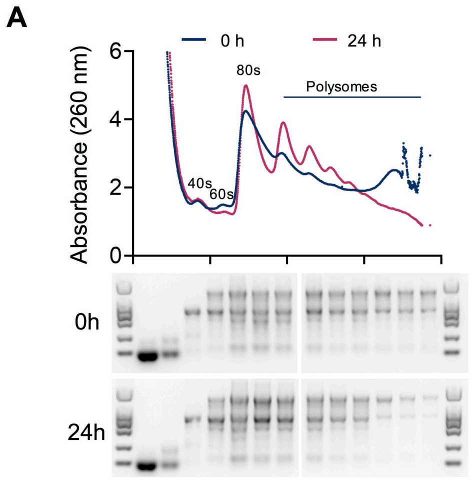

3) As advised by the reviewer, we also looked at the distribution of *Atf4* transcript. Although the result showed that most *Atf4* transcripts were associated with sub-polysomes, a significant increase in polysome-associated *Atf4* transcripts was observed in liver tissues from 24 h post-surgery, suggesting the translational induction of ATF4

(**Revised Figure S8B**).

Revised Figure S8

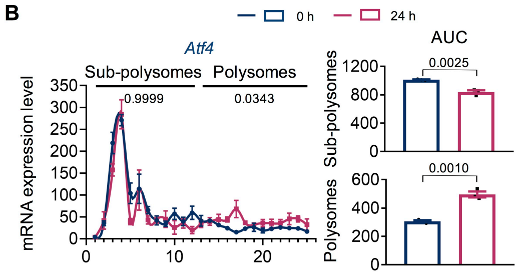

In addition, based on our observations, although more direct evidences are surely needed, we suspect that the physiological and transient ISR induced during liver regeneration is different from the long-time, chronic ISR, for example, in some diseased conditions, in which the shift of the *Atf4* transcripts to polysomes is dramatic. The ribo-seq results in our study also showed specific patterns of transcripts with changes of translational activity in the physiological and transient ISR during liver regeneration (**Revised Figure 4I, 4J, and 4K**). Further functional and mechanistic investigations are warranted to tell the differences between the transient ISR and the chronic ISR.

5. There are also some concerns about the rigor of polysomes and measured mRNAs in fractions for assessing translation repression of MIER1 in Figure 5.

Response: Many thanks. In Figure 5, we analyzed the change of MIER1 protein during early liver regeneration after reducing the liver lipid accumulation by adipose specific knocking out of HSL (*Lipe* AKO animals), a key enzyme in lipolysis. Our results showed compromised MIER1 regulation and blunted changes in p-EIF2S1 when lipid accumulation was affected in *Lipe* AKO animals. The polysome profile and mRNA measurement further supported the compromised regulation of MIER1 in *Lipe* AKO animals, and the approach we used to perform the analysis was the same as one adopted previously. These results altogether supported that the translational regulation of MIER1 is affected due to reduced liver lipid accumulation during regeneration in *Lipe* AKO animals.

6. Quantification of western blots is needed in several figure panels (Figure 1F, Figure 3C, Figure 4C, Figure 5A, D, I, Figure 6F, Figure 7B, C, G and H, Figure S2I, Figure S7, Figure S8B, C, E, F, I), The quantitation is needed to support key conclusions and confirm consistency of biological replicates for experiments where only 2 replicates are shown in the WB. Some blots appear overexposed and/or of poor quality (Figure 4F, Figure 5A, Figure 6F PCNA, Figure 8H, EIF2S1; Figure S8E)

Response: Thanks very much for the suggestion. We included the quantification of western blots above each image in the revised figures, and specified the number of times the WB experiments have been independently repeated for in the figure legends (**Revised Figure 1F, Figure 3E, Figure 4C, Figure 5D, 5I, Figure 6F, Figure 7B, C, G and H, Figure S5A, Figure S10A, Figure S11B, C, E, F, H, I**). We also re-did the blots with low quality, and have new better images replaced in the revised manuscript (**Revised Figure 4F, Figure 5A, Figure 6F, Figure S11E**).

Minor concerns:

1. Portions of the abstract are not clear. Factors and pathways, EIF2S and Lipe knockdown are referred to without sufficient introduction. Furthermore, the subjects and meaning of some sentences are not fully clear. It would be helpful to simplify some sentences.

Response: Many thanks for the suggestion. We rewrote the abstract in the revised manuscript.

2. The title for Supplementary Figure 9 indicates a comparison between chronic and acute steatosis, but the names of the groups in comparison to the title is confusing. Provide better descriptions to improve clarity.

Response: Many thanks. We have revised the title and the label in the figure **(now revised Figure S12)**.

3. Several graphs appear to be missing labels, or the legends are in awkward locations

and not near the graphs themselves. This hampers the clarity of the manuscript.

Figure 1E

Figure 3D and F

Figure 5D, F, G, I

Figure 6D and E

Figure 7A, D, I

Response: Many thanks for picking these up. We have carefully checked all figures and revised labels in these figures.

Reviewer #4 (Remarks to the Author):

Chen and colleagues have conducted a series of well thought out and logical studies that have defined a novel role for Mier1 in regulating liver regeneration. There are a number of strengths to this paper including the use of an in vivo screen for factors that regulate liver regeneration, a mix of in vivo and in vitro studies with sophisticated molecular approaches, and the overall conceptual novelty of the work. However, there are some things to be improved.

The studies focus a great deal on the transient steatosis that occurs with liver resection in mice. However, it is less clear that this is relevant in humans for a variety of reasons. The severity of the partial hepatectomy likely causes anorexia in the mice and even acute fasting in mice can drive steatosis. In humans, caloric restriction for the same duration would lead to reduced liver fat and only after several days would liver fat accumulation manifest.

Response: Thanks for the comments. This study mostly relies on the animal models, and we appreciate the reviewer for bringing out the differences between human and mouse.

As advised by the reviewer, we did the sham surgery in the revised manuscript. We observed similar decrease in food intake at day 1 in animals with sham surgery as compared to animals under liver hepatectomy, but no significant liver lipid

accumulation in animals with sham surgery. We therefore do not think the decreased food intake in animals drive the liver steatosis during early regeneration.

Revised Figure S5

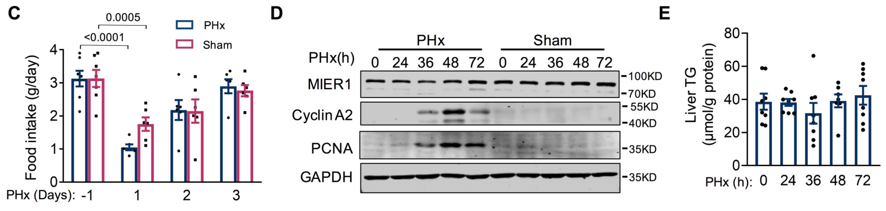

Given the specific regeneration topic this study has been focusing, we are unfortunately not able to obtain clinical human liver tissues relevant to the regeneration issue. We have also searched literatures, and have not seen any report with specific characterization with human liver tissues shortly after liver hepatectomy. Whether this acute liver lipid accumulation also happens during human liver regeneration remains to be discovered. Nevertheless, the acute lipid accumulation during liver regeneration has been well established in animal models, including rat and mouse. And it is still unclear how this acute lipid accumulation regulates liver regeneration in these animal models. The discovery that the epigenetic regulator MIER1 bridges the acute liver steatosis and chromatin remodeling and cell proliferation during liver regeneration is our main finding here.

In order to further test the potential function of MIER1 in a human system, we generated human liver organoids derived from human pluripotent stem cells (hPSCs), following a previously established protocol, which can partly recapitulate the organogenesis events during liver regeneration. Interestingly, when *MIER1* was knocked down using the lenti-Cas9/CRISPR system in hPSCs, the size of liver organoids is clearly larger than wild-type liver organoids (**revised Figure S3A**). In addition, expression levels of several cell cycle markers, including Cyclin A2, Cyclin D1, PCNA, are significantly enhanced in liver organoids after MIER1 depletion (**revised Figure S3B**), suggesting a potential similar function of MIER1 in promoting human liver cell proliferation. No

obvious difference was observed in hepatocyte markers, indicating that MIER1 depletion does not affect liver characteristics (**revised Figure S3C**). These data altogether suggested a potential similar function of MIER1 in promoting human liver cell proliferation during regeneration.

Revised Figure S3

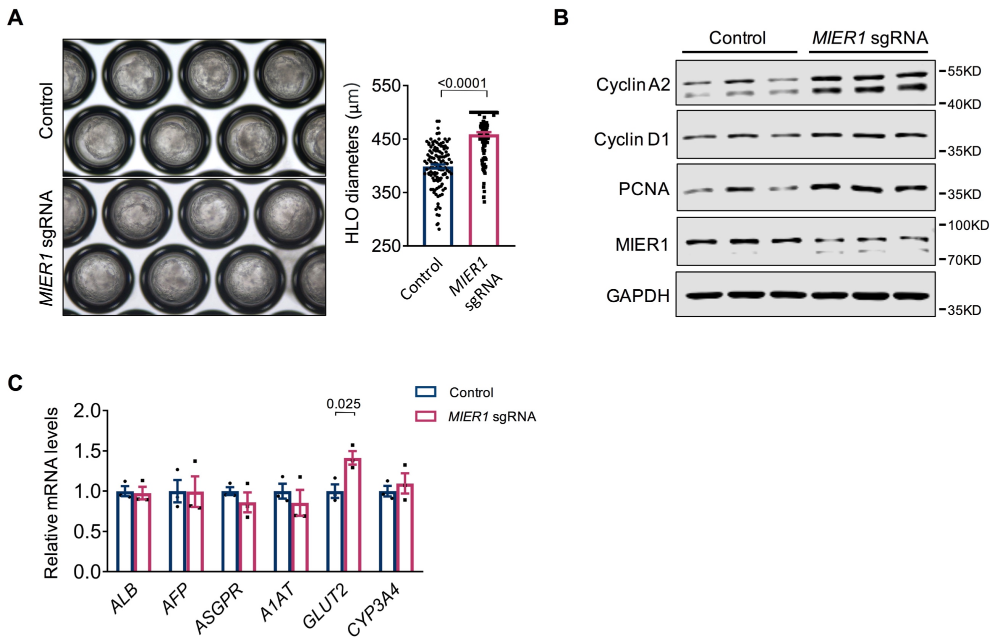

There are also many questions about the lipid accumulation in these mice.

1. Can the authors determine why adipose tissue lipolysis is increased (humoral, neuroendocrine, food intake)?

Response: Thanks for this great question. We observed clear increase of HSL phosphorylation in adipose tissues shortly after liver hepatectomy (**Revised Figure S9C, S9D**, details please see responses below). In addition, the liver lipid accumulation was significantly reduced in adipose specific HSL knockout animals (**Revised Figure 5A, 5B**). We therefore suspect that liver hepatectomy activates lipases in adipose tissues, which subsequently lead to increased lipolysis in adipose tissues. Unfortunately, although efforts being made, we currently do not know the specific mechanisms underlying the activated adipose lipase during liver regeneration, which is certainly an interesting direction worthy deeper investigating in the future.

1) Previous reports suggested that liver can regulate systemic lipid catabolism via a neuronal pathway, in that signals of the afferent vagus from the liver are relayed to the sympathetic nerves to white adipose tissues, which stimulate lipolysis. To test whether this neuronal pathway is involved in activated adipose lipolysis in early liver regeneration, we performed selective hepatic vagotomy (HVX) surgery before animals were subjected to liver hepatectomy. In the HVX surgery, the hepatic branch of the vagal trunk was transected. The results showed that HVX caused no significant effect to HSL phosphorylation in adipose tissues at 8 h after liver hepatectomy, nor liver lipid accumulation or liver regeneration as analyzed at 24 h after liver hepatectomy.

Analysis at 8 hours after liver hepatectomy

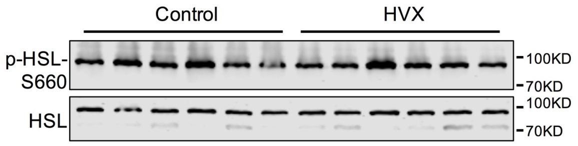

Analysis at 24 hours after liver hepatectomy

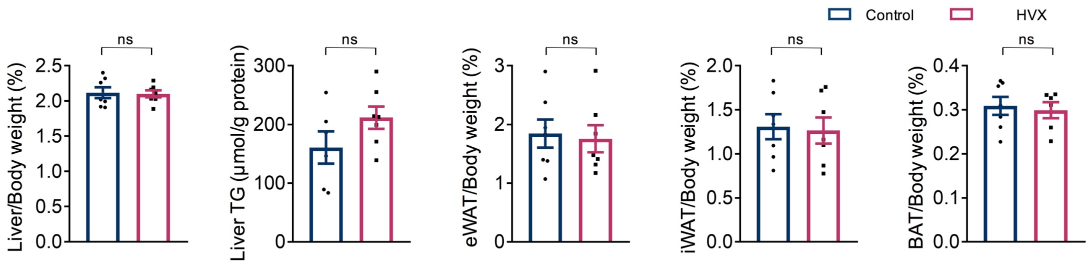

2) As advised by the reviewer, we monitored the food intake of animals after liver hepatectomy. Results showed a significant decrease in food intake at day 1 after surgery (**Revised Figure S5C**). Sham surgery with no hepatectomy was also performed (thanks to the reviewer's reminding), in which reduced food intake was also noticed. However, no significant liver steatosis was observed in animals with sham surgery, suggesting that reduced food intake was not the primary reason for increased liver lipid accumulation during regeneration.

Revised Figure S5

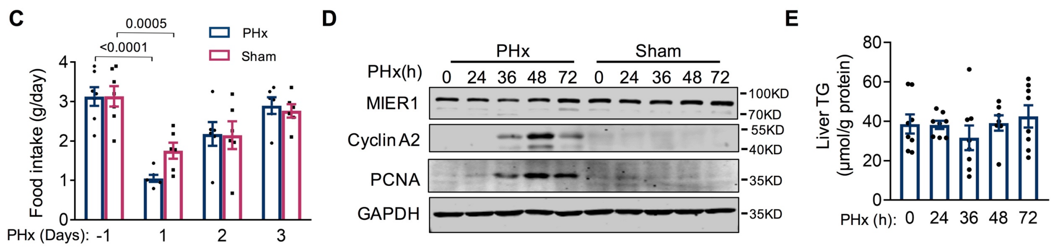

Besides the neuronal pathway and effects of reduced food intake, many other factors, e.g growth factors, immune factors, blood glucose, all display a dynamic change at the early onset of liver regeneration, which may involve in the activation of adipose lipolysis. It seems that deeper investigations with heavy input would be required to answer this question, which is also an independent direction from the main focus in this study. We thus decide to leave this question for future study.

2. Is the phosphorylation of HSL and PLIN1 increased after PH?

Response: Thanks for the suggestion. We checked the phosphorylation of HSL after PHx. We did observe a significant increase of HSL phosphorylation at as early as 8h after PHx in the early onset of liver regeneration in adipose tissues—epididymal white adipose tissues (**Revised Figure S9C**) and inguinal white adipose tissues (**Revised Figure S9D**), suggesting the involvement of HSL in lipid redistribution induced by liver regeneration. We unfortunately could not do analysis on the phosphorylation of PLIN1 due to the lack of available antibody, especially with the interference of the Shanghai Covid-19 lockdown, which have made the import of biological materials from overseas very hard.

Revised Figure S9

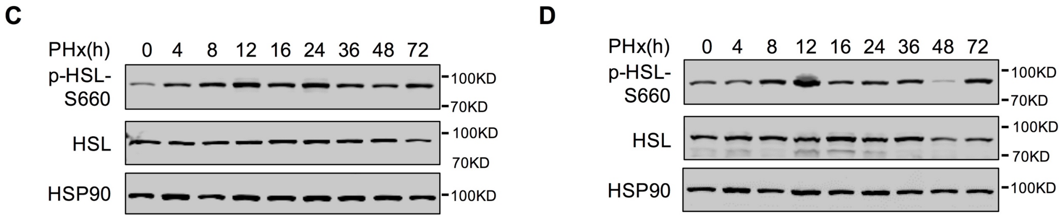

3. Plasma free fatty acids and glycerol should be measured after PH and in the HSL KO mice.

Response: Thanks for the suggestion. We measured plasma free fatty acids after PH in control and adipose specific HSL knockout animals. Consistent with compromised adipose lipolysis in the HSL KO animals, we observed reduced plasma serum free fatty acids during liver regeneration in HSL KO mice (**Revised Figure S9F**).

Revised Figure S9

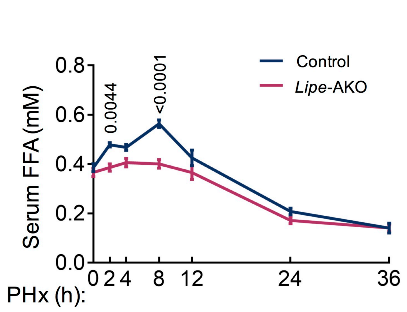

Recent work has defined the zonal subpopulations of hepatocytes that form the major regenerative pools. Is Mier1 expressed uniformly across all zones of the liver? Although liver fat is measured by biochemical assays, there is no data on the spatial distribution of the fat. Is this zonal as well? Is there a negative correlation between Mier1 expressing cells and lipid accumulation?

Response: Thanks for this great question. We so far did not observe clear zonal difference in MIER1 localization in liver via IHC analysis. Regarding the spatial distribution of liver fat, we did notice more lipid accumulation at the portal vein as

compared to lipid accumulation near the central vein at 36 h after liver hepatectomy from liver sections (**Revised Figure S2F**). Regarding the MIER1 level, we found weaker expression of MIER1 in some hepatocytes with more lipid accumulation, especially near the portal vein (**Revised Figure S2F**). However, we think it is probably immature to make any conclusion based simply with these observations. Detailed analysis to the zonal issue with specific genetic tools are warranted in the future.

Revised Figure S2

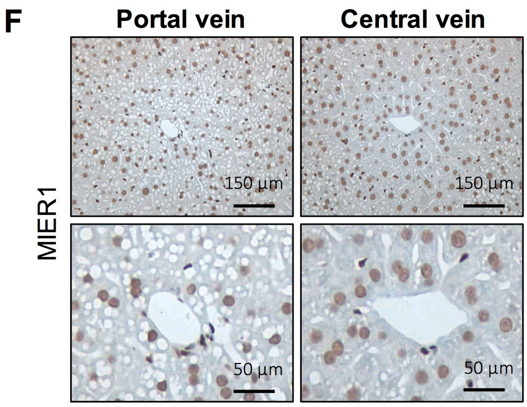

There are some methodologic issues that need to be addressed.

1. A normal chow is not the appropriate control for the 60% fat diet. The HFD is made up of defined components and also has added sucrose. Normal chow is not made of refined components and has no added sugar. A matched low fat control chow needs to be used and the effect of added sugar also needs to be addressed. Without this important control, the conclusions from the HFD studies are not supported. These studies need to be re-done with this important control.

Response: Many thanks for the comment. We unfortunately cannot purchase the matched low-fat control chow from Research Diets in time, as the Covid19 lockdown have made the import of biological materials from overseas very hard and rather slow. Instead, to assess whether added sugar in HFD may also interfere with liver regeneration, we fed the animals with extra glucose and fructose before being subjected

to liver hepatectomy. The results showed that extra sugar did not change the MIER1 regulation, cell proliferation, nor liver lipid accumulation during regeneration (**Revised Figure S10B, S10C**).

Revised Figure S10

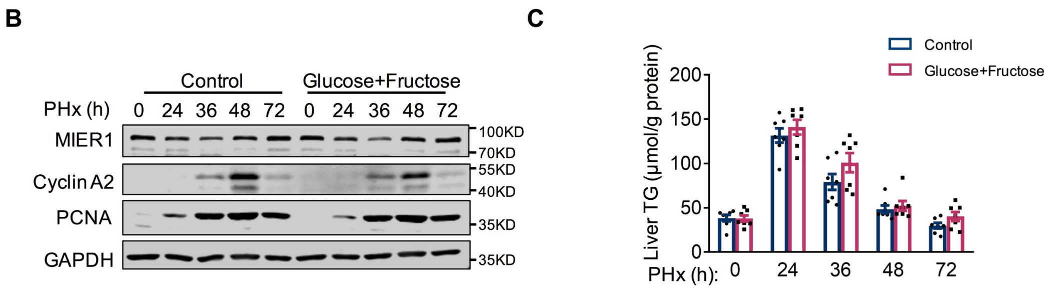

2. For the in vitro studies with fatty acids, I could not find specified what the concentration of fatty acids was in these studies. Please specify. Also, the saturated fats were found to have effects on Mier1 and translational control. Care must be taken in interpreting these results as high levels of sat fat are toxic. It's also not clear that high levels of saturated fatty acids are physiologic, since in vivo the liver is exposed to both saturated and unsaturated fatty acids.

Response: Many thanks for the suggestion. We included the concentration of fatty acids in the corresponding figure legends (**Revised Figure S6F**, **and Figure 4C**).

To assess the potential effect of liver lipids to MIER1 regulation, we first performed lipidomic analysis in liver tissues collected before and 24h after surgery, when the liver lipid content reaches its peak (**Revised Figure 4A, S6C, S6D, S6E**). Further calculation of the major fatty acids that comprise increased liver lipids in liver at 24h after surgery showed that the palmitic acid (PA, 16:0), stearic acid (SA, 18:0), oleic acid (OA, 18:1), and linoleic acid (LA, 18:2) are the most abundant fatty acids in the lipid composition (**Revised Figure 4B, Supplementary Data 4**). We thus first evaluated the effect of different FFAs to MIER1 regulation using mouse primary hepatocytes. Cells were treated with 1mM of PA, SA, OA and LA for 10 hours and analyzed for MIER1 protein level, respectively (**Revised Figure S6F**). We observed clear decrease of MIER1

expression when cells were exposed to FFAs (**Revised Figure S6F**). We next treated primary hepatocytes with PA (0.5mM) for 5 h, 10 h and 20 h, or treated primary hepatocytes with different concentrations of PA (0.1 mM, 0.5 mM and 1mM) for 10 h, and we observed a time- and dose-dependent decrease in MIER1 protein (**Revised Figure 4C**). And in comparison, treatment of PA (0.5mM) for 10 h to primary hepatocytes isolated from crHFD-fed or aging animals showed no effect on MIER1 regulation (**Revised Figure 7C and 7H**).

Revised Figure S6

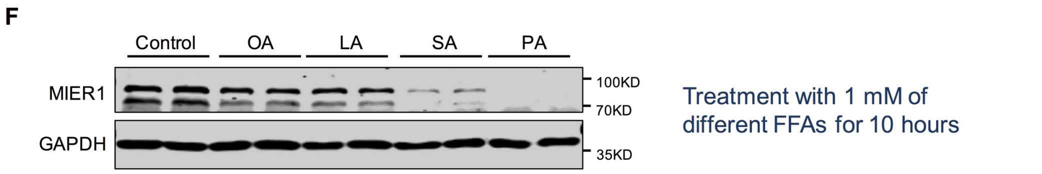

Revised Figure 4

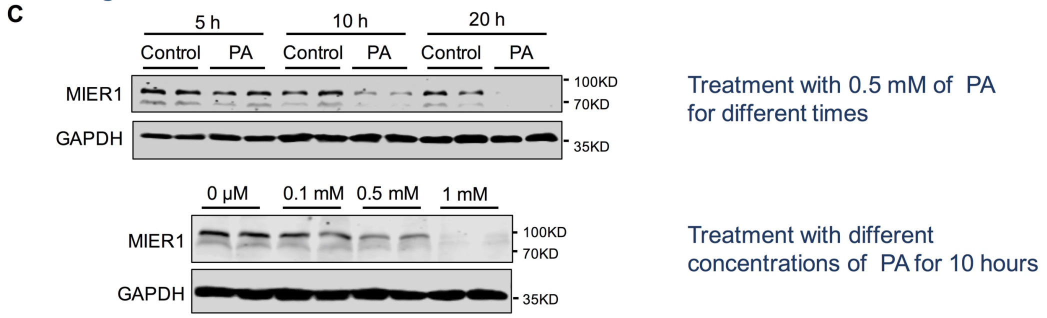

Revised Figure 7

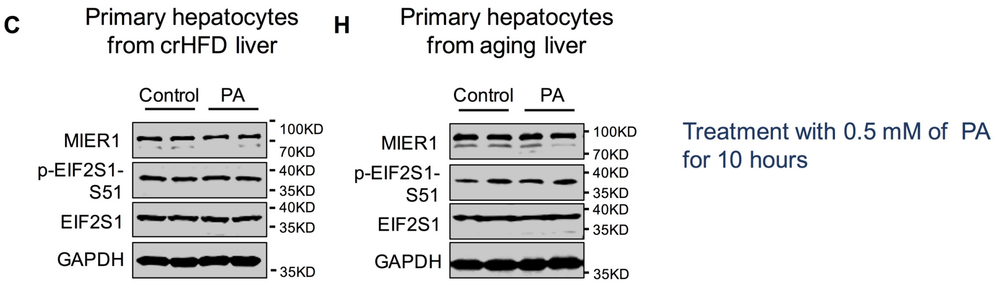

Based on these results, we can tell that both saturated (PA, SA) and unsaturated (OA, LA) can affect MIER1 expression. In addition, different effects with PA treatment to primary hepatocytes from healthy, young animals and ones from aging and crHFD-fed animals were observed, suggesting different regulatory mechanisms in these cells. Also considering the results from the *in vivo* analyses with animals from different

groups and with different genotypes, we think the conclusion that liver lipid accumulation during early regeneration regulates *Mier1* translation is well supported.

3. There were no sham surgery animals included in these studies. Does hepatic steatosis occur with laparotomy but no PH? This important control should also be included.

Response: We appreciate the suggestion. We included sham surgery in the revised manuscript (**Revised Figure S5C, S5D, S5E**). We observed a similar decrease in food intake at day 1 after sham surgery (**Revised Figure S5C**), however, no significant liver steatosis was observed (**Revised Figure S5E**).

Revised Figure S5

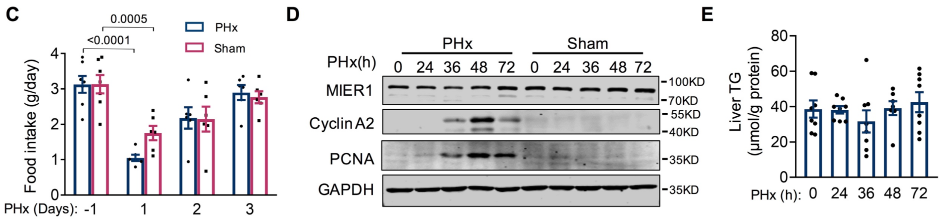

The effect of the acute HFD feeding on liver TG is very modest. Moreover, the HFD did preload the liver with lipid (as was the stated intention) since there was no increase in TG at time 0. It is not clear exactly what is happening in this experiment since the HFD also has added sucrose compared to the control chow. Food intake after PH should also be assessed.

Response: We appreciate the comment. In order to assess whether an earlier boost in liver lipid accumulation can cause earlier MIER1 reduction and accelerated liver regeneration, we did the acute HFD feeding experiment. We pre-fed the animals with HFD 2 hours before surgery and continued HFD feeding after surgery until sacrificing for analysis. We observed no significant increase in liver lipids at time 0 (when we did the surgery), possibly due to the relatively short time in HFD feeding. We did observe an increase in liver lipid accumulation at as early as 12h after surgery (**Revised Figure 6B**), and accordingly, a significant enhancement of the p-EIF2S1 signal and reduction

in MIER1 level after surgery (**Revised Figure 6C, S10A**), and increased regenerative capacity (**Revised Figure 6D-F**).

As also mentioned above, to control the possible effect of added sugar in HFD diet, we pre-fed the animals with extra glucose and fructose before being subjected to liver hepatectomy. And the result showed that extra sugar did not change the MIER1 regulation, cell proliferation, nor liver lipid accumulation during regeneration (**Revised Figure S10B, S10C**). Food intake was also monitored (**Revised Figure S5C, S5D, S5E**), which displayed a reduction at day 1 after surgery. However, the reduced food intake does not seem to cause increased liver lipid accumulation, as similar reduction in food intake was observed in sham surgery, which did not lead to change in liver lipid accumulation (**Revised Figure S5C, S5D, S5E**).

Revised Figure S10

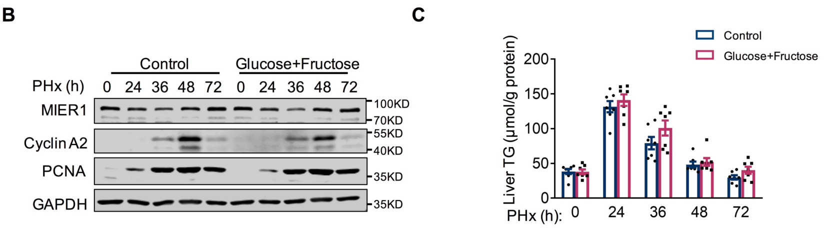

Revised Figure S5

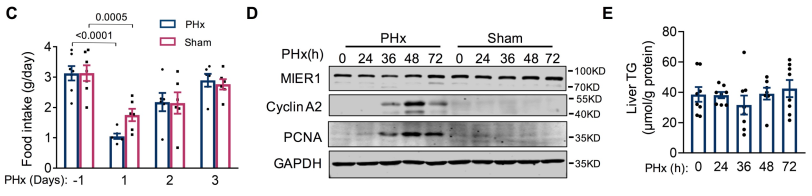

Minor:

Overall the manuscript is very well written. However, the sentence beginning on line 72 and running through line 77 is very long and doesn't completely make sense.

Response: Many thanks for picking this out. We rewrote the sentence in the revised manuscript.

Line 71. Technically, "energy" is not produced. The amount of energy in the universe

remains constant.

Response: Thanks. We modified the text in the revised manuscript.

REVIEWER COMMENTS

Reviewer #1 (Remarks to the Author):

The revised manuscript has addressed all my concerns.

Reviewer #2 (Remarks to the Author):

In this revised version of the manuscript, the authors went above and beyond in their response to the comments and addressed all concerns. The current version of the manuscript is thorough and describes a highly novel aspect of liver biology, shedding new insight into liver regeneration. It should be of great interest to the field. My only suggestion is to edit carefully for grammatical issues (i.e. instead of “the steatosis” in the title, just “steatosis”).

Reviewer #3 (Remarks to the Author):

The revised manuscript by Chen and Chen et al identified a new role for protein MIER1 in hepatic regeneration. Depletion of Mier1 was discovered to improve liver regeneration and the study suggests that acute steatosis impairs MIER1 translation, which in turn changes chromatin accessibility to promote transcription of cell cycle genes and liver regeneration. Furthermore, this regulatory process was impaired in murine models of aging and chronic steatosis. This is an interesting study that has novelty. Prior reviewer concerns were sufficiently addressed.

Reviewer #4 (Remarks to the Author):

My prior concerns were addressed.

Reviewer #5 (Remarks to the Author):

In the revised Manuscript "The transient liver steatosis translationally controls the epigenetic regulator MIER1 to promote liver regeneration" Chen et al. significantly enhance their findings by providing a considerable effort to address most of the reviewers concerns. They optimized their experimental design, improved their figures, and designed interesting new experiments. They modified their manuscript accordingly. In general it is a very interesting work, with novel findings that might have the potential to further our knowledge on human liver transplant and regeneration. This potential translation to the field of human liver transplant/regeneration is interestingly addressed using organoid models but unfortunately this part of the article is weak with several overstatements at that point. First bright-field images of the organoid structures do not match the images of the previously described

model and the total absence of characterization (staining for classical markers, hepatocyte functional assays...) makes it difficult to assess the quality of the organoid generation process. Moreover, since authors did not used a shMIER1 inducible system and provided very few images, it is very difficult to understand if the observed difference in organoid size is due to a difference in hepatocyte proliferation or rather to immature cells in the first steps of the protocol. A proper characterization of the day 22 hepatocyte organoid and a Ki67 staining/quantification might help enforce the data. Finally, the mRNA expression of a few markers of hepatocyte differentiation is too preliminary to state that MIER1 depletion does not affect human liver characteristics. Again, while I acknowledge that this is not the main part of the article but rather a way to open the study for future applications in human care, a minimal characterization of the organoid model and its evolution upon MIR1 depletion at protein, histological and or functional levels is mandatory to suggest that it might be involved in regeneration processes in human and might not affect the overall liver characteristics.

Reviewer #1 (Remarks to the Author):

The revised manuscript has addressed all my concerns.

Reviewer #2 (Remarks to the Author):

In this revised version of the manuscript, the authors went above and beyond in their response to the comments and addressed all concerns. The current version of the manuscript is thorough and describes a highly novel aspect of liver biology, shedding new insight into liver regeneration. It should be of great interest to the field. My only suggestion is to edit carefully for grammatical issues (i.e. instead of "the steatosis" in the title, just "steatosis").

Reviewer #3 (Remarks to the Author):

The revised manuscript by Chen and Chen et al identified a new role for protein MIER1 in hepatic regeneration. Depletion of Mier1 was discovered to improve liver regeneration and the study suggests that acute steatosis impairs MIER1 translation, which in turn changes chromatin accessibility to promote transcription of cell cycle genes and liver regeneration. Furthermore, this regulatory process was impaired in murine models of aging and chronic steatosis. This is an interesting study that has novelty. Prior reviewer concerns were sufficiently addressed.

Reviewer #4 (Remarks to the Author):

My prior concerns were addressed.

Response: We are truly grateful for the comments and instructions from the previous four reviewers. We have carefully revised the manuscript for grammatical issues. Many thanks to the suggestions from Reviewer 2.

Reviewer #5 (Remarks to the Author):

In the revised Manuscript "The transient liver steatosis translationally controls the epigenetic regulator MIER1 to promote liver regeneration" Chen et al. significantly enhance their findings by providing a considerable effort to address most of the reviewers concerns. They optimized their experimental design, improved their figures,

and designed interesting new experiments. They modified their manuscript accordingly. In general, it is a very interesting work, with novel findings that might have the potential to further our knowledge on human liver transplant and regeneration. This potential translation to the field of human liver transplant/regeneration is interestingly addressed using organoid models but unfortunately this part of the article is weak with several overstatements at that point. First bright-field images of the organoid structures do not match the images of the previously described model and the total absence of characterization (staining for classical markers, hepatocyte functional assays...) makes it difficult to assess the quality of the organoid generation process. Moreover, since authors did not used a shMIER1 inducible system and provided very few images, it is very difficult to understand if the observed difference in organoid size is due to a difference in hepatocyte proliferation or rather to immature cells in the first steps of the protocol. A proper characterization of the day 24 hepatocyte organoid and a Ki67 staining/quantification might help enforce the data. Finally, the mRNA expression of a few markers of hepatocyte differentiation is too preliminary to state that MIER1 depletion does not affect human liver characteristics. Again, while I acknowledge that this is not the main part of the article but rather a way to open the study for future applications in human care, a minimal characterization of the organoid model and its evolution upon MIR1 depletion at protein, histological and or functional levels is mandatory to suggest that it might be involved in regeneration processes in human and might not affect the overall liver characteristics.

Response: We appreciate very much the suggestions from the reviewer, and agree with the reviewer that the original characterizations of HLOs were indeed not sufficient. To address this issue, we performed the following additional assays:

1) we further analyzed the expression change of cell cycle proteins in hPSCs and foregut cells with or without MIER1 depletion. Compared to HLOs, results showed no significant difference in cell cycle marker expression in these two stages upon MIER1 depletion, suggesting that MIER1 mostly functions to improve cell proliferation after the foregut stage (**Figure S3A, S3B, S3C**). However, we are still unclear about whether MIER1 loss promoted the proliferation of hepatic progenitor cells or mature hepatocytes. We acknowledged this limitation in revised manuscript (**Line 205-208**): However, as MIER1 depletion started in hPSCs, whether MIER1 depletion promoted proliferation of hepatic progenitor cells or mature hepatocytes is so far unclear. Deeper

analyses to the function of MIER1 in HLO differentiation and proliferation, as well as in human liver regeneration processes, are warranted in the future before making a conclusion.

2) The organoids were previously cultured in the Corning Elplasia non-adherent surface 96 well plates, and the bright-field images were taken for organoids grown in microcavities in the plates. To get clearer pictures of these spheroids, organoids were grown in Matrigel this time, and the pictures were taken at the HLO stage (**Revised Figure S3D**), which demonstrated larger size spheroids after MIER1 depletion.

3) As suggested, we also did the fluorescence staining of Ki-67 in HLOs (**Revised Figure S3E**), which showed more Ki-67+ cells in HLOs with MIER1 depletion.

4) Albumin secretion and expression of several hepatic markers were also measured in HLOs (**Revised Figure S3F, S3G)**. No obvious difference was observed after MIER1 depletion.

Detailed results can be found in revised Figure S3, or directly accessed below.

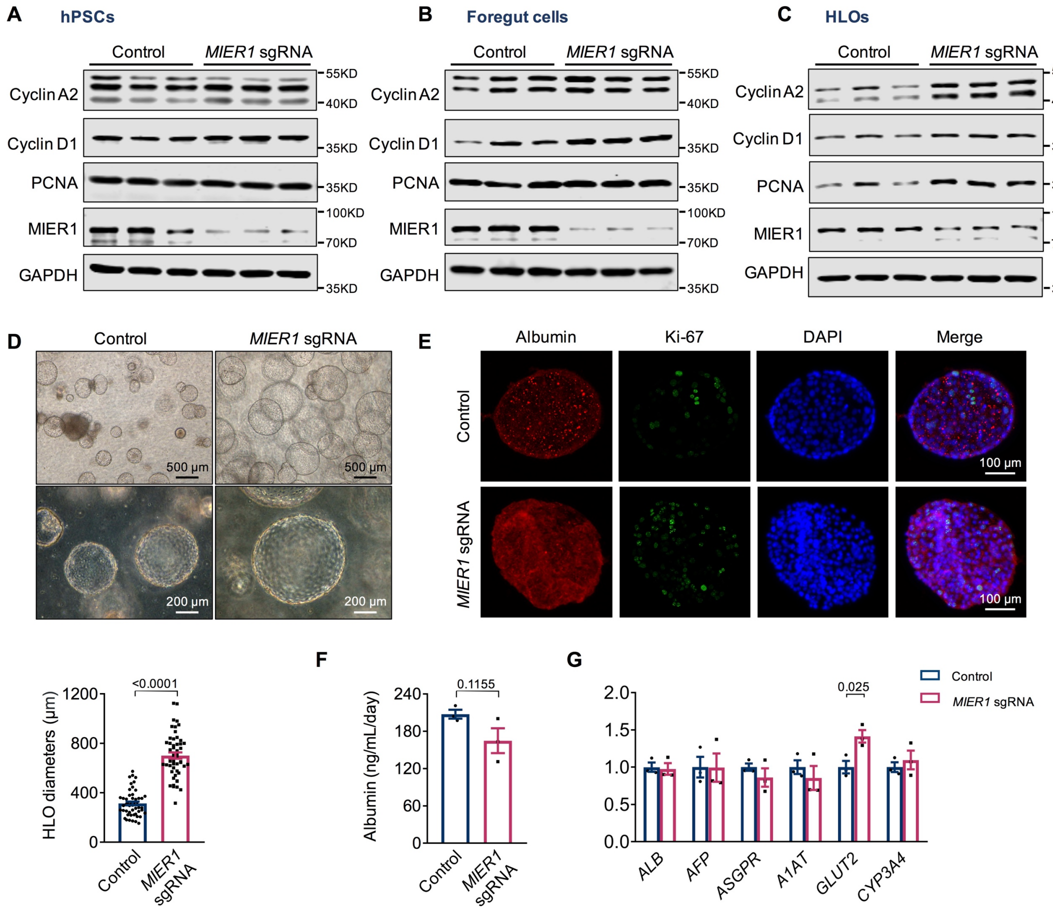

In addition, we recently have a peer-reviewed manuscript accepted in *Cell Regeneration*, in which HLOs were adopted as a cellular model for drug-induced liver injury and for drug screening. The same approach with 5F expansion (designated as 5F-HLO in below) for HLO generation was adopted in these two studies. We have a more detailed characterization of HLOs in that manuscript, and some of the results are shown below for reference.

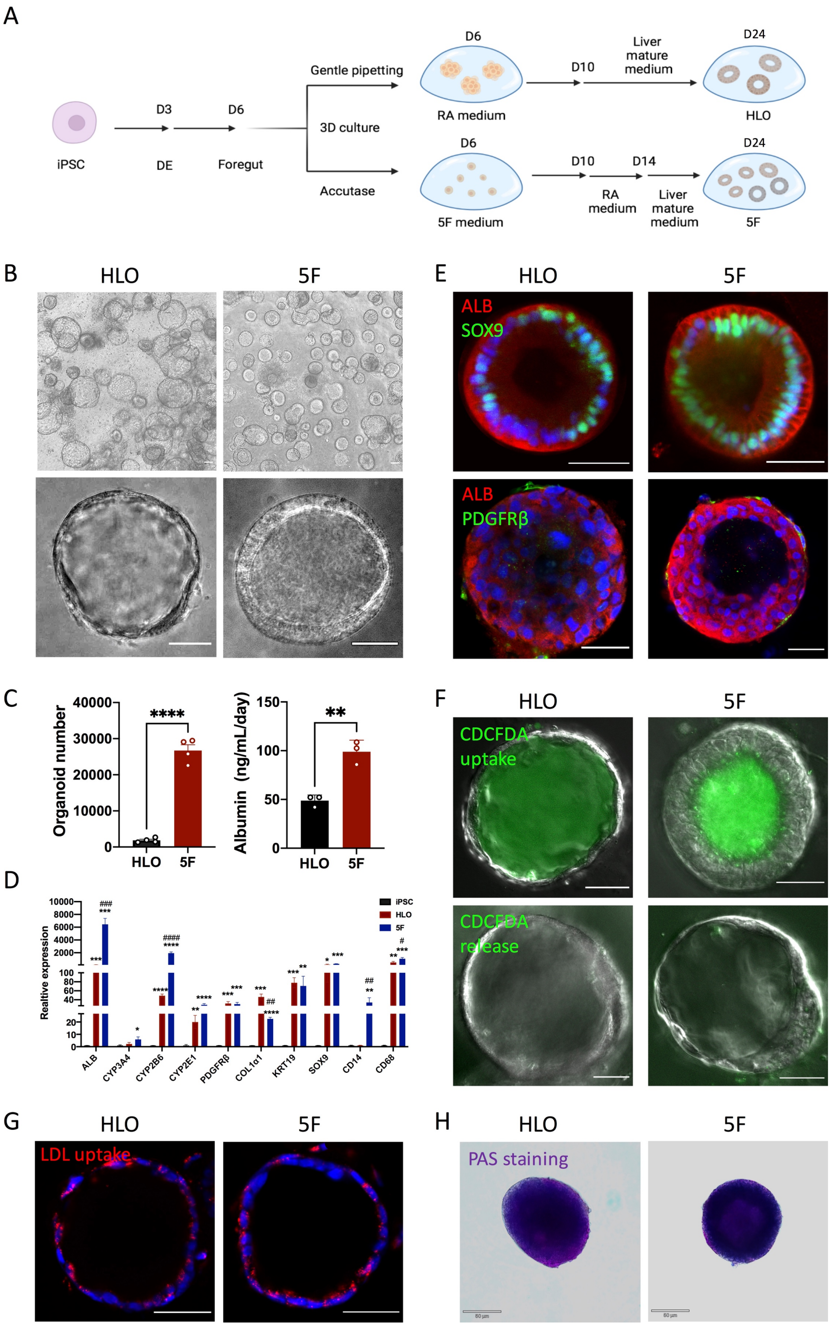

In summary, we appreciate the reviewer's suggestions to improve the characterization of HLOs and the function of MIER1 in HLOs. The current revised results suggest a

possible similar effect of MIER1 depletion in promoting human liver cell proliferation. However, we do recognize the limitations of HLOs to recapitulate the complex *in vivo* liver regeneration process, and the lack of inducible depletion approach to address the effect of MIER1 in different differentiation stages in this study. We stressed the limitations in revised manuscript and stated that the function of MIER1 in HLOs and in human liver regeneration needs more investigation before conclusions can be made.

REVIEWERS' COMMENTS

Reviewer #5 (Remarks to the Author):

In this revised version of the manuscript, the authors addressed all my concerns.

Reviewer #5 (Remarks to the Author):

In this revised version of the manuscript, the authors addressed all my concerns.

Response: We appreciate the previous helpful suggestions from the reviewer.
